# Thrombin Generation Is Associated With Extracellular Vesicle and Leukocyte Lipid Membranes in Atherosclerotic Cardiovascular Disease

**DOI:** 10.1161/ATVBAHA.124.320902

**Published:** 2024-08-01

**Authors:** Majd B. Protty, Victoria J. Tyrrell, Keith Allen-Redpath, Shin Soyama, Ali A. Hajeyah, Daniela Costa, Anirban Choudhury, Rito Mitra, Amal Sharman, Parveen Yaqoob, P. Vince Jenkins, Zaheer Yousef, Peter W. Collins, Valerie B. O’Donnell

**Affiliations:** 1 Systems Immunity University Institute, Cardiff University, United Kingdom (M.B.P., V.J.T., A.A.H., D.C., P.V.J., V.B.O.D.).; 2 Cardiff and Vale University Health Board, Heath Park, Cardiff, United Kingdom (P.V.J.).; 3 Department of Nutritional Sciences, University of Reading, United Kingdom (K.A.-R., S.S., A.S., P.Y.).; 4 Morriston Cardiac Centre, Swansea Bay University Health Board, United Kingdom (A.C.).; 5 Department of Cardiology, University Hospital of Wales, Cardiff, United Kingdom (R.M., Z.Y.).

**Keywords:** acute coronary syndrome, humans, lipidomics, phospholipids, thrombosis

## Abstract

**BACKGROUND:**

Clotting, leading to thrombosis, requires interactions of coagulation factors with the membrane aminophospholipids (aPLs) phosphatidylserine and phosphatidylethanolamine. Atherosclerotic cardiovascular disease (ASCVD) is associated with elevated thrombotic risk, which is not fully preventable using current therapies. Currently, the contribution of aPL to thrombotic risk in ASCVD is not known. Here, the aPL composition of circulating membranes in ASCVD of varying severity will be characterized along with the contribution of external facing aPL to plasma thrombin generation in patient samples.

**METHODS:**

Thrombin generation was measured using a purified factor assay on platelet, leukocyte, and extracellular vesicles (EVs) from patients with acute coronary syndrome (n=24), stable coronary artery disease (n=18), and positive risk factor (n=23) and compared with healthy controls (n=24). aPL composition of resting/activated platelet and leukocytes and EV membranes was determined using lipidomics.

**RESULTS:**

External facing aPLs were detected on EVs, platelets, and leukocytes, elevating significantly following cell activation. Thrombin generation was higher on the surface of EVs from patients with acute coronary syndrome than healthy controls, along with increased circulating EV counts. Thrombin generation correlated significantly with externalized EV phosphatidylserine, plasma EV counts, and total EV membrane surface area. In contrast, aPL levels and thrombin generation from leukocytes and platelets were not impacted by disease, although circulating leukocyte counts were higher in patients.

**CONCLUSIONS:**

The aPL membrane of EV supports an elevated level of thrombin generation in patient plasma in ASCVD. Leukocytes may also play a role although the platelet membrane did not seem to contribute. Targeting EV formation/clearance and developing strategies to prevent the aPL surface of EV interacting with coagulation factors represents a novel antithrombotic target in ASCVD.

**Highlights**

- The aminophospholipid (aPL) profile of membranes from platelets, leukocytes, and extracellular vesicles (EVs) in patients with atherosclerotic cardiovascular disease (ASCVD) is characterized in detail using lipidomics, and EV aPL levels were found to be generally higher in ASCVD groups than in healthy controls.
- Thrombin generation, EV counts, and aPL levels are elevated in EV membranes of patient groups as compared with healthy controls, suggesting these particles interact directly with coagulation factors to drive thrombotic risk in ASCVD.
- Membranes from leukocytes were also suggested to contribute somewhat, although platelet aPL did not appear to drive elevated thrombin generation in patients.
- The EV aPL membrane is proposed as a therapeutic target for dampening thrombotic risk in ASCVD.
- Targeting circulating aPL membrane levels or blocking their interaction with coagulation factors could reduce thrombotic risk in ASCVD.

Atherosclerotic cardiovascular disease (ASCVD) refers to a spectrum of conditions ranging from patients with acute coronary syndrome (ACS), stable coronary artery disease (CAD), and positive risk factors (RFs) that have not yet developed obstructive coronary artery stenosis. Worldwide, ASCVD is responsible for the death of >9 million people annually, with an estimated prevalence of ≈200 million.^1,2^ The most severe form of ASCVD is ACS, which is triggered by atherosclerotic plaque rupture, leading to the activation and recruitment of platelets and leukocytes along with upregulation of TF (tissue factor) expression.^3–10^ This activates coagulation, forming an occlusive arterial thrombus driving ischemia and infarction.^11^ Despite standard treatment with antiplatelet agents, the rates of subsequent strokes, myocardial infarction, and cardiovascular death exceed 10% in the first-year post-ACS diagnosis.^12,13^ This indicates that there are likely to be other modifiable factors involved in ASCVD beyond platelet activity.^12–14^

Thrombin generation requires a procoagulant phospholipid (PL) membrane to allow the assembly of the prothrombinase (FXa [factor Xa]/FVa [factor Va]) complex and other Gla (gamma-carboxyglutamic) domain containing coagulation factors.^15^ This can be provided by the external surface of activated platelets, leukocytes, and extracellular vesicles (EVs).^16,17^ Resting platelet and leukocyte membranes are comprised primarily of phosphatidylcholine on the external side, with the aminophospholipids (aPLs) phosphatidylethanolamine (PE) and phosphatidylserine (PS) being internally facing.^18^ The binding of coagulation factors requires a specific PL composition, which includes the electronegative headgroup of PS, supported by PE, and is dependent on the presence of calcium ions.^19,20^ Native phosphatidylcholine does not facilitate the binding of coagulation factors, and so resting platelets or leukocytes provide minimal support for coagulation reactions. During inflammation or acute trauma/bleeding challenge, platelets and leukocytes become activated, leading to a calcium-dependent translocation of PS/PE to the outside of the cell, mediated by scramblase.^21^ Inflammation also leads to the release of aPL-rich EV. Together, this exposure of aPL to the circulation leads to interactions with coagulation factors via calcium, which are essential for effective coagulation in vivo.^22–24^

Whether the enhanced thrombotic risk in ASCVD is related to altered levels of aPL-mediated thrombin generation on the surface of blood cells is currently unknown. Previous studies have focused on differences in coagulation factor amounts and activity, but there have been no studies focusing on the contribution of the PL membrane to thrombin generation independent of TF or plasma content.^25^ To determine this, we characterized the aPL membrane composition in platelets, leukocytes, and EVs in patients with ACS, CAD, and RF. We assessed the ability of these membranes to support coagulation in vitro compared with healthy controls (HCs) using a thrombin generation assay that uses purified coagulation factors independent of TF.^26–29^ This approach allowed the contribution of the procoagulant membrane to coagulation to be determined specifically.

## MATERIALS AND METHODS

### Study Participants for Clinical Cohort (Blood Samples)

#### Clinical Cohort

Participants were recruited from Cardiff University and Cardiff and Vale University Health Boards. Ethical approval was from Health and Care Research Wales (IRAS [Integrated Research Application System] 243701; REC [Research Ethics Committee] reference 18/YH/0502). Age- and sex-matched individuals were recruited into 1 of the following 4 groups: (1) ACS: participants were identified on in-patient cardiology wards using diagnostic tests (ischemic ECG changes, raised troponin level above normal laboratory-defined range) and clinical assessment by the cardiology team. All were recruited within 48 hours of the index event before any revascularization/angioplasty. (2) Significant CAD: patients attending for an elective coronary angiogram to assess for symptoms of stable angina in the absence of a history of ACS. Coronary angiography demonstrated lesions requiring revascularization on anatomic/physiological criteria as defined by guidelines from the European Society for Cardiology (2018).^30^ (3) Risk factor controls with no significant CAD (RF): this group includes patients attending for a diagnostic coronary angiogram with risk factors for ischemic heart disease (a clinical diagnosis of hypertension requiring therapy, diabetes types 1 or 2, hypercholesterolemia [total cholesterol, >6 mmol/L], smoking, chronic kidney disease stage ≥3, or combination thereof) but whose coronary angiogram demonstrates no significant CAD, defined as not requiring revascularization on anatomic/physiological criteria as per the European Society for Cardiology 2018 guidelines.^30^ (4) HCs: participants had no significant history for ischemic heart disease or its risk factors, were never smokers, and were not on antiplatelet agents, anticoagulants, or statins. They were identified from the workplace or were volunteers from partner studies such as HealthWise Wales.^31^ Clinical characteristics are in Table S1. Inclusion criteria were aged ≥18 years, ACS in ACS group, and no history of ACS in the others. Exclusion criteria were diagnosis of infective endocarditis or atrial fibrillation or inability to consent to study. Overall, 90 participants were recruited: HC, n=24; RF, n=23; CAD, n=19; and ACS, n=24. Blood samples were collected by peripheral venepuncture as outlined below, by 1 individual, and all samples were transferred to the laboratory within 10 minutes. The study design is summarized in Figure S1. Blood cell counts were not available for 2 patients with CAD. In our human study, numbers of males and females in patient groups were not significantly different, when compared with HCs, using the Fisher exact test (Table S1). Data from males and females were combined, and sex differences were not determined in the study due to group sizes being too small. Primary analyses were preplanned, not post hoc, and *P* values are specified on figures where <0.05. Power calculations were not performed due to the lack of relevant primary data with which to perform them.

### Platelet Isolation

Whole blood was taken from using a 21G butterfly needle into a 50-mL syringe containing acid-citrate-dextrose (85 mmol/L trisodium citrate, 65 mmol/L citric acid, and 100 mmol/L glucose) at a ratio of 8.1 parts whole blood to 1.9 parts acid-citrate-dextrose, as described previously,^26^ and centrifuged at 250*g* for 10 minutes at 20 °C. The platelet-rich plasma was collected and centrifuged at 1000*g* for 8 minutes at 20 °C. Platelet-poor plasma was removed and retained for EV isolation. The platelet pellet was resuspended in Tyrode buffer (134 mm NaCl, 12 mm NaHCO_3_, 2.9 mm KCl, 0.34 mm Na_2_HPO_4_, 1.0 mm MgCl_2_, 10 mm HEPES, and 5 mm glucose, pH 7.4) containing acid-citrate-dextrose (9:1, v/v). The platelets were washed by centrifuging at 1000*g* for 8 minutes at 20 °C and then resuspended in Tyrode buffer at 2×10^8^/mL^1^. Platelets were activated at 37 °C in the presence of 1 mmol/L CaCl_2_, 0.2 U/mL^1^ thrombin (Sigma-Aldrich).

### Leukocyte Isolation

Leukocytes were isolated from 20 mL citrate-anticoagulated whole blood as described previously.^26^ Briefly, 20 mL of blood was drawn using a 21G butterfly needle into a 50-mL syringe containing 4 mL of 2% citrate and 4 mL of Hetasep (Stem Cell Technologies) and allowed to sediment for 45 minutes. The upper plasma layer was recovered and centrifuged at 250*g* for 10 minutes at 4 °C. The pellet was resuspended in ice-cold 0.4% trisodium citrate/PBS and centrifuged at 250*g* for 5 minutes at 4 °C. Erythrocytes were removed by hypotonic lysis (0.2% hypotonic saline) before being neutralized with a PBS wash. Leukocytes were resuspended in Krebs buffer (100 mmol/L NaCl, 48 mmol/L HEPES, 5 mmol/L KCl, 1 mmol/L sodium dihydrogen orthophosphate dehydrate, and 2 mmol/L glucose) at 4×10^6^/mL. For activation, 4×10^6^ leukocytes were incubated at 37 °C with 10 μmol/L A23187 and 1 mmol/L CaCl_2_, for 30 minutes, before lipid extraction.

### EV Isolation for Clinical Assay

Methods were adapted from recent literature and guidelines.^32,33^ Platelet-poor plasma generated as above was centrifuged at 1000*g* for 10 minutes at 20 °C to generate platelet-free plasma (PFP). One milliliter of PFP was snap-frozen on dry ice and stored at −80 °C for quantification at a later date as below. For each donor’s plasma, 6×1 mL PFP aliquots were centrifuged at 16 000*g* for 30 minutes at 20 °C. Seven hundred fifty microliters was removed from each aliquot, and 750 μL of modified Tyrode buffer was added to the pellet, which was gently resuspended using a pipette. Following a second centrifugation as above, 950 μL was removed. Fifty microliters of modified Tyrode buffer was added to the pellet to gently resuspend and recover the EV-rich fraction. EV fractions were pooled to generate 1 isolate per donor. Of this, 250 μL was used for lipidomics, and 3×20 μL for prothrombinase assays.

### EV Quantification for Clinical Assay

EV quantification was performed by thawing 1 aliquot of PFP per patient, of which 500 μL was passed through size exclusion chromatography iZON qEV columns (Izon Science, Ltd, United Kingdom) to recover particles and vesicles between 70 and 1000 nm in diameter as outlined in the manufacturer’s information. The eluting EV-rich fractions were collected and analyzed using nanoparticle tracking on NanoSight 300 (Malvern, United Kingdom) equipped with a sensitive sCMOS (scientific complementary metal–oxide–semiconductor) camera and a 488-nm blue laser, to generate an EV count and size distribution for all participants. For 2 samples, a measure of count and size could not be obtained due to turbidity.

### ELISA

Commercially available ELISA kits were purchased (Abcam, United Kingdom) for the measurement of apoB (ab190806), d-dimer (ab260076), and human thrombin-antithrombin (TAT) complexes (ab279724) on frozen plasma samples. These were quantified on a plate reader for absorbance. For all available information on the kits and antibodies used, please see the following links:

https://www.abcam.com/ps/products/190/ab190806/documents/Human-Apolipoprotein-B-elisa-kit-protocol-book-v3-ab190806%20(website).pdf and https://www.abcam.com/ps/products/260/ab260076/documents/Human-D-Dimer-ELISA-Kit-protocol-book-v3a-ab260076%20(website).pdf.

### Testing for Lipoprotein Contamination EV Isolated by Size Exclusion Chromatography

Twelve male and female subjects (females, 10; males, 2; age, 22–37 years) were recruited from the University of Reading. All subjects gave informed consent. Ethical approval for the study was obtained from the School of Chemistry, Food and Pharmacy Ethics Committee at the University of Reading (study number 17/17) and conducted according to the guidelines laid down in the Declaration of Helsinki. The inclusion criteria comprised the following: age range 18–65 years, nonsmoker, hemoglobin ≥115 g/L for women and 130 g/L for men, total cholesterol <5 mmol/L, TAG 0.4 to 1.5 mmol/L, no disclosed history of drug or alcohol abuse, and no illness or disease requiring medication (excluding hormone replacement therapy, oral contraceptive, and thyroxine replacement therapy). Participants were excluded if unwell or on any prescribed medication and were asked to avoid exercise, alcohol, and fatty meals the day before the experimental visit, consuming a low-fat meal the evening before the visit. Participants attended the Hugh Sinclair Unit of Human Nutrition following a 12-hour overnight fast, and a baseline fasting blood sample was taken before consuming a high-fat test meal consisting of 2 all-butter croissants containing 24 g fat, 10.6 g protein, and 48 g carbohydrate. A second blood sample was collected 4 hours later, corresponding to the point at which postprandial lipemia reaches a peak. Venous blood samples were drawn into citrated tubes, inverted 4×, and processed immediately. Samples were centrifuged at 1500*g* at room temperature for 15 minutes to remove larger cells and cellular debris. Further centrifugation at 13 000*g* for 2 minutes at room temperature produced PFP, which was aliquoted and stored at −80^ ^°C for further analysis.

1. Isolation of EVs using size exclusion chromatography (SEC): PFP (0.5 mL) was thawed at room temperature on a sample roller and loaded onto a qEV original column (Izon, Oxford, United Kingdom), which had been preflushed with 30 mL PBS. A further 5 mL PBS was passed through the column to elute EVs based on their size and 0.5 mL fractions were collected.
2. Lipoprotein isolation: density gradient centrifugation was performed on baseline and postprandial PFP to isolate chylomicrons, VLDL (very-low-density lipoprotein)-1, and VLDL-2, and the fractions were stored at −20 °C in the presence of apo B_48_ preservative (5% v/v) for later analysis, as described in the study by Palmer et al.^34^
3. Assessment of contamination of EV fractions with apo B_48_ and apo B_100_: to determine whether EV fractions prepared from PFP by SEC were contaminated with chylomicrons or VLDL, EV fractions prepared from both baseline and postprandial samples of PFP were subjected to ELISA for apo B_48_ (chylomicrons [CM]) and apo B_100_ (VLDL). The presence of CM was evaluated using the Human Apolipoprotein B48 ELISA kit purchased from ElabScience (Houston, TX), and the presence of VLDL was investigated using the human apo B_100_ ELISA kit (Sigma-Aldrich, St. Louis, MO). The ELISA tests were performed according to the manufacturer’s instructions and read on a plate reader (Spark, Tecan, United Kingdom).
4. Assessment of the potential coisolation of lipoproteins prepared by density gradient centrifugation with EVs: CM, VLDL-1, and VLDL-2 fractions prepared by density gradient centrifugation (as described above) were subjected to SEC to determine whether there was co-elution of lipoproteins and EVs. Fractions were analyzed using nanoparticle tracking analysis.

### Prothrombinase Assay

Resting platelets (4×10^6^), resting leukocytes (8×10^4^), and plasma EV isolates (20 μL) were added in triplicate to a 96-well half-area flat bottom clear plate (Greiner, Austria). Next, a mix of recombinant FXa (50 nmol/L; Enzyme Research Laboratories, United Kingdom), FVa (15 nmol/L; Haematologic/Cambridge Bioscience, United Kingdom), FII (factor II; 1 μmol/L; Enzyme Research Laboratories, United Kingdom), and CaCl_2_ (5 mmol/L) in prothrombinase buffer (20 mmol/L Tris, 150 mmol/L NaCl, and 0.05% BSA w/v) was added to the wells and the reaction allowed to proceed for 5 minutes at 21 °C, before being quenched with an excess of EDTA (7 mmol/L final concentration). Thrombin (FIIa) activity was measured on a plate reader using a chromogenic substrate S-2238 (Enzyme Research Laboratories, United Kingdom). A graphical depiction of the assay is shown in Figure S2.

### Lipid Biotinylation, Extraction, and Analysis for aPL

To determine the amounts of PE and PS on the external leaflet of cell membranes, total and external aPLs were measured as described previously.^35^ Briefly, 0.2 mL of platelets (4×10^7^), leukocytes (8×10^5^), or EVs isolated as described above were incubated with 20 μL of 20 mmol/L NHS (National Health System)-biotin for 10 minutes at 21 °C to label total aPL. In the case of externalized aPL, samples were incubated with 86 μL of 11 mmol/L EZ-Link sulfo-NHS-biotin for 10 minutes at 21 °C followed by 72 μL of 250 mmol/L of L-lysine for 10 minutes at 21 °C. The final volumes were made up to 0.4 mL with PBS. Lipids were extracted by adding samples to 1.5 mL chloroform:methanol (1:2) containing 10 ng internal standards (biotinylated 1,2-dimyristoyl-PE and biotinylated 1,2-dimyristoyl-PS, generated as in the study by Thomas et al^35^) to give a solvent:sample ratio of 3.75:1, as described previously.^35^ Following vortexing and centrifugation (400*g*, 5 minutes), lipids were recovered in the lower chloroform layer, which was dried under vacuum. Samples were analyzed for aPL using liquid chromatography tandem mass spectrometry (LC/MS/MS). For this, samples were separated on the Ascentis C-18 (5 µm 150 mm×2.1 mm) column (Sigma-Aldrich) with an isocratic gradient (methanol, 0.2% w/v NH_4_CH_3_CO_2_) at a flow rate of 400 μL/min. Products were analyzed in multiple reaction monitoring mode on a Q-Trap 4000 instrument (Applied Biosystems, United Kingdom) by monitoring precursor-to-product ion transitions in negative ion mode (Table S2). The peak area for the analytes was integrated and normalized to the internal standards. Limit of quantitation is defined as signal:noise ratio of 5:1 with at least 6 data points across a peak. For quantification, standard curves were generated using biotinylated PS and PE.^35^ Chromatograms of biotinylated PS and PE species as detected in representative participant samples can be seen in Figures S3 and S4, respectively. For 5 patient extracts processed on the same day, internal standard could not be detected during subsequent LC/MS/MS and they were excluded. In a further 8 samples, while conducting LC/MS/MS analysis of the full cohort samples, we identified that a batch of NB (NHS-biotin) used for this subset of samples had been inactive. SNB (sulfo-NHS-biotin) used for those samples was active, so externalized but not total PE/PS levels were determined for these samples. Full data on all sample numbers are included in legends for clarity.

### Statistical Analysis

Statistical significance was determined using 1-way ANOVA with Tukey post hoc test (astatsa.com). For platelets and leukocytes, resting or activated samples were compared separately. Correlation analysis utilized Pearson correlation for linear dependence between variables. The cutoff value chosen for test significance was *P*<0.05. Box plots were drawn in Excel (Microsoft) with edges indicating the interquartile range, the line inside the box indicating the median, and whiskers indicating 1.5× the interquartile range. For generation of heatmaps, samples were averaged within their groups, and a log10 was applied to lipid amounts (ng) normalized to cell count or tissue weight (mg) for each lipid to allow row-wise and column-wise comparison. Next, lipid measurements were plotted as intensity values using the pheatmap package in the R coding environment (v3.6.2, open source) with lipid hierarchical clustering. Intensity levels were represented by a color gradient ranging from blue (low levels or absent) to red (high levels) with variations in between. Graphical illustrations of assays, designs, and pathways were performed using the online platforms draw.io and biorender.com (premium subscription). For lipidomics data statistical analysis, missing values where lipids were below the limit of quantitation were replaced with 50% of the assay limit of quantitation value. If any lipid was missing >50% in a sample set, it was not analyzed. Where values were imputed, they are shown in red in the Data Set in the Supplemental Material.

### Data Availability

The authors declare that all supporting data are available within the article and its Supplemental Material. Raw data for individual experiments are available from the corresponding authors on reasonable request.

## RESULTS

### EV-Containing Plasma From Patients With ACS Supports Elevated Thrombin Generation, Driven by Higher EV Counts in Disease

EV fractions isolated using either centrifugation or SEC were found to be free of lipoproteins as measured using the ab190806 human apoB ELISA kit (Abcam, United Kingdom; Figure S5A) or by monitoring lipoprotein elution from SEC (Figure S5B through S5F). Here, when PFP was subjected to SEC, EVs eluted in fractions 7 to 9. However CMs (apo B_48_) and VLDLs (apo B_100_) in PFP only began to elute from fraction 11 onward (Figure S5B and S5C), demonstrating that lipoproteins elute later than EVs, with little cross-contamination. This is consistent with lipoproteins having a smaller diameter than EVs. Lipoproteins isolated first by density gradient centrifugation and then subjected to SEC did not elute in EV enriched fractions (fractions 7–9) and began to elute in the later fractions (Figure S5D through S5F). Specifically, CM isolated from fasted plasma eluted after fraction 12 and those isolated from postprandial plasma eluted after fraction 10, indicating that the postprandial CM population was larger in diameter but still distinct from the EV population. The VLDL-1 population eluted after fraction 14 and the VLDL-2 population eluted after fraction 16, indicating the progressively smaller diameters of these lipoproteins.

**Figure 1.**
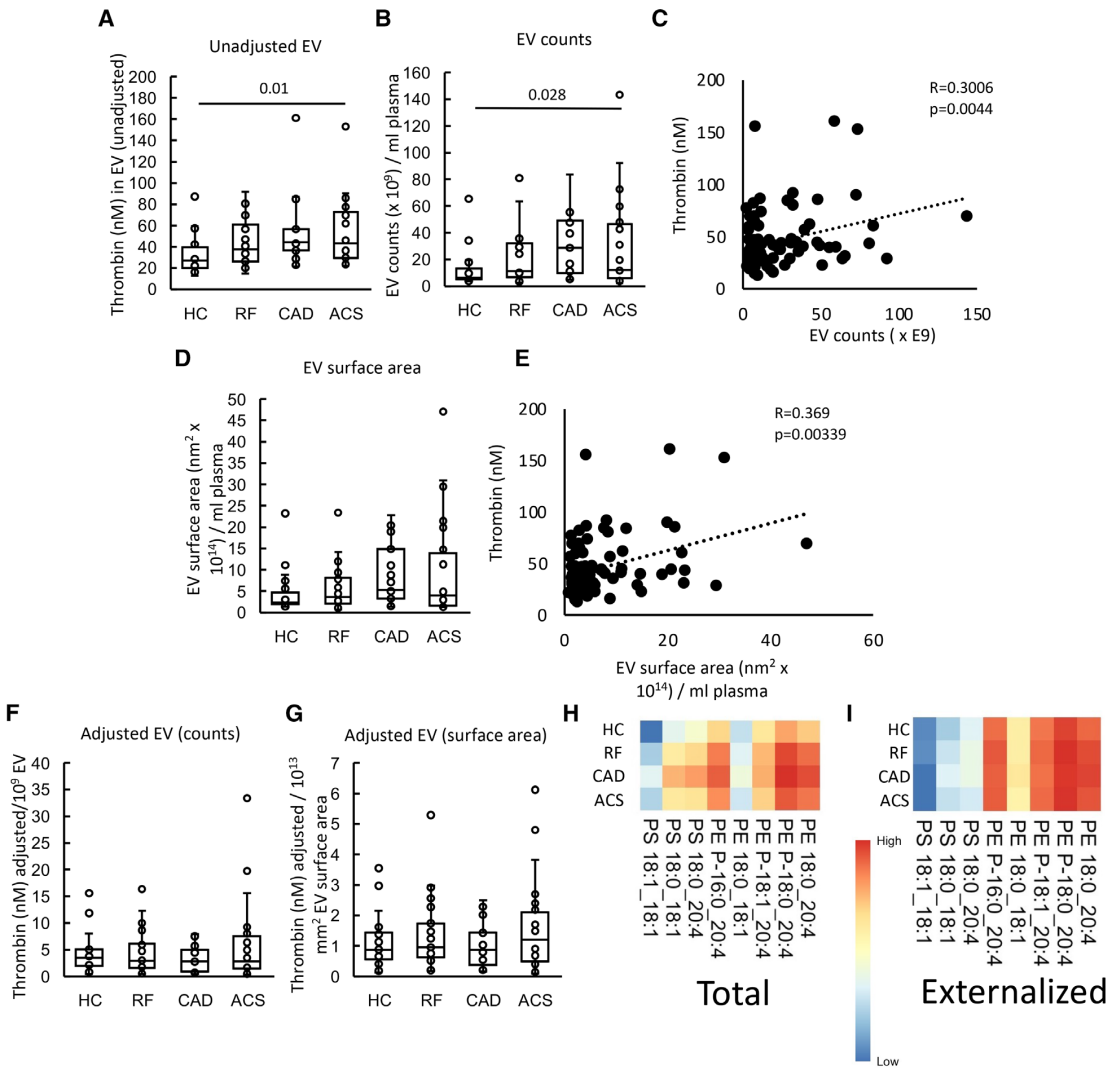
**Plasma extracellular vesicles (EVs) support elevated thrombin generation in atherosclerotic cardiovascular disease (ASCVD) plasma. A**, EVs from patients support higher levels of thrombin generation. The ability of EV membranes to support thrombin generation was assessed using prothrombinase assay as described in Materials and Methods. **B**, EV counts are significantly higher in patients. To quantify EV, platelet-free plasma (0.5 mL) was processed using size exclusion chromatography (iZON qEV columns) and nanoparticle tracking analysis (Nanosight 300). **C**, Thrombin generation positively correlates with EV counts. Thrombin generation and EV counts were correlated using Pearson correlation. **D**, EV surface area is increased in vascular disease. EV surface area was calculated as described in Materials and Methods and plotted as box plots. **E**, Thrombin generation positively correlates with EV surface area. Thrombin generation and EV surface area were correlated using Pearson correlation. **F** and **G**, Thrombin generation was not changed between groups if EVs were normalized by counts or surface area. Thrombin generation on the surface of EV was adjusted by EV counts (per 1×10^9^ EV; **F**) and surface area (**G**) and plotted as box plots. **H** and **I**, Heatmaps showing aminophospholipid (aPL) molecular species in EV. Heatmaps were drawn using the pheatmap R package as described in Materials and Methods to visualize aPL amounts between groups for all the measured species, analyzed using liquid chromatography tandem mass spectrometry. Statistical significance was tested with 1-way ANOVA and Tukey post hoc test (*P*<0.05 considered significant). Acute coronary syndrome (ACS; n=24: **A**, **B**, **D**, **F**, and **G**; n=21: **H** and **I**), coronary artery disease (CAD) but no ACS (n=19: **A**, **B**, **D**, **F**, and **G**), positive risk factors (RFs) with no significant CAD (n=23: **A**, **B**, **D**, **F**, and **G**; n=22: **H** and **I**), and healthy control (HC; n=24: **A**; n=22: **B**, **D**, **F**, and **G**; n=23: **H** and **I**).

In an in vitro system using purified FXa, FVa, and FII, EV-containing plasma from patients with ACS stimulated significantly higher thrombin generation than those from HCs, while there was a higher trend for RF and CAD (Figure 1A). EVs were isolated from a fixed volume (6 mL) of plasma; thus, particles were quantified using nanoparticle tracking analysis. There was a trend for higher EV counts in plasma from all patient groups compared with HC, which was significantly higher for CAD (Figure 1B). Furthermore, there was a significant weak positive correlation between thrombin generation and EV counts (Figure 1C). The mean EV vesicle diameter was significantly smaller in RF and CAD and slightly smaller in ACS, compared with HC (Figure S6). As coagulation takes place on the surface of vesicles, we next calculated the EV total surface area (vesicle area as a sphere calculated as 4πr^2^×EV counts) in plasma. A trend was seen for higher EV total surface areas in patient groups although they showed greater overall variability (Figure 1D). Thrombin generation correlated significantly with EV total surface area indicating a direct relationship between EV membrane and coagulation (Figure 1E). Overall, this indicates that EVs from ASCVD patient groups (RF, CAD, and ACS) tend to be smaller than HCs, but due to higher counts, the overall EV surface area is elevated in disease groups and will directly contribute to elevated thrombin generation observed. Confirming this idea, we normalized thrombin generation to either EV counts or surface area and found that this abolished the differences between groups (Figure 1E and 1F).

### Characterization of aPL Molecular Species in EV Shows Levels Tend to Be Higher in Disease Groups

The aPL composition of EVs has not been described before; hence total and external PS and PE species were next characterized using LC/MS/MS. In this assay, external facing aPLs are derivatized using the cell impermeable biotinylation reagent, sulfo-NHS-biotin. Total aPL is instead derivatized using the cell-permeable form, NHS-biotin.^35^ Derivatized aPL are then detected using LC/MS/MS, based on a mass shift of +226 atomic mass units from the native lipid. This method was previously used to determine the molecular species and amounts of PS and PE on the surface of platelets from healthy donors.^27^ The most abundant species detected were as follows: PE P-16:0_20:4, PE 18:0_20:4, PE P-18:0_20:4, PS 18:0_18:1, and PS 18:0_20:4 (Figure 1G; Figure S7A), as previously shown in healthy platelets.^27^ Externalized PS and PE comprised the same molecular species, with the most abundant isomers being detected in higher amounts on the outside (Figure 1H; Figure S7B). This indicates an absence of selectivity for any particular aPL isomers to be present on the outer side of EV.

**Figure 2.**
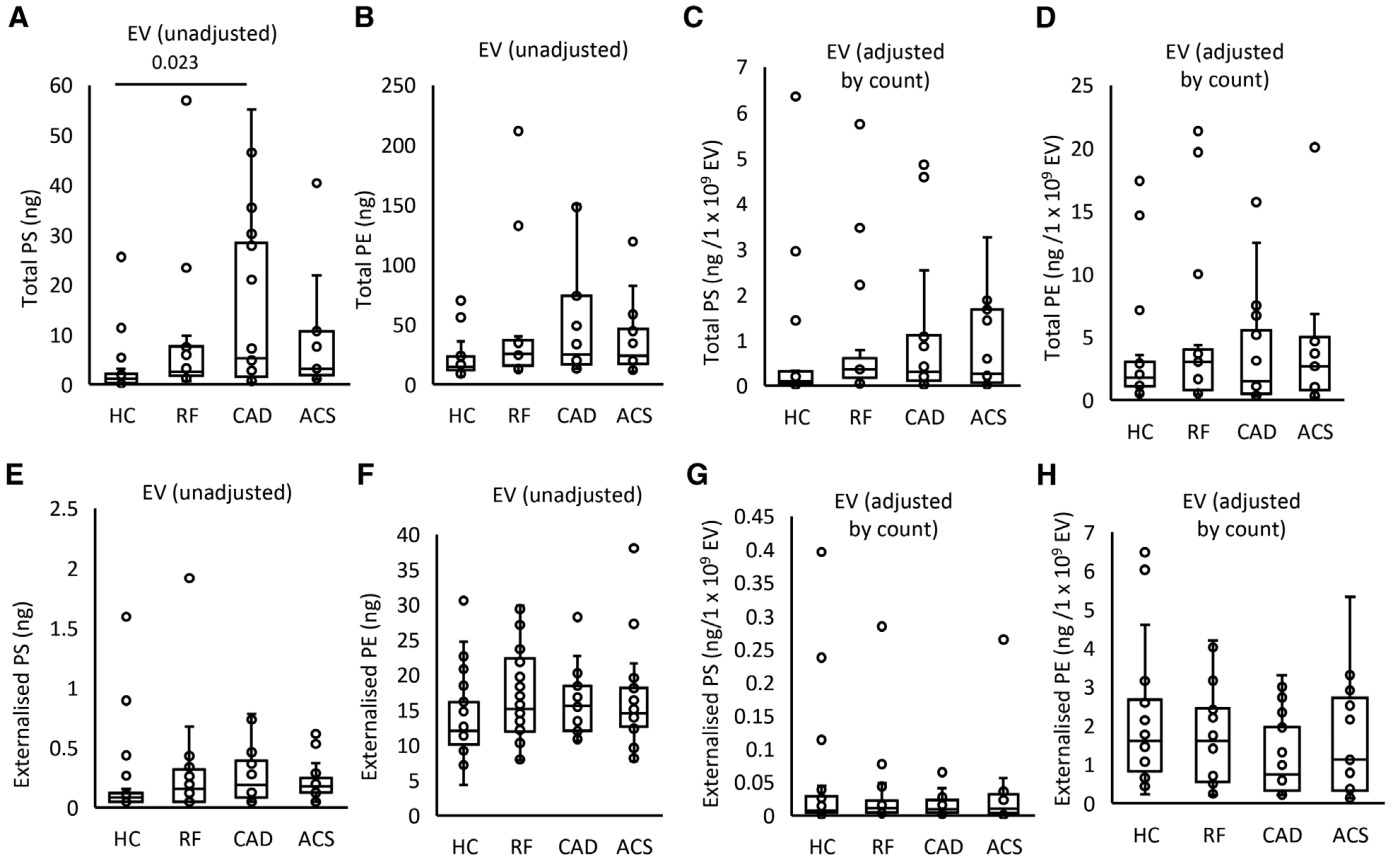
**Levels of externalized phosphatidylethanolamine (PE) and phosphatidylserine (PS) on extracellular vesicles (EVs) reflect higher EV counts in patients. A** and **B**, Grouping by headgroup, the amounts of total PS and total PE for each of the clinical groups were plotted to examine for differences between groups. **C** and **D**, Total aminophospholipid (aPL) amounts adjusted by EV count. **E** and **F**, Grouping by headgroup for externalized PS and PE lipids. **G** and **H**, Externalized aPL amounts adjusted by EV count. Lipids were extracted from EV-rich plasma fractions as in Materials and Methods. Lipid amounts (ng) were calculated by liquid chromatography tandem mass spectrometry. Statistical significance was tested with 1-way ANOVA and Tukey post hoc test (*P*<0.05 considered significant). Acute coronary syndrome (ACS; n=19: **A–D**; n=21: **E–H**), coronary artery disease (CAD) but no ACS (n=18: **A–D**; n=19: **E–H**), positive risk factors (RFs) with no significant CAD (n=17: **A–D**; n=22: **E–H**), and healthy control (HC; n=23: **A**, **B**, **E**, and **F**; n=22: **C**, **D**, **G**, and **H**).

EVs from plasma of patients with CAD contained higher levels of total PS and total PE, with a trend for higher levels in all patient groups, compared with HC (Figure 2A and 2B); however, once data were normalized to EV counts, this disappeared indicating that it was due to higher EVs being present in plasma from patients with ACS and CAD (Figure 2C and 2D). External PS amounts significantly correlated with thrombin generation on EVs in line with a functional involvement in driving coagulation (Figure S8A). Externalized PS showed a nonsignificant trend to be higher in all patient groups versus HCs, although externalized PE was similar (Figure 2E and 2F). Once adjusted by EV count, externalized aPL trends disappeared (Figure 2G and 2H). These data suggest that EVs drive coagulation via their external PS, which may be somewhat elevated in ASCVD groups compared with HCs.

### Thrombin Generation in Leukocytes From Patients With ACS Is Slightly Higher Than HC

**Figure 3.**
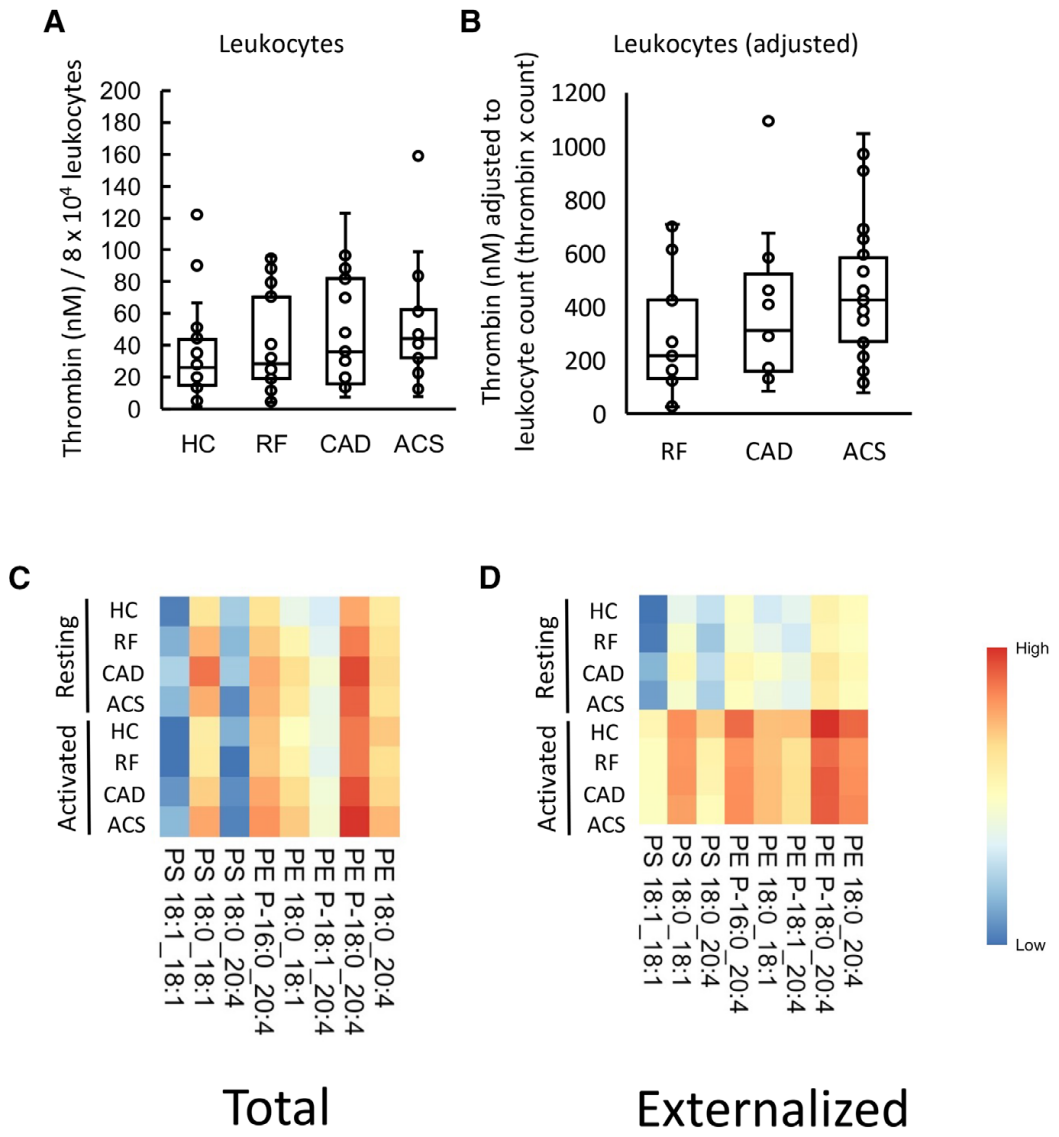
**Leukocytes from patients with acute coronary syndrome (ACS) generated more thrombin than healthy controls (HCs), and this may be further increased by higher in vivo leukocyte counts. A**, Leukocytes from patients with ACS support higher levels of thrombin generation than HCs. The ability of leukocyte membranes to support thrombin generation was quantified using prothrombinase assay as described in Materials and Methods and displayed on a box plot. **B**, Adjusting by total leukocyte count demonstrates an upward trend in thrombin generation among positive risk factor (RF), coronary artery disease (CAD), and ACS samples. Thrombin generation was adjusted by total leukocyte count to account for differences between groups. **C** and **D**, Heatmaps show aminophospholipid (aPL) molecular species in leukocytes, with increased externalization upon activation. Lipids were extracted from resting or ionophore-activated leukocytes and quantified using liquid chromatography tandem mass spectrometry as described in Materials and Methods. The log10 lipid amounts (ng) were plotted on a heatmap using the pheatmap R package as described in Materials and Methods to show total (**C**) and externalized (**D**) aPL molecular species. Statistical significance was tested with 1-way ANOVA and Tukey post hoc test (*P*<0.05 considered significant). ACS (n=24: **C**; n=21: **D**), CAD but no ACS (n=19: **A**; n=17: **B**), RFs with no significant coronary artery disease (n=23: **C**; n=22: **D**), and HC (n=24: **A**; n=23: **C** and **D**).

Next, we characterized the procoagulant membrane on circulating white cells from patient groups, also comparing their ability to support thrombin generation with PE/PS content. Resting leukocytes (8×10^4^) from patients showed a trend to support higher thrombin generation than HCs, in particular for patients with ACS (Figure 3A). In ASCVD groups, there were significantly higher circulating leukocyte counts as measured by the hospital clinical laboratory, with worsening disease, although all cell counts were within normal range (Table S1). To test the potential impact of cell count in vivo, thrombin generation was normalized by circulating leukocyte count, since a higher count in disease could further impact thrombin generation. This further demonstrated an upward trend with higher amounts of thrombin predicted to be generated in ACS compared with RF (Figure 3B). Leukocyte counts had not been obtained for HC, as volunteers were outside the hospital system; so direct comparison with this group was not possible.

### Leukocytes Externalize aPL on Activation, Although Molecular Composition Is Unchanged in CAD

**Figure 4.**
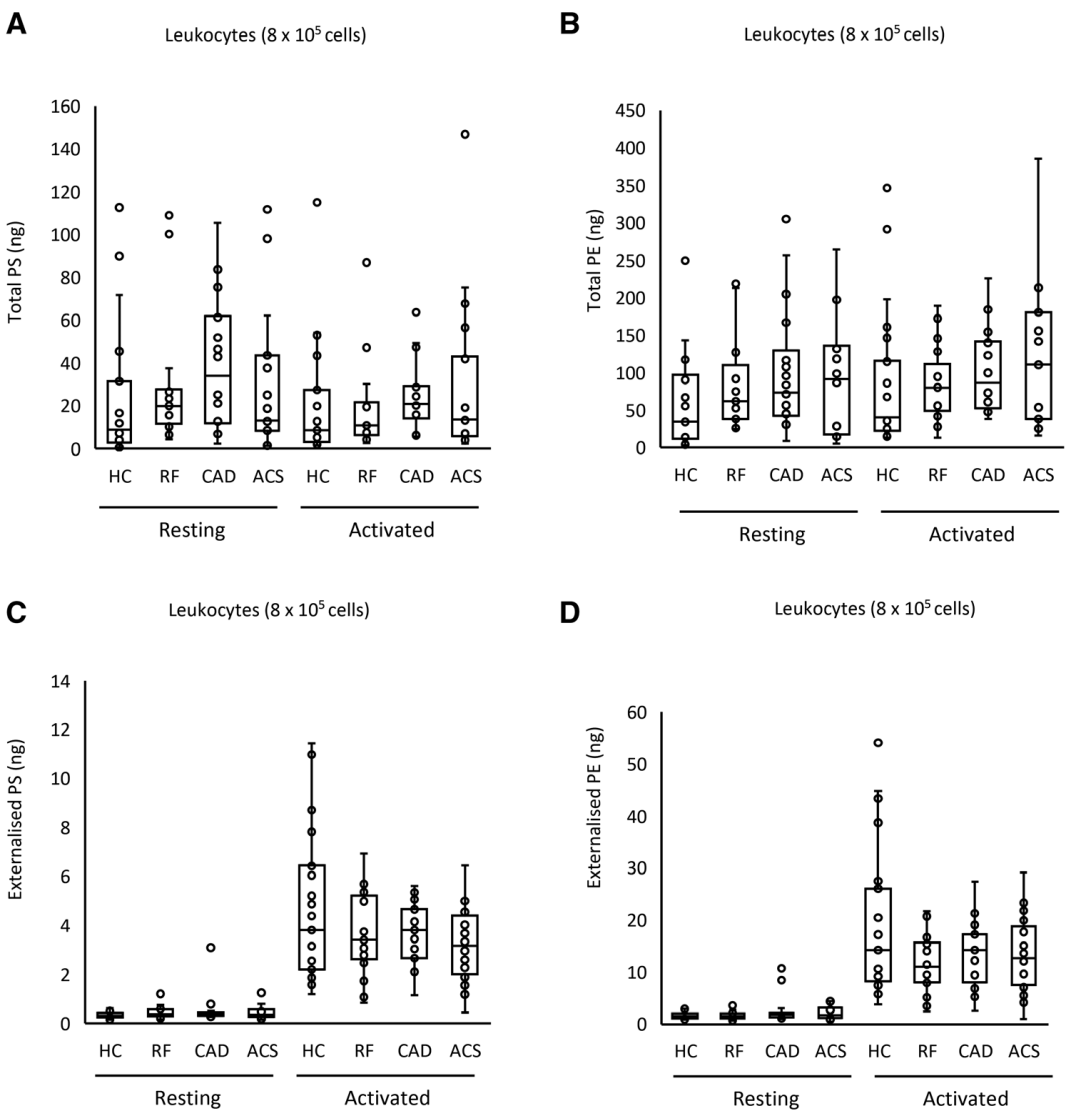
**Leukocytes from patients with acute coronary syndrome (ACS) contain and externalize similar amounts of phosphatidylethanolamine (PE)/phosphatidylserine (PS), when expressed on a per-cell basis. A** through **D**, Lipids were extracted from resting or ionophore-activated leukocytes as described in Materials and Methods. Lipid amounts (ng) were determined using liquid chromatography tandem mass spectrometry. Statistical significance was tested with 1-way ANOVA and Tukey post hoc test (*P*<0.05 considered significant). ACS (n=19: **A** and **B**; n=21: **C** and **D**), coronary artery disease (CAD) but no ACS (n=18: **A** and **B**; n=19: **C** and **D**), positive risk factors (RFs) with no significant CAD (n=17: **A** and **B**; n=22: **C** and **D**), healthy control (HC; n=23).

The aPL composition of leukocyte membranes is currently unknown. To characterize this, and determine the impact of ASCVD, total and external molecular species of aPL were quantified in leukocytes, both before after calcium ionophore activation. Activation reveals the potential of the cells, when fully activated, to externalize aPLs, while resting cells are more comparable with circulating cells. Three PS and 5 PE species were detected, with all PEs containing 20:4, and of a similar composition to EV (Figure 3C and 3D; Figure S9A). Total PE and PS was similar for all groups, and this was not impacted by activation (Figure 4A and 4B; Figure S9B). In contrast, following ionophore activation, leukocytes from all groups externalized aPLs (Figure 4C and 4D). The most abundant aPL isomers were detected in higher amounts on the outside indicating a lack of selectivity for any specific aPL isomer to be externalized in leukocytes (Figure 4C and 4D; Figure S9A). Externalized aPL species or amounts showed only small differences between patient groups, and there was no significant correlation between the amount of thrombin generated on the surface of resting leukocytes and externalized PS and PE amounts (Figure 4C and 4D; Figure S8B). These data indicate that the elevated thrombin generation on the surface of leukocytes in ACS compared with HC is unlikely to be related to the levels of externalized aPL.

### Thrombin Generation on the Surface of Resting Platelets Was Similar for All Groups

**Figure 5.**
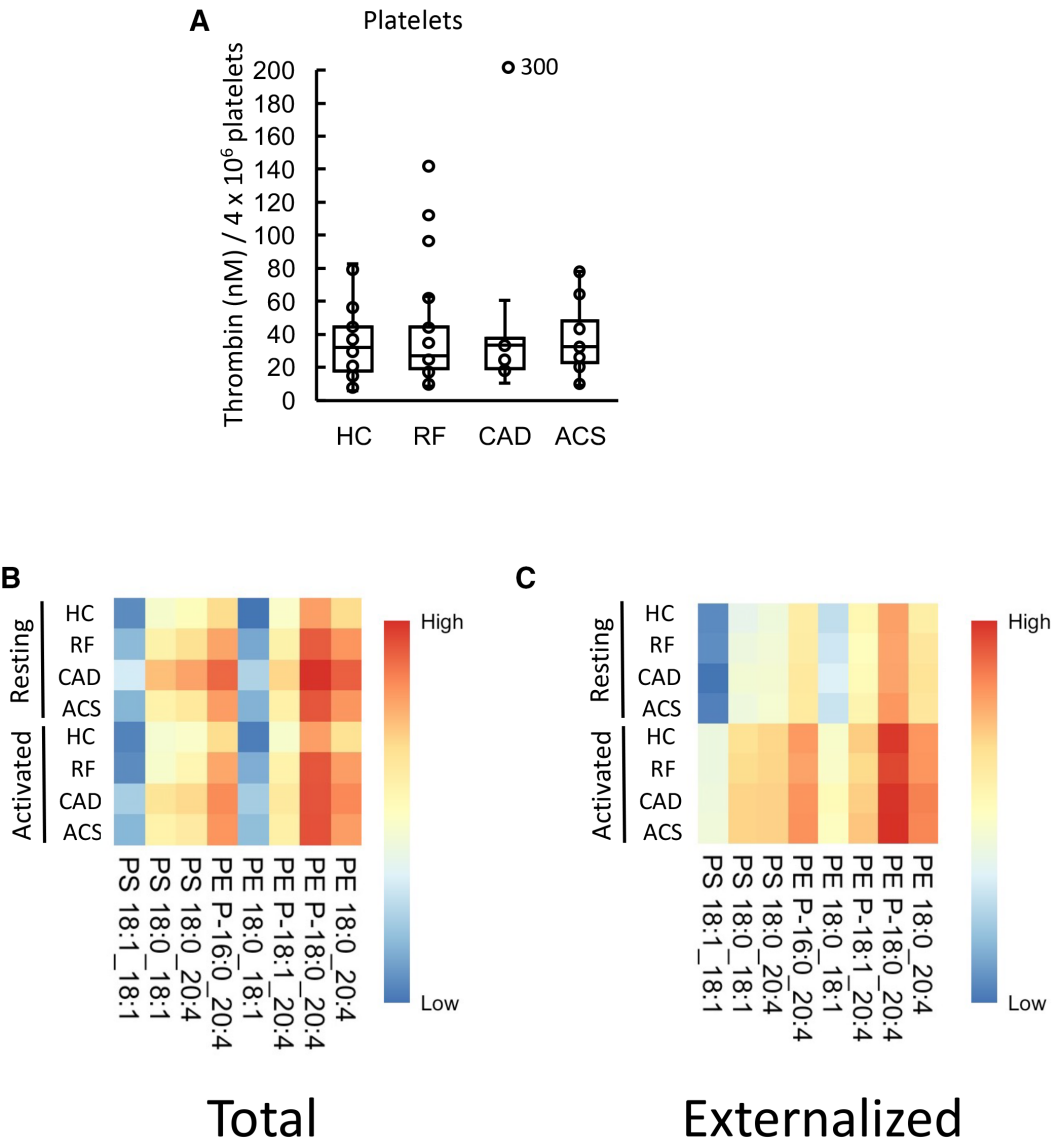
**Thrombin generation on the surface of atherosclerotic cardiovascular disease (ASCVD) platelets was unchanged from healthy controls (HCs), and there were minimal differences in aminophospholipid (aPL) externalization between groups. A**, Thrombin generation on the platelet surface was similar between clinical groups. The ability of platelet membranes to support thrombin generation was assessed using the prothrombinase assay as in Materials and Methods and is displayed on a box plot. **B** and **C**, Heatmaps showing aPL molecular species in platelets. Heatmaps were drawn using the pheatmap R package as described in Materials and Methods to visualize total (**B**) and externalized (**C**) aPL amounts between groups for all species measured using liquid chromatography tandem mass spectrometry in resting or thrombin-activated platelets. Statistical significance was tested with 1-way ANOVA and Tukey post hoc test (*P*<0.05 considered significant). Acute coronary syndrome (ACS; n=24: **A**; n=21: **B** and **C**), coronary artery disease (CAD) but no ACS (n=19), positive risk factors (RFs) with no significant CAD (n=23: **A**; n=22: **B** and **C**), and healthy control (HC; n=24: **A**; n=23: **B** and **C**).

Next, the ability of washed platelets from patient groups to support prothrombinase activity was tested. Thrombin generation was stimulated by platelets from all groups tested, but there were no significant differences seen (Figure 5A). Furthermore, platelet counts were similar between disease groups with all being within the normal range (Table S1). Since aspirin was highly prescribed among patients (Table S1), we next tested whether this drug had an impact on platelet-driven thrombin generation in our assay, using platelets from HC volunteers. No effect of in vitro aspirin was seen, indicating that supplementation was not reducing the ability of platelet membranes to stimulate thrombin generation (Figure S8D). Overall, these data suggest that platelet membrane–driven thrombin generation is not responsible for increased thrombotic risk in ACS.

### Platelets Externalize aPL on Activation, With an Unchanged Molecular Composition Between Disease Groups and HCs

Next, the procoagulant PL composition of platelets was determined using LC/MS/MS. The aPL species detected were as previously found in HC platelets, namely PE P-16:0_20:4, PE P-18:1_20:4, PE 18:0_20:4, PE P-18:0_20:4, PE 18:0_18:1, PS 18:1_18:1, PS 18:0_18:1, and PS 18:0_20:4, as shown previously^27^ (Figure 5B; Figure S10B). Following thrombin activation, platelets from all groups externalized aPL with the most abundant isomers detected in higher amounts on the outside (Figure 5C; Figure S10A).

**Figure 6.**
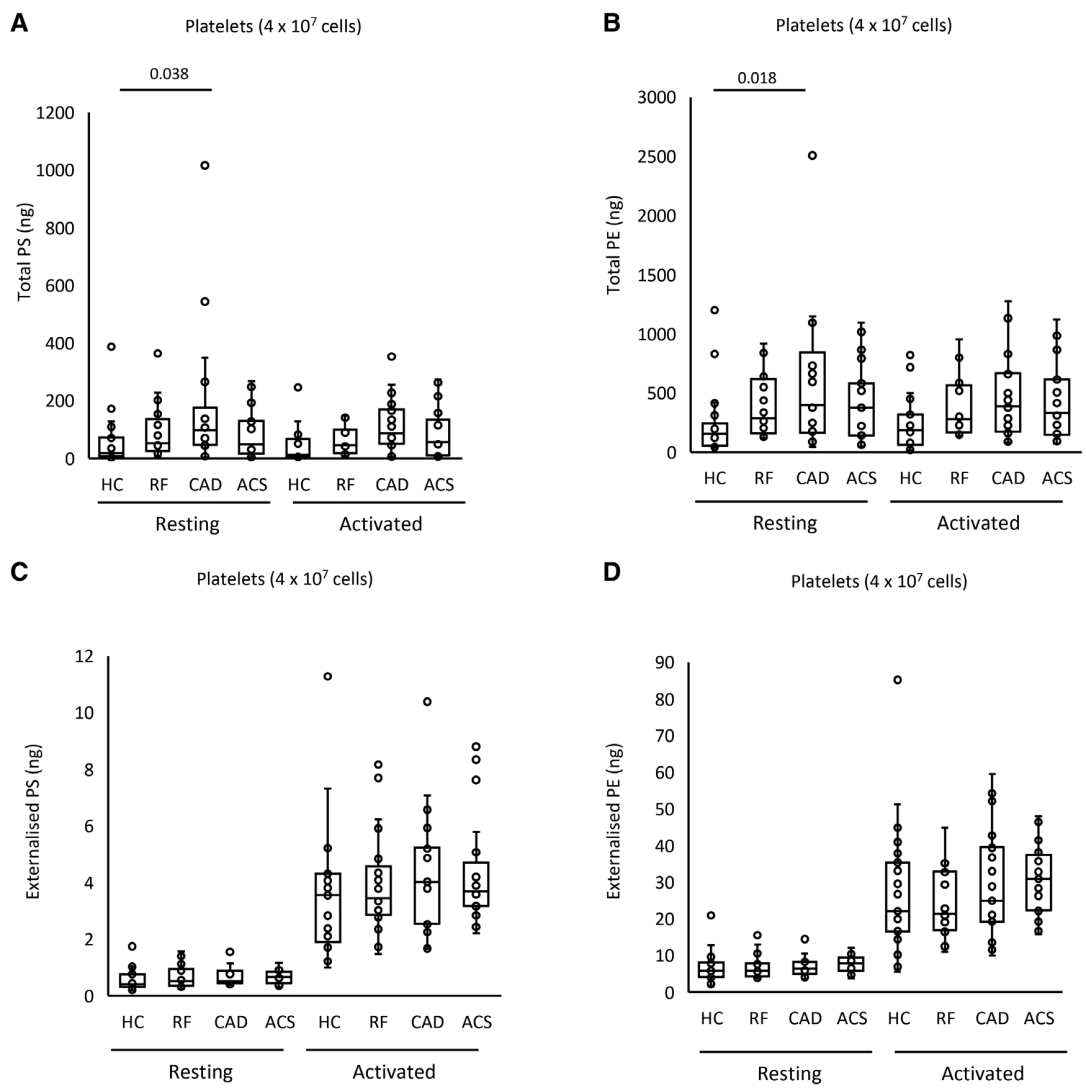
**Minimal differences in aminophospholipid externalization between groups were seen in total and externalized phosphatidylethanolamine (PE) and phosphatidylserine (PS) for platelets.** Lipids were extracted from resting or thrombin-activated platelets as in Materials and Methods. Lipid amounts (ng) were determined using liquid chromatography tandem mass spectrometry. Statistical significance was tested with 1-way ANOVA and Tukey post hoc test (*P*<0.05 considered significant). Acute coronary syndrome (ACS; n=19: **A** and **B**; n=21: **C** and **D**), coronary artery disease (CAD) but no ACS (n=18: **A** and **B**; n=19: **C** and **D**), positive risk factors (RFs) with no significant CAD (n=17: **A** and **B**; n=22: **C** and **D**), and healthy control (HC; n=23).

Total PS and PE levels were somewhat higher in platelets from the CAD group compared with HC in resting and activated conditions (Figure 6A and 6B). Following thrombin activation, externalized PS and PE rose compared with resting platelets, but there were no significant differences between clinical groups and HCs (Figure 6C and 6D). Thus, disease did not result in major changes in either the amounts or molecular species of aPL externalized by platelets. Furthermore, there was no correlation between the amount of thrombin generated and externalized PS and PE amounts on the platelet membrane surface (Figure S8C). In summary, platelets from patients with disease did not have higher PE/PS externalization or elevated thrombin generation capacity, suggesting that changes in platelet aPL do not significantly contribute to the higher thrombotic risk seen in cardiovascular disease.

### Levels of TAT and d-Dimer in Plasma Are Not Altered in Patient Groups

Plasma d-dimer and TAT complexes were measured for all participants. There were no differences between groups in d-dimer levels, and for most participants, TATs were undetectable (Figure S11A and S11B). Additionally, there was no significant correlation between d-dimers and TATs (Figure S11C) or with the amount of thrombin generated on the surface of EVs, platelets, or leukocytes (Figure S11D). Finally, the levels of plasma d-dimer and TAT showed no significant correlations with total or externalized aPL in EV, platelets, or leukocytes (Figures S12 and S13).

## DISCUSSION

Thrombin generation on the plasma membrane surface relies on the presence of procoagulant lipids, primarily native PS and PE, but up to now, it was not known whether thrombin generation or native aPLs are altered in arterial thrombosis. This is important as it may influence the procoagulant status of patients with this condition and lead to further thrombotic complications.^12,13^ Here, thrombin generation and the aPL composition of circulating blood cells and EVs from patients with ASCVD was characterized. Our data suggest that EVs and leukocyte membranes may contribute to increased thrombotic tendency of patients with cardiovascular disease. In contrast, a role for platelets in driving membrane-dependent coagulation was not revealed. This has implications for the clinical management of ACS, which currently does not target leukocyte or EV membrane compartments therapeutically for reducing thrombotic risk.

The assay used in this study to assess thrombin generation is TF independent, and no external PL was added. The means that the assay evaluates the role that the procoagulant membrane PL surface has on prothrombinase activity and is independent of changes in the levels of the patient’s coagulation factors. While there were minimal differences in aPL amounts on the surface of EVs and leukocytes between disease groups and HCs as characterized by LC/MS/MS, the higher count of EV in plasma provided more surface area for the prothrombinase reaction to take place, which in turn led to more thrombin generation. The same is likely for leukocytes, which were found to be higher in patients with ACS compared with RF. Together, these results imply that a simple abundance of circulating procoagulant surfaces is sufficient to alter the amount of thrombin generation in resting states. Targeting EV clearance or shielding the circulation from the aPL membrane may, therefore, present a novel angle to reduce coagulation reactions in this thrombotic condition.

LC/MS/MS to characterize aPL in platelets, leukocytes, and EVs has not been used in patients with arterial thrombosis before this study. Additionally, the external aPL lipidome in leukocytes and EVs has not been previously characterized with LC/MS/MS. The majority of the literature on aPL trafficking and detection utilizes a flow cytometry–based method, which relies on aPL-binding fluorescent probes.^36–38^ The commonest of these is annexin V-FITC (fluorescein isothiocyanate), which can bind to either PS or PE in the presence of calcium.^37,38^ There are a number of limitations to this method, the main one being its nonquantitative nature. The binding of annexin V probes to cells and EVs during flow cytometry exhibits rapidly saturated kinetics, likely as a result of steric hindrance preventing additional aPL from binding to this large protein.^27^ Consequently, while it is feasible to count annexin V^+^ cells and EVs using this method, it is not possible to quantify how much aPL is on the surface, distinguish between PS and PE, or know what molecular species are exposed. This is relevant because the procoagulant activity of aPL can be influenced by fatty acyl composition, with an impaired ability of PE comprising shorter FA chains to support coagulation.^27^ Mass spectrometry with biotin derivatization allows the quantitative analysis of aPL molecular species, distinguishing between external and total aPL amounts.^35^ Our study provides a first characterization of PS and PE species present on circulating membrane surfaces using an assay that distinguishes between total and externalized aPL.^35^ The findings build on the current understanding of aPL externalization, which had previously been reported in patients with ACS using nonquantitative techniques with annexin V/lactadherin binding.^39^

Several studies have described higher numbers of EVs in patients experiencing arterial thrombosis and its risk factors.^40–46^ Patients with hypertension have more circulating EVs, which correlate with their blood pressure readings and are thought to be generated as a consequence of higher shear.^40^ Similarly, diabetes is associated with higher levels of EVs released as a consequence of stimuli such as advanced glycation products and oxidative stress, with a higher procoagulant phenotype in patients with poor glycemic control.^41,42^ Patients with dyslipidemias have upregulated EV levels due to LDL (low-density lipoprotein)-induced membrane blebbing.^43^ Indeed, the development of atherosclerosis may be influenced by EVs, generated by mechanisms described above, which alter the profile of adhesion molecules on endothelial cells and promote monocyte transmigration and vascular inflammation.^47,48^ In addition, the presence of high amounts of EVs within the atherosclerotic plaque may contribute to the thrombotic process that follows plaque rupture.^49^ This may implicate EVs in ACS where elevated numbers were demonstrated in comparison to patients with CAD^44–46^ and shown to positively correlate with high-risk coronary angiographic features.^50^ Previous studies characterizing plasma from patients with ASCVD showed that the highest proportion of EVs in these groups is platelet derived (40%–50%), followed by endothelial derived (20%–25%) and then leukocyte derived (5%–10%).^51^ Neutrophil-derived EVs comprise a relatively low percentage, being around 5% in ACS and CAD, versus <2% in HC.^51,52^ Nevertheless, beyond their proposed use as biomarkers and their elevated amounts, the exact role and regulation of EV in human arterial thrombosis remains largely unstudied.

Our data show no significant changes in platelet-supported thrombin generation or platelet PE/PS externalization. These findings suggest a lack of role for the platelet membrane in raising thrombotic risk in these patients. Notably, the purified factor-based experimental system we used to study membrane involvement does not take into account the many other potential platelet-dependent mechanisms including platelet aggregation, release of platelet-derived EVs, or secondary processes, such as release of bioactive mediators that activate leukocytes (thromboxanes) or stimulate vascular and circulating blood cells to generate EVs. Indeed antiplatelet agents such as GP IIb/IIIa (glycoprotein IIb/IIIa) inhibitors work well in patients with a heavy thrombus burden supporting their central role.^53^

Our study adds significantly to knowledge of EV biology in vascular disease, providing the first characterization of their aPL composition and how this contributes directly to thrombotic tendency. It is generally accepted that annexin V^+^ EVs (containing externalized aPL) are procoagulant. Previous studies have concluded that a higher number of EVs implies more PS exposure in the circulation, which in turn could lead to a more procoagulant phenotype, and our study adds significant new information to support this idea by characterizing the specific molecular species of PS and PE involved.^45,54,55^ Up to now, methods used to study PS^+^ EVs have been limited to flow cytometry–based assays with annexin V. This does not allow accurate quantification of external aPL, individual molecular species, nor does it determine the total aPL content within membranes. LC/MS/MS and biotin derivatization allowed the mapping-specific molecular species and their amounts, while also distinguishing between external and total membrane aPL.^35^

In our study, TATs and d-dimers were similar across all groups. However, published literature is conflicting, with some studies showing elevations and several others not showing changes in coagulation markers in patients with ASCVD.^56–62^ A possible explanation for no increase being seen in this cohort is that many patients were actively taking medications designed to dampen thrombosis via inhibiting platelet activation. However, considering these patients are still at elevated thrombotic risk, the characterization of the role of additional factors such as circulating PL membranes becomes important.

The timing of any intervention targeting leukocytes or EVs in ACS would require careful consideration. In this study, all ACS samples were taken within 48 hours of the onset of the event, and, therefore, the findings reflect the acute phase. This immediate stage is associated with higher thrombotic risk.^63^ As such, it is managed with more intensive antithrombotic treatment in the first few days after ACS such as the addition of a low-molecular-weight heparin to dual antiplatelet therapy.^63^ It is unclear, however, whether the higher procoagulant potential of leukocyte/EV membranes continues in the months after the acute event where persistent activation of the coagulation system has been described in ACS.^64^ To test this, longitudinal studies of patients with ACS in the acute (48 hours), intermediate (6 weeks), and distant (6 months) phases would provide more information on the variation of procoagulant membrane properties over time.

### Study Limitations

While cells from patients with ACS may demonstrate lipidomic and biological differences, it is not possible to determine causality because it is not possible to predict when an ACS event will occur.^65^ Furthermore, the EV cell of origin and TF surface content is unknown. Two separate preps for EV were utilized, for the coagulation assays and EV counting. This was necessary because EVs for the coagulation assay needed to be generated and used on the same day, while EVs for counting could be generated later using frozen plasma. Finally, the cross-sectional design of the study on the clinical cohort and the absence of longitudinal sampling time points (particularly for the acute phase of ACS) may be confounded by interindividual variations, which may reduce statistical power. This, alongside the relatively small sample size, makes further validation and replication in a larger cohort advantageous.

### Conclusions

In this study, the procoagulant membrane in circulating blood cells and EV was determined in patients with ASCVD. Our findings provide the first characterization of aPL in ASCVD circulating membranes and propose that higher membrane procoagulant activity in arterial thrombosis will be driven primarily by EVs and leukocytes. Expanding on these findings and testing whether interference with the procoagulant lipidome alters the observed incidence of arterial thrombosis would move the field closer toward targeting PLs as therapeutic targets for the prevention of thrombosis in high-risk groups.

## ARTICLE INFORMATION

### Sources of Funding

This work was supported by the Wellcome Trust (GW4-CAT fellowship to M.B. Protty, 216278/Z/19/Z) and the British Heart Foundation (program grant to P.W. Collins and V.B. O’Donnell, RG/F/20/110020). V.J. Tyrrell was supported, in part, by the Welsh Government/EU Ser Cymru Programme. A.A. Hajeyah is supported by a grant from the Kuwait University. P.V. Jenkins and A. Sharman were funded by the Government of Kingdom of Saudi Arabia.

### Disclosures

None.

### Supplemental Material

Tables

Figures

Data Set

## Supplementary Material
