# Thrombin generation is associated with extracellular vesicle and leukocyte lipid membranes in atherosclerotic cardiovascular disease

**DOI:** 10.1161/ATVBAHA.124.320902

**Published:** 2024-08-01

**Authors:** Majd B Protty, Victoria J Tyrrell, Keith Allen-Redpath, Shin Soyama, Ali A Hajeyah, Daniela Costa, Anirban Choudhury, Rito Mitra, Amal Sharman, Parveen Yaqoob, P Vince Jenkins, Zaheer Yousef, Peter W Collins, Valerie B O’Donnell

**Affiliations:** 1 Systems Immunity University Institute, Cardiff University, Cardiff, CF14 4XN, UK; 2 Department of Nutritional Sciences, University of Reading, Reading, RG6 6AP, UK; 3 Morriston Cardiac Centre, Swansea Bay University Health Board, Swansea, SA6 6NL, UK; 4 Department of Cardiology, University Hospital of Wales, Cardiff, CF14 4XW, UK

**Keywords:** lipidomics, thrombosis, phospholipids, acute coronary syndrome

## Abstract

**Background:**

Clotting, leading to thrombosis, requires interactions of coagulation factors with the membrane aminophospholipids (aPL) phosphatidylserine (PS) and phosphatidylethanolamine (PE). Atherosclerotic cardiovascular disease (ASCVD) is associated with elevated thrombotic risk which is not fully preventable using current therapies. Currently, the contribution of aPL to thrombotic risk in ASCVD is not known. Here, the aPL composition of circulating membranes in ASCVD of varying severity will be characterized along with the contribution of external-facing aPL to plasma thrombin generation in patient samples.

**Methods:**

Thrombin generation was measured using a purified factor assay on platelet, leukocyte and extracellular vesicles (EV) from patients with ACS (n = 24), stable coronary artery disease (CAD, n = 18), risk factor positive (RF, n = 23) and compared with healthy controls (HC, n = 24). aPL composition of resting/activated platelet and leukocytes, and EV membranes was determined using lipidomics.

**Results:**

External facing aPL were detected on EV, platelets and leukocytes, elevating significantly following cell activation. Thrombin generation was higher on the surface of EV from ACS patients than HC, along with increased circulating EV counts. Thrombin generation correlated significantly with externalized EV PS, plasma EV counts and total EV membrane surface area. In contrast, aPL levels and thrombin generation from leukocytes and platelets were not impacted by disease, although circulating leukocyte counts were higher in patients.

**Conclusions:**

The aPL membrane of EV supports an elevated level of thrombin generation in patient plasma in ASCVD. Leukocytes may also play a role although the platelet membrane did not seem to contribute. Targeting EV formation/clearance and developing strategies to prevent the aPL surface of EV interacting with coagulation factors represents a novel anti-thrombotic target in ASCVD.

## Graphical abstract

**Figure:**
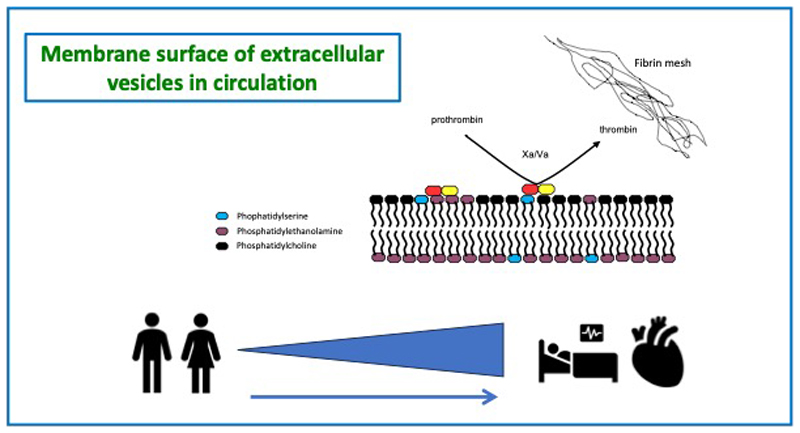

## Non-Standard Abbreviations

ASCVD: Atherosclerotic cardiovascular disease
EV: Extracellular vesicles
aPL: Aminophospholipids
HC: Healthy controls
RF: Risk factor positive
ACS: Acute coronary syndrome
CAD: Coronary artery disease
TF: Tissue factor
PS: Phosphatidylserine
PE: Phosphatidylethanolamine
PL: Phospholipid
LC/MS/MS: Liquid chromatography tandem mass spectrometry
TAT: Thrombin-anti thrombin complex

## Introduction

Atherosclerotic cardiovascular disease (ASCVD) refers to a spectrum of conditions ranging from acute coronary syndrome (ACS), stable coronary artery disease (CAD), and patients with risk factors that have not yet developed obstructive coronary artery stenosis (RF). Worldwide, ASCVD is responsible for the death of more than 9 million people annually, with an estimated prevalence of approximately 200 million^1,2^. The most severe form of ASCVD is ACS which is triggered by atherosclerotic plaque rupture leading to activation and recruitment of platelets and leukocytes along with upregulation of tissue factor (TF) expression^3–10^. This activates coagulation, forming an occlusive arterial thrombus driving ischemia and infarction^11^. Despite standard treatment with anti-platelet agents, the rates of subsequent strokes, myocardial infarction and cardiovascular death exceed 10% in the first-year post ACS diagnosis^12,13^. This indicates that there are likely to be other modifiable factors involved in ASCVD beyond platelet activity^12–14^.

Thrombin generation requires a procoagulant phospholipid (PL) membrane to allow the assembly of the “prothrombinase” (Factor Xa/Va) complex and other Gla-domain containing coagulation factors^15^. This can be provided by the external surface of activated platelets, leukocytes and extracellular vesicles (EV)^16,17^. Resting platelet and leukocyte membranes are comprised primarily of phosphatidylcholine (PC) on the external side, with the aminophospholipids (aPL) phosphatidylethanolamine (PE) and phosphatidylserine (PS) being internally facing^18^. The binding of coagulation factors requires a specific phospholipid (PL) composition which includes the electronegative headgroup of PS, supported by PE, and is dependent on the presence of calcium ions^19,20^. Native PC does not facilitate the binding of coagulation factors and so resting platelets or leukocytes provide minimal support for coagulation reactions. During inflammation or acute trauma/bleeding challenge, platelets and leukocytes become activated, leading to a calcium-dependent translocation of PS/PE to the outside of the cell, mediated by scramblase^21^. Inflammation also leads to the release of aPL-rich EV. Together, this exposure of aPL to the circulation leads to interactions with coagulation factors via calcium which are essential for effective coagulation in vivo^22–24^.

Whether the enhanced thrombotic risk in ASCVD is related to altered levels of aPL-mediated thrombin generation on the surface of blood cells is currently unknown. Previous studies have focused on differences in coagulation factor amounts and activity, but there have been no studies focusing on the contribution of the PL membrane to thrombin generation independent of tissue factor or plasma content^25^. To determine this, we characterized the aPL membrane composition in platelets, leukocytes and EV in patients with ACS, CAD and RF. We assessed the ability of these membranes to support coagulation in vitro compared with healthy controls (HC) using a thrombin generation assay that employs purified coagulation factors independent of tissue factor^26–29^. This approach allowed the contribution of the pro-coagulant membrane to coagulation to be determined specifically.

## TOP

### Methods

#### Study Participants for clinical cohort (blood samples)

*Clinical cohort:* Participants were recruited from Cardiff University and Cardiff and Vale University Health Boards. Ethical approval was from Health and Care Research Wales (HCRW, IRAS 243701; REC reference 18/YH/0502). Age and sex-matched individuals were recruited into one of the following four groups: *(i) Acute Coronary Syndrome (ACS)*: Participants were identified on in-patient cardiology wards using diagnostic tests (ischemic ECG changes, raised troponin level above normal laboratory defined range) and clinical assessment by the cardiology team. All were recruited within 48 hours of the index event prior to any revascularization/angioplasty. *(ii) Significant coronary artery disease (CAD):* Patients attending for an elective coronary angiogram to assess for symptoms of stable angina in the absence of a history of ACS. Coronary angiography demonstrated lesions requiring revascularization on anatomical/physiological criteria as defined by guidelines from the European society for cardiology (ESC, 2018)^30^. *(iii) Risk-factor controls with no significant CAD (RF):* This group includes patients attending for a diagnostic coronary angiogram with risk factors for ischemic heart disease (a clinical diagnosis of hypertension requiring therapy, diabetes types 1 or 2, hypercholesterolemia [total cholesterol > 6 mmol/L], smoking, chronic kidney disease stage 3 or more, or combination thereof) but whose coronary angiogram demonstrates no significant coronary artery disease, defined as not requiring revascularization on anatomical/physiological criteria as per the ESC 2018 guidelines^30^. *(iv) Healthy controls (HC):* Participants had no significant history for ischemic heart disease or its risk factors, were never-smokers, and were not on anti-platelet agents, anti-coagulants or statins. They were identified from the workplace or were volunteers from partner studies such as ‘HealthWise Wales’^31^. Clinical characteristics are in Table S1. Inclusion criteria were aged 18 years or over, acute coronary syndrome in ACS group, and no history of ACS in the others. Exclusion criteria were: diagnosis of infective endocarditis or atrial fibrillation, or inability to consent to study. Overall, 90 participants were recruited: HC, n = 24, RF, n = 23, CAD, n = 19, ACS, n = 24. Blood samples were collected by peripheral venepuncture as outlined below, by one individual and all samples were transferred to the laboratory within 10 min. The study design is summarized in Figure S1. Blood cell counts were not available for 2 CAD patients. In our human study, numbers of males and females in patient groups were not significantly different, when compared to healthy controls, using Fisher’s exact test (Table S1). Data from males and females were combined and sex differences were not determined in the study due to group sizes being too small. Primary analyses were pre-planned, not post-hoc and p values are specified on figures where < 0.05. Power calculations were not performed due to the lack of relevant primary data with which to perform them.

#### Platelet isolation

Whole blood was taken from using a 21G butterfly needle into a 50 ml syringe containing acidified citrate dextrose (ACD; 85 mM trisodium citrate, 65 mM citric acid, 100 mM glucose) at a ratio of 8.1 parts whole blood to 1.9 parts ACD, as described previously^26^, and centrifuged at 250 *g* for 10 min at 20 °C. The platelet-rich plasma was collected and centrifuged at 1000 *g* for 8 min at 20 °C. Platelet poor plasma was removed and retained for extracellular vesicle isolation. The platelet pellet was resuspended in Tyrode’s buffer (134 mm NaCl, 12 mm NaHCO_3_, 2.9 mm KCl, 0.34 mm Na_2_HPO_4_, 1.0 mm MgCl_2_, 10 mm Hepes, 5 mm glucose, pH 7.4) containing ACD (9:1, v/v). The platelets were washed by centrifuging at 1000 *g* for 8 min at 20 °C then resuspended in Tyrode’s buffer at 2 × 10^8^·ml^−1^. Platelets were activated at 37 °C in the presence of 1 mM CaCl_2_, 0.2 unit·ml^−1^ thrombin (Sigma Aldrich).

#### Leukocyte isolation

Leukocytes were isolated from 20 ml citrate-anticoagulated whole blood as described previously^26^. Briefly, 20 ml of blood was drawn using a 21G butterfly needle into a 50 ml syringe containing 4 ml of 2 % citrate and 4 ml of Hetasep (Stem Cell Technologies) and allowed to sediment for 45 minutes. The upper plasma layer was recovered and centrifuged at 250 g for 10 min at 4 °C. The pellet was resuspended in ice-cold 0.4 % trisodium citrate/PBS and centrifuged at 250 g for 5 min at 4 °C. Erythrocytes were removed by hypotonic lysis (0.2 % hypotonic saline before being neutralized with a PBS wash. Leukocytes were resuspended in Krebs buffer (100 mM NaCl, 48 mM HEPES, 5 mM KCl, 1 mM sodium dihydrogen orthophosphate dihydrate and 2 mM glucose) at 4 x 10^6^/ml. For activation, 4 x 10^6^ leukocytes were incubated at 37 °C with 10 μM A23187 and 1 mM CaCl_2_, for 30 min, prior to lipid extraction.

#### Extracellular vesicle (EV) isolation for clinical assay

Methods were adapted from recent literature and guidelines^32,33^. Platelet poor plasma generated as above was centrifuged at 1000 g for 10 min at 20 °C to generate platelet-free plasma (PFP). 1 ml PFP was snap frozen on dry ice and stored at -80 °C for quantification at a later date as below. For each donor’s plasma, 6 x 1 ml PFP aliquots were centrifuged at 16,000 g for 30 min at 20 °C. 750 μl was removed from each aliquot, and 750 μl of modified Tyrode’s buffer was added to the pellet, which was gently resuspended using a pipette. Following a second centrifugation as above, 950 μl was removed. 50 μl modified Tyrode’s buffer was added to the pellet to gently resuspend and recover the EV-rich fraction. EV fractions were pooled to generate one isolate per donor. Of this, 250 μl was used for lipidomics, and 3 x 20 μl for prothrombinase assays.

#### Extracellular vesicle (EV) quantification for clinical assay

EV quantification was performed by thawing one aliquot of PFP per patient, of which 500 μL was passed through size-exclusion chromatography iZON qEV columns (Izon Science Ltd, UK) to recover particles and vesicles between 70 nm to 1000 nm in diameter as outlined in the manufacturers information. The eluting EV-rich fractions were collected and analyzed using nanoparticle tracking on a NanoSight 300 (Malvern, UK) equipped with a sensitive sCMOS camera and a 488 nm blue laser, to generate an EV count and size distribution for all participants. For two samples, a measure of count and size could not be obtained due to turbidity.

#### ELISA

Commercially available ELISA kits were purchased (Abcam, UK) for the measurement of apolipoprotein B (ab190806), D-Dimer (ab260076) and human thrombin anti-thrombin complexes (ab279724) on frozen plasma samples. These were quantified on a plate reader for absorbance. For all available information on the kits and antibodies used, please see the following links: https://www.abcam.com/ps/products/190/ab190806/documents/Human-Apolipoprotein-B-elisa-kit-protocol-book-v3-ab190806%20(website).pdf https://www.abcam.com/ps/products/260/ab260076/documents/Human-D-Dimer-ELISA-Kit-protocol-book-v3a-ab260076%20(website).pdf

#### Testing for lipoprotein contamination EV isolated by SEC

Twelve male and female subjects (females=10, males=2) (aged 22-37y) were recruited from the University of Reading. All subjects gave informed consent. Ethical approval for the study was obtained from the School of Chemistry, Food and Pharmacy Ethics Committee at the University of Reading (Study number 17/17) and conducted according to the guidelines laid down in the Declaration of Helsinki. The inclusion criteria comprised the following: Age range 18-65 y, nonsmoker, haemoglobin ≥115 g/l for women and 130 g/l for men, total cholesterol < 5 mmol/l, TAG 0.4-1.5 mmol/l, no disclosed history of drug or alcohol abuse, no illness or disease requiring medication (excluding HRT, oral contraceptive, and thyroxine replacement therapy). Participants were excluded if unwell or on any prescribed medication and were asked to avoid exercise, alcohol and fatty meals the day before the experimental visit, consuming a low-fat meal the evening before the visit. Participants attended the Hugh Sinclair Unit of Human Nutrition following a 12 h overnight fast and a baseline fasting blood sample was taken before consuming a high fat test meal consisting of two all-butter croissants containing 24 g fat, 10.6 g protein and 48 g carbohydrate. A second blood sample was collected 4 h later, corresponding to the point at which postprandial lipaemia reaches a peak. Venous blood samples were drawn into citrated tubes, inverted 4 times, and processed immediately. Samples were centrifuged at 1500 x g at room temperature for 15 min to remove larger cells and cellular debris. Further centrifugation at 13000 x g for 2 min at room temperature produced PFP, which was aliquoted and stored at -80°C for further analysis.

(i) *Isolation of EVs using SEC*. PFP (0.5 ml) was thawed at room temperature on a sample roller and loaded onto a qEV original column (Izon, Oxford, UK), which had been pre-flushed with 30 ml phosphate-buffered saline (PBS). A further 5 ml PBS was passed through the column to elute EVs based on their size and 0.5 ml fractions were collected.
(ii) *Lipoprotein isolation*. Density gradient centrifugation was performed on baseline and postprandial PFP to isolate chylomicrons, VLDL-1 and VLDL-2, and the fractions were stored at -20°C in the presence of apo B48 preservative (5% v/v) for later analysis, as described in^34^.
(iii) *Assessment of contamination of EV fractions with apoB48 and apoB100*. To determine whether EV fractions prepared from PFP by SEC were contaminated with chylomicrons or VLDL, EV fractions prepared from both baseline and postprandial samples of PFP were subjected to ELISA for Apo B48 (CM) and Apo B100 (VLDL). The presence of CM was evaluated using the Human Apolipoprotein B48 ELISA kit purchased from ElabScience (Houston, USA) and the presence of VLDL was investigated using the human apolipoprotein B100 ELISA kit. (SIGMA-Aldrich, Saint Louis, USA). The ELISA tests were performed according to the manufacturer’s instructions and read on a plate reader (Spark, Tecan, UK).
(iv) *Assessment of the potential co-isolation of lipoproteins prepared by density gradient centrifugation with EVs*. CM, VLDL-1 and VLDL-2 fractions prepared by density gradient centrifugation (as described above) were subjected to SEC to determine whether there was co-elution of lipoproteins and EVs. Fractions were analysed using nanoparticle tracking analysis.

#### Prothrombinase assay

Resting platelets (4 x 10^6^), resting leukocytes (8 x 10^4^) and plasma EV isolates (20 μL) were added in triplicate to a 96-well half-area flat bottom clear plate (Greiner, Austria). Next, a mix of recombinant factor Xa (Fxa) (50 nM, Enzyme Research Laboratories, UK), factor Va (Fva) (15 nM, Haematologic/Cambridge Bioscience, UK), factor II (FII) (1 μM, Enzyme Research Laboratories, UK) and CaCl_2_ (5 mM) in prothrombinase buffer (20 mM Tris, 150 mM NaCl, 0.05% BSA w/v) was added to the wells and the reaction allowed to proceed for 5 min at 21 °C, before being quenched with an excess of EDTA (7 mM final concentration). Thrombin (FIIa) activity was measured on a plate reader using a chromogenic substrate S-2238 (Enzyme Research Laboratories, UK). A graphical depiction of the assay is shown in Figure S2.

#### Lipid biotinylation, extraction and analysis for aPL

To determine the amounts of PE and PS on the external leaflet of cell membranes, total and external aPL were measured as described previously^35^. Briefly, 0.2 mL of platelets (4 x 10^7^), leukocytes (8 x 10^5^) or EV isolated as described above were incubated with 20 μL of 20mM NHS-biotin for 10 min at 21 °C to label total aPL. In the case of externalized aPL, samples were incubated with 86 μL of 11mM EZ-Link sulfo-NHS-biotin for 10 min at 21 °C followed by 72 μl of 250 mM of L-lysine for 10 min at 21 °C. The final volumes were made up to 0.4 mL with phosphate-buffered saline (PBS). Lipids were extracted by adding samples to 1.5 mL chloroform:methanol (1:2) containing 10 ng internal standards (biotinylated 1,2-dimyristoyl-PE and -PS, generated as in Thomas et al^35^) to give a solvent:sample ratio of 3.75:1, as described previously^35^. Following vortexing and centrifugation (400 g, 5 mins), lipids were recovered in the lower chloroform layer, which was dried under vacuum. Samples were analyzed for aPL using LC/MS/MS. For this, samples were separated on an Ascentis C-18 (5 μm 150 mm × 2.1 mm) column (Sigma Aldrich, USA) with an isocratic gradient (methanol, 0.2 % w/v NH_4_CH_3_CO_2_) at a flow rate of 400 μL/min. Products were analyzed in MRM mode on a Q-Trap 4000 instrument (Applied Biosystems, UK) by monitoring precursor-to-product ion transitions in negative ion mode (Table S2). The peak area for the analytes was integrated and normalized to the internal standards. Limit of quantitation (LOQ) is defined as signal:noise of 5:1 with at least 6 data points across a peak. For quantification, standard curves were generated using biotinylated PS and PE^35^. Chromatograms of biotinylated PS and PE species as detected in representative participant samples can be seen in Figures S3,4, respectively. For 5 patient extracts processed on the same day, internal standard could not be detected during subsequent LC/MS/MS and they were excluded. In a further 8 samples, while conducing LC/MSMS analysis of the full cohort samples, we identified that a batch of NB used for this subset of samples had been inactive. SNB used for those samples was active so externalized but not total PE/PS levels were determined for these samples. Full data on all sample numbers are included in legends for clarity.

#### Statistical analysis

Statistical significance was determined using one way ANOVA with Tukey Post Hoc test (astatsa.com). For platelets and leukocytes, resting or activated samples were compared separately. Correlation analysis utilized Pearson’s correlation for linear dependence between variables. The cut-off value chosen for test significance was a p-value <0.05. Box plots were drawn in Excel (Microsoft, USA) with edges indicating the interquartile range (IQR), the line inside the box indicating the median, and whiskers indicating 1.5 times the IQR. For generation of heatmaps, samples were averaged within their groups, and a log10 was applied to lipid amounts (ng) normalized to cell count or tissue weight (mg) for each lipid to allow row-wise and column-wise comparison. Next, lipid measurements were plotted as intensity values using the ‘pheatmap’ package in the R coding environment (v3.6.2, open source) with lipid hierarchical clustering. Intensity levels were represented by a colour gradient ranging from blue (very low levels or absent) to red (high levels) with variations in between. Graphical illustrations of assays, designs and pathways were carried out using the online platforms draw.io and biorender.com (premium subscription). For lipidomics data statistical analysis, missing values where lipids were below LOQ were replaced with 50% of the assay LOQ value. If any lipid was missing more than 50 % in a sample set, it was not analysed. Where values were imputed, they are shown in red in the Supplementary Dataset.

## Results

### EV-containing plasma from ACS patients supports elevated thrombin generation, driven by higher EV counts in disease

EV fractions isolated using either centrifugation or SEC were found to be free of lipoproteins as measured using the ab190806 human apolipoprotein B ELISA kit (Abcam, UK) (Figure S5 A), or by monitoring lipoprotein elution from SEC (Figure S5 B-F). Here, When PFP was subjected to SEC, EVs eluted in Fractions 7-9. However CM (apo B48) and VLDLs (apo B100) in PFP only began to elute from Fraction 11 onwards (Figure S5 B,C), demonstrating that lipoproteins elute later than EVs, with little cross-contamination. This is consistent with lipoproteins having a smaller diameter than EVs. Lipoproteins isolated first by density gradient centrifugation and then subjected to SEC did not elute in EV enriched fractions (Fractions 7-9) and began to elute in the later fractions (Figure S5 D-F). Specifically, CM isolated from fasted plasma eluted after fraction 12 and those isolated from postprandial plasma eluted after fraction 10, indicating that the postprandial CM population was larger in diameter, but still distinct from the EV population. The VLDL-1 population eluted after Fraction 14 and the VLDL-2 population eluted after fraction 16, indicating the progressively smaller diameters of these lipoproteins.

**Figure 1:**
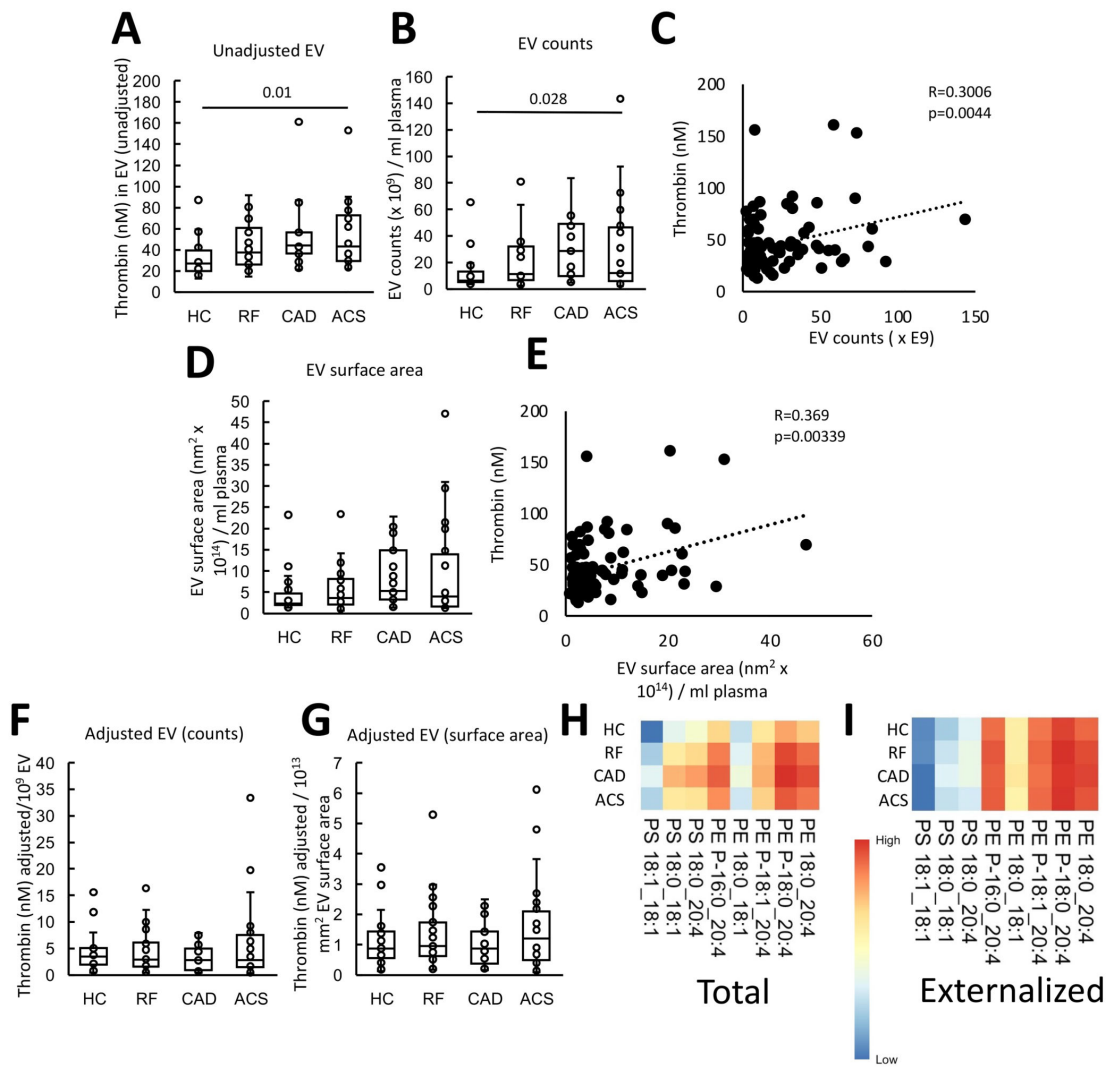
Plasma EV support elevated thrombin generation in ASCVD plasma. *Panel A. EV from patients support higher levels of thrombin generation*. The ability of EV membranes to support thrombin generation was assessed using prothrombinase assay as described in Methods. *Panel B. EV counts are significantly higher in patients*. To quantify EV, platelet-free plasma (0.5 mL) was processed using size-exclusion chromatography (iZON qEV columns) and nanoparticle tracking analysis (Nanosight 300). *Panel C. Thrombin generation positively correlates with EV counts*. Thrombin generation and EV counts were correlated using Pearson’s correlation. *Panel D. EV surface area is increased in vascular disease*. EV surface area was calculated as described in Methods and plotted as box plots. *Panel E. Thrombin generation positively correlates with EV surface area*. Thrombin generation and EV surface area were correlated using Pearson’s correlation. *Panel F,G. Thrombin generation was not changed between groups if EV were normalized by counts or surface area*. Thrombin generation on the surface of EV was adjusted by EV counts (per 1x10^9^ EV) (Panel F) and surface area (Panel G) and plotted as box plots. *Panels H,I. Heatmaps showing aPL molecular species in EV*. Heatmaps were drawn using the pheatmap R package as described in methods to visualize aPL amounts between groups for all the measured species, analyzed using LC/MS/MS. Statistical significance was tested with one way ANOVA and Tukey Post Hoc test (p <0.05 considered significant). ACS: acute coronary syndrome (n=24 Panel A,B,D,F,G, n=21 panels H,I), CAD: coronary artery disease but no ACS (n=19 panel A,B,D,F,G), RF: Risk factors with no significant coronary artery disease (n=23 panel A,B,D,F,G, n=22 panels H,I), HC: Healthy control (n=24, panels A, n=22 panels B,D,F,G, n=23 panels H,I).

In an in vitro system using purified Fxa, Fva and FII, EV-containing plasma from patients with ACS stimulated significantly higher thrombin generation than those from HC, while there was a higher trend for RF and CAD (Figure 1 A). EV were isolated from a fixed volume (6 mL) of plasma, thus, particles were quantified using nanoparticle tracking analysis. There was a trend for higher EV counts in plasma from all patient groups compared with HC, which was significantly higher for CAD (Figure 1 B). Furthermore, there was a significant weak positive correlation between thrombin generation and EV counts (Figure 1 C). The mean EV vesicle diameter was significantly smaller in RF and CAD, and slightly smaller in ACS, compared with HC (Figure S6). As coagulation takes place on the surface of vesicles, we next calculated the EV total surface area (vesicle area as a sphere calculated as 4πr^2^ x EV counts) in plasma. A trend was seen for higher EV total surface areas in patient groups although they showed greater overall variability (Figure 1 D). Thrombin generation correlated significantly with EV total surface area indicating a direct relationship between EV membrane and coagulation (Figure 1 E). Overall, this indicates that EV from ASCVD patient groups (RF, CAD and ACS) tend to be smaller than HC, but due to higher counts, the overall EV surface area is elevated in disease groups and will directly contribute to elevated thrombin generation observed. Confirming this idea, we normalized thrombin generation to either EV counts or surface area and found that this abolished the differences between groups (Figure 1 E-F).

### Characterisation of aPL molecular species in EV shows levels tend to be higher in disease groups

The aPL composition of EV has not been described before, hence total and external PS and PE species were next characterized using LC/MS/MS. In this assay, external facing aPL are derivatized using the cell impermeable biotinylation reagent, sulfo-NHS-biotin. Total aPL is instead derivatized using the cell-permeable form, NHS-biotin^35^. Derivatized aPL are then detected using LC/MS/MS, based on a mass shift of +226 a.m.u. from the native lipid. This method was previously used to determine the molecular species and amounts of PS and PE on the surface of platelets from healthy donors^27^. The most abundant species detected were: PE P-16:0_20:4, PE 18:0_20:4, PE P-18:0_20:4, PS 18:0_18:1 and PS 18:0_20:4 (Figure 1 G, Figure S7 A), as previously shown in healthy platelets^27^. Externalized PS and PE comprised the same molecular species, with the most abundant isomers being detected in higher amounts on the outside (Figure 1 H, Figure S7 B). This indicates an absence of selectivity for any particular aPL isomers to be present on the outer side of EV.

**Figure 2:**
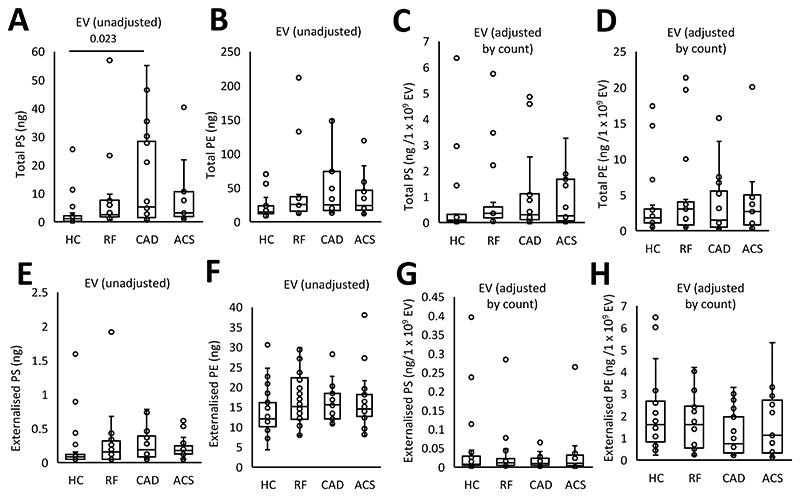
Levels of externalized PE and PS on EV reflect higher EV counts in patients. *Panels A,B. Grouping by headgroup, the amounts of total PS and total PE for each of the clinical groups were plotted to examine for differences between groups. Panel C,D. Total aPL amounts adjusted by EV count. Panel E,F. Grouping by headgroup for externalized PS and PE lipids. Panel G,H. Externalized aPL amounts adjusted by EV count*. Lipids were extracted from EV-rich plasma fractions as in Materials and Methods. Lipids amounts (ng) were calculated by LC/MS/MS. Statistical significance was tested with one way ANOVA and Tukey Post Hoc test (p <0.05 considered significant). ACS: acute coronary syndrome (n = 19 panels A-D, n=21 panels E-H), CAD: coronary artery disease but no ACS (n = 18 panels A-D, n=19 panels E-H), RF: Risk factors with no significant coronary artery disease (n = 17 panels A,B,C,D, n=22 E,F,G,H), HC: Healthy control (n=23 panels A,B,E,F. n=22 panels C,D,G,H).

EV from plasma of CAD patients contained higher levels of total PS and total PE, with a trend for higher levels in all patient groups, compared to HC (Figure 2 A,B), however, once data were normalized to EV counts, this disappeared indicating that it was due to higher EV being present in plasma from ACS and CAD patients (Figure 2 C,D). External PS amounts significantly correlated with thrombin generation on EVs in line with a functional involvement in driving coagulation (Figure S8 A). Externalized PS showed a non-significant trend to be higher in all patient groups versus HC, although externalized PE was similar (Figure 2 E,F). Once adjusted by EV count, externalized aPL trends disappeared (Figure 2 G,H). These data suggest that EV drive coagulation via their external PS which may be somewhat elevated in ASCVD groups compared with HC.

### Thrombin generation in leukocytes from ACS patients is slightly higher than HC

**Figure 3:**
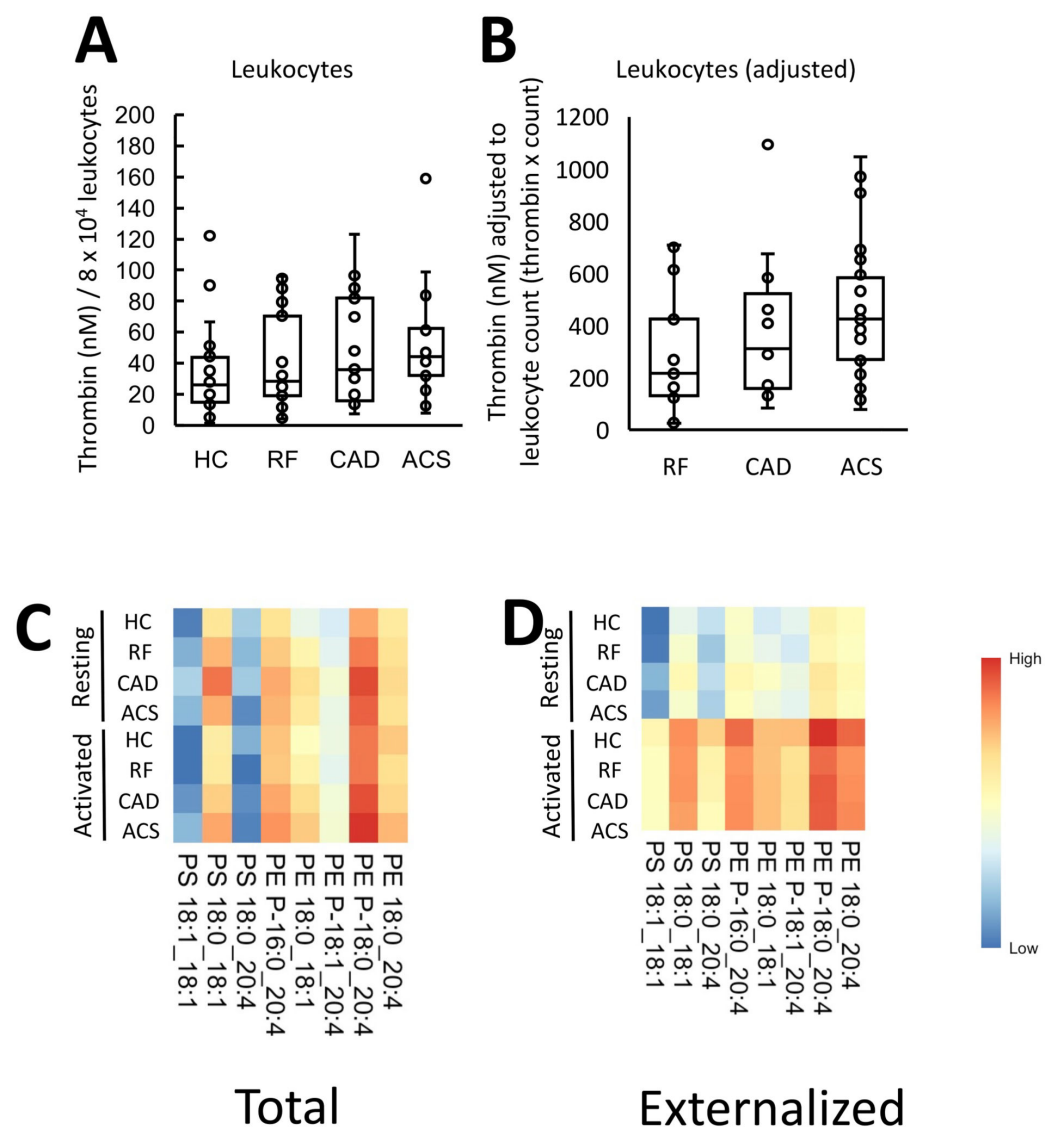
Leukocytes from ACS patients generated more thrombin than HC, and this may be further increased by higher *in-vivo* leukocyte counts. *Panel A. Leukocytes from ACS patients support higher levels of thrombin generation than HC*. The ability of leukocyte membranes to support thrombin generation was quantified using prothrombinase assay as described in Methods and displayed on a box plot. *Panel B. Adjusting by total leukocyte count demonstrates an upward trend in thrombin generation between RF, CAD and ACS samples*. Thrombin generation was adjusted by total leukocyte count to account for differences between groups. *Panels C,D. Heatmaps show aPL molecular species in leukocytes, with increased externalization upon activation*. Lipids were extracted from resting or ionophore activated leukocytes and quantified using LC/MS/MS as described in Methods. The log10 lipid amounts (ng) were plotted on a heatmap using the pheatmap R package as described in Methods to show total (Panel C) and externalized (Panel D) aPL molecular species. Statistical significance was tested with one way ANOVA and Tukey Post Hoc test (p <0.05 considered significant). ACS: acute coronary syndrome (n=24, n=21 panels C,D), CAD: coronary artery disease but no ACS (n=19 panel A, n=17 panel B), RF: Risk factors with no significant coronary artery disease (n=23, n=22, panels C,D), HC: Healthy control (n=24 panel A, n=23 panels C,D).

Next, we characterized the pro-coagulant membrane on circulating white cells from patient groups, also comparing their ability to support thrombin generation with PE/PS content. Resting leukocytes (8 x 10^4^) from patients showed a trend to support higher thrombin generation than HC, in particular for ACS patients (Figure 3 A). In ASCVD groups, there were significantly higher circulating leukocyte counts as measured by the hospital clinical laboratory, with worsening disease, although all cell counts were within normal range (Table S1). To test the potential impact of cell count *in vivo*, thrombin generation was normalized by circulating leukocyte count, since a higher count in disease could further impact thrombin generation. This further demonstrated an upward trend with higher amounts of thrombin predicted to be generated in ACS compared with RF (Figure 3 B). Leukocyte counts had not been obtained for HC, as volunteers were outside the hospital system, so direct comparison with this group was not possible.

### Leukocytes externalize aPL on activation, although molecular composition is unchanged in coronary artery disease

**Figure 4:**
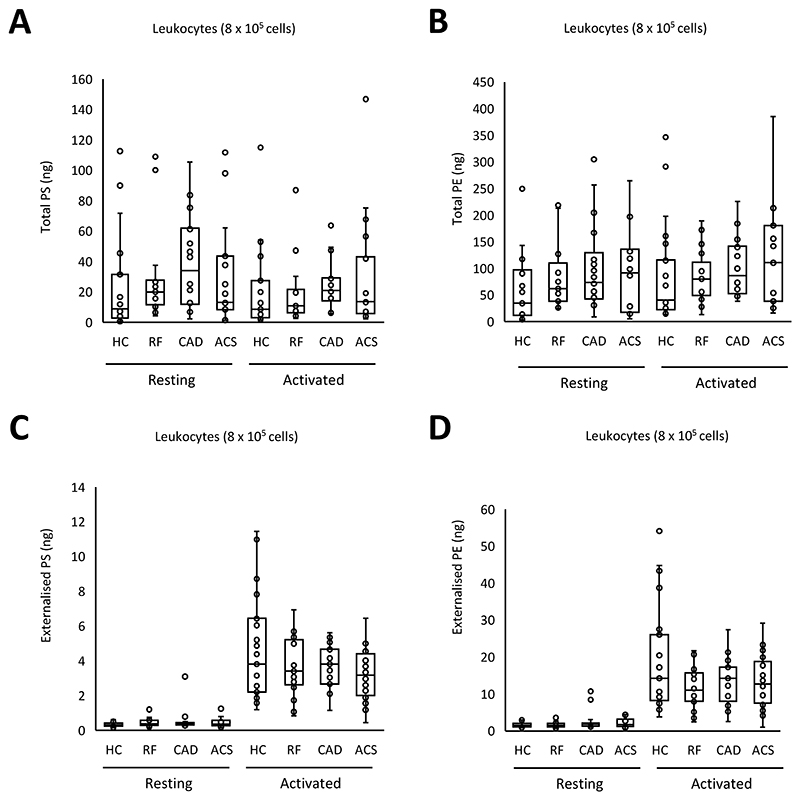
Leukocytes from ACS patients contain and externalize similar amounts of PE/PS, when expressed on a per cell basis. *Panels A-D*. Lipids were extracted from resting or ionophore-activated leukocytes as described in Methods. Lipids amounts (ng) were determined using LC/MS/MS. Statistical significance was tested with one way ANOVA and Tukey Post Hoc test (p <0.05 considered significant). ACS: acute coronary syndrome (n = 19 panels A,B, n=21 panels C,D), CAD: coronary artery disease but no ACS (n = 18 panels A,B, n=19 panels C,D), RF: Risk factors with no significant coronary artery disease (n = 17 panels A,B, n=22 panels C,D), HC: Healthy control (n=23).

The aPL composition of leukocyte membranes is currently unknown. To characterize this, and determine the impact of ASCVD, total and external molecular species of aPL were quantified in leukocytes, both before after calcium ionophore activation. Activation reveals the potential of the cells, when fully activated, to externalize aPL, while resting cells are more comparable with circulating cells. Three PS and five PE species were detected, with all PE containing 20:4, and of a similar composition to EV (Figure 3 C,D, Figure S9 A). Total PE and PS was similar for all groups, and this was not impacted by activation (Figure 4 A,B Figure S9 B). In contrast, following ionophore activation, leukocytes from all groups externalized aPL (Figure 4 C,D). The most abundant aPL isomers were detected in higher amounts on the outside indicating a lack of selectivity for any specific aPL isomer to be externalized in leukocytes (Figure 4 C,D, Figure S9 A). Externalized aPL species or amounts showed only small differences between patient groups, and there was no significant correlation between the amount of thrombin generated on the surface of resting leukocytes and externalized PS and PE amounts (Figure 4 C,D, Figure S8 B). These data indicate that the elevated thrombin generation on the surface of leukocytes in ACS compared with HC is unlikely to be related to levels of externalized aPL.

### Thrombin generation on the surface of resting platelets was similar for all groups

**Figure 5:**
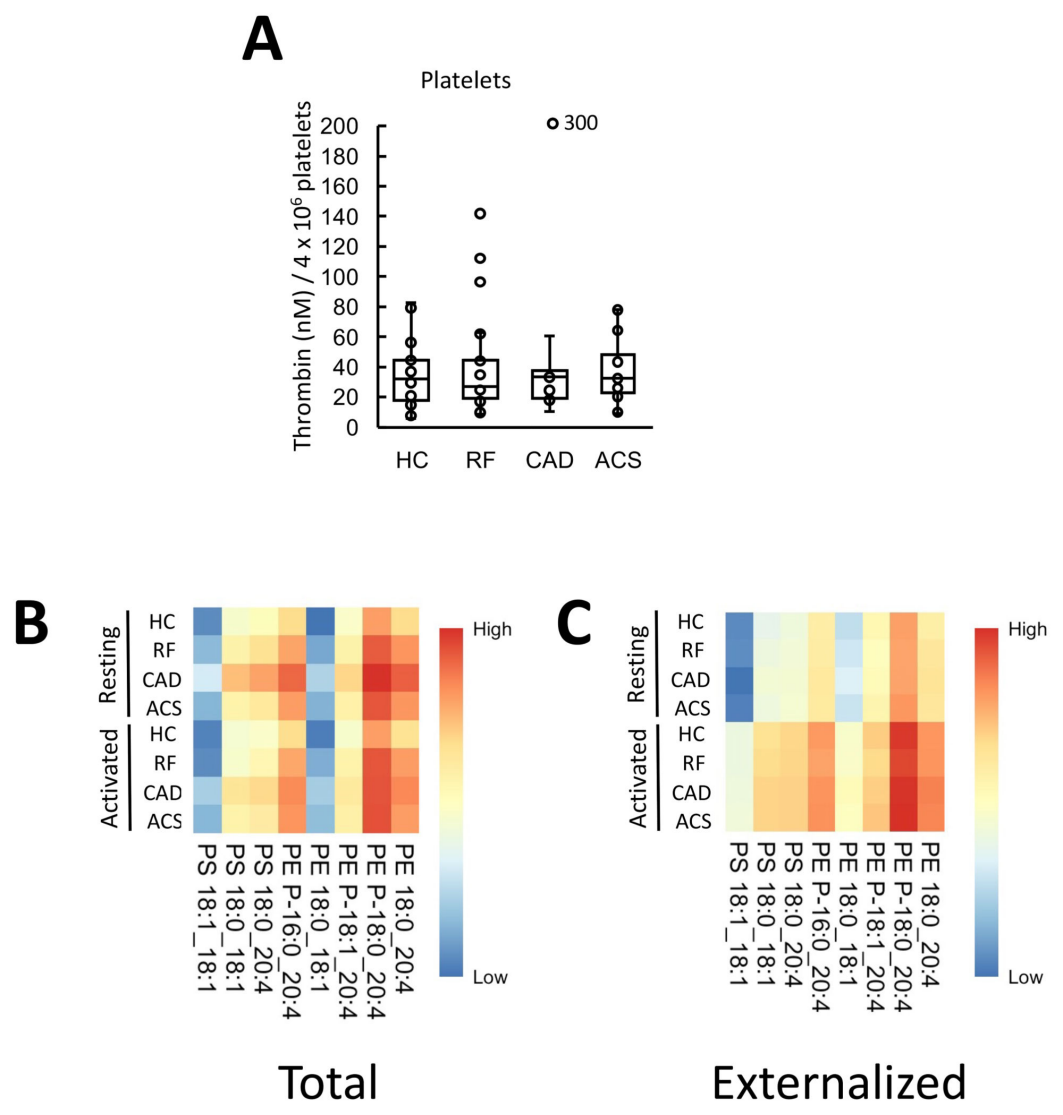
Thrombin generation on the surface of ASCVD platelets was unchanged from HC, and there were minimal differences in aPL externalization between groups. *Panel A. Thrombin generation on the platelet surface was similar between clinical groups*. The ability of platelet membranes to support thrombin generation was assessed using the prothrombinase assay as in Methods and is displayed on a box plot. *Panels B,C. Heatmaps showing aPL molecular species in platelets*. Heatmaps were drawn using the pheatmap R package as described in Methods to visualize total (Panel B) and externalized (Panel C) aPL amounts between groups for all species measured using LC/MS/MS in resting or thrombin-activated platelets. Statistical significance was tested with one way ANOVA and Tukey Post Hoc test (p <0.05 considered significant). ACS: acute coronary syndrome (n=24 panel A, n=21 panels B,C), CAD: coronary artery disease but no ACS (n=19), RF: Risk factors with no significant coronary artery disease (n=23 panel A, n=22 panels B,C), HC: Healthy control (n=24, panel A, n=23 panels B,C).

Next, the ability of washed platelets from patient groups to support prothrombinase activity was tested. Thrombin generation was stimulated by platelets from all groups tested, but there were no significant differences seen (Figure 5 A). Furthermore, platelet counts were similar between disease groups with all being within the normal range (Table S1). Since aspirin was highly prescribed among patients (Table S1), we next tested whether this drug had an impact on platelet driven thrombin generation in our assay, using platelets from healthy control volunteers. No effect of in vitro aspirin was seen, indicating that supplementation was not reducing the ability of platelet membranes to stimulate thrombin generation (Figure S8 D). Overall, this data suggests that platelet membrane-driven thrombin generation is not responsible for increased thrombotic risk in ACS.

### Platelets externalize aPL on activation, with an unchanged molecular composition between disease groups and healthy controls

Next, the pro-coagulant PL composition of platelets was determined using LC/MS/MS. The aPL species detected were as previously found in healthy control platelets, namely: PE P-16:0_20:4, PE P-18:1_20:4, PE 18:0_20:4, PE P-18:0_20:4, PE 18:0_18:1, PS 18:1_18:1, PS 18:0_18:1 and PS 18:0_20:4, as previously shown^27^ (Figure 5 B, Figure S10 B). Following thrombin activation, platelets from all groups externalized aPL with the most abundant isomers detected in higher amounts on the outside (Figure 5 C, Figure S10 A).

**Figure 6:**
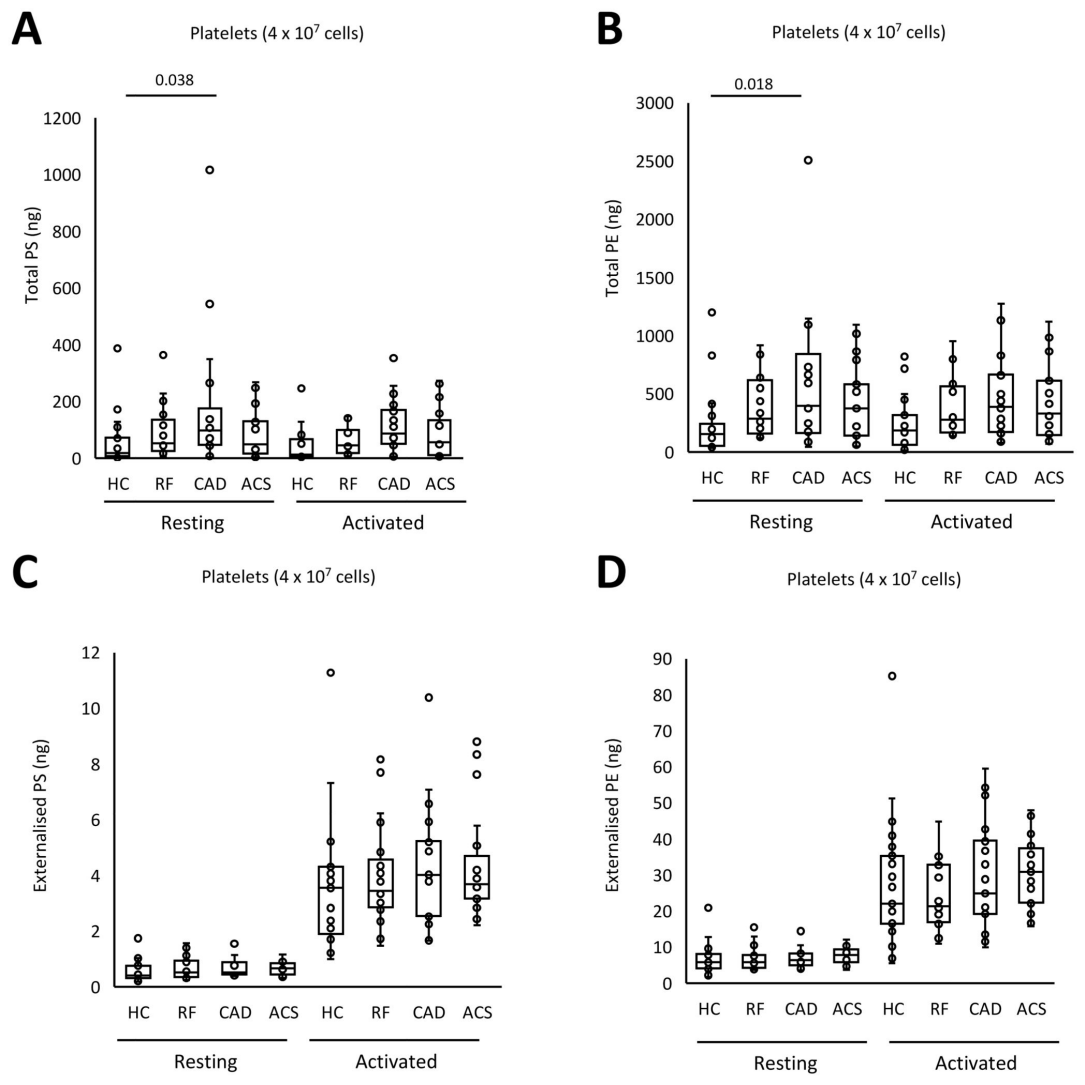
Minimal differences in aPL externalization between groups were seen *Panels A-D: Total and externalized PE and PS for platelets*. Lipids were extracted from resting or thrombin-activated platelets as in Methods. Lipids amounts (ng) were determined using LC/MS/MS. Statistical significance was tested with one way ANOVA and Tukey Post Hoc test (p <0.05 considered significant). ACS: acute coronary syndrome (n = 19 panels A,B, n=21 panels C,D), CAD: coronary artery disease but no ACS (n = 18 panels A,B, n=19 panels C,D), RF: Risk factors with no significant coronary artery disease (n = 17 panels A,B, n=22 panels C,D), HC: Healthy control (n=23).

Total PS and PE levels were somewhat higher in platelets from the CAD group compared to HC in resting and activated conditions (Figure 6 A,B). Following thrombin activation, externalized PS and PE rose compared to resting platelets but there were no significant differences between clinical groups and HC (Figure 6 C,D). Thus, disease did not result in major changes in either the amounts or molecular species of aPL externalized by platelets. Furthermore, there was no correlation between the amount of thrombin generated and externalized PS and PE amounts on the platelet membrane surface (Figure S8 C). In summary, platelets from patients with disease did not have higher PE/PS externalization or elevated thrombin generation capacity suggesting that changes in platelet aPL do not significantly contribute to the higher thrombotic risk seen in cardiovascular disease.

### Levels of TAT and D-Dimer in plasma are not altered in patient groups

Plasma D-Dimer and thrombin anti-thrombin (TAT) complexes were measured for all participants. There were no differences between groups in D-Dimer levels and for most participants, TATs were undetectable (Figure S11 A,B). Additionally, there was no significant correlations between D-Dimers and TATs (Figure S11 C) nor with the amount of thrombin generated on the surface of EV, platelets or leukocytes (Figure S11 D). Finally, the levels of plasma D-Dimer and TAT showed no significant correlations with total or externalized aPL in EV, platelets or leukocytes (Figures S12,13).

## Discussion

Thrombin generation on the plasma membrane surface relies on the presence of procoagulant lipids, primarily native PS and PE, but up to now it was not known whether thrombin generation or native aPL are altered in arterial thrombosis. This is important as it may influence the procoagulant status of patients with this condition and lead to further thrombotic complications^12,13^. Here, thrombin generation and the aPL composition of circulating blood cells and EV from patients with ASCVD was characterized. Our data suggest that EV and leukocyte membranes may contribute to increased thrombotic tendency of patients with cardiovascular disease. In contrast, a role for platelets in driving membrane-dependent coagulation was not revealed. This has implications for the clinical management of ACS which currently does not target leukocyte or EV membrane compartments therapeutically for reducing thrombotic risk.

The assay employed in this study to assess thrombin generation is TF independent and no external PL was added. The means that the assay evaluates the role that the procoagulant membrane PL surface has on prothrombinase activity and is independent of changes in the levels of the patient’s coagulation factors. Whilst there were minimal differences in aPL amounts on the surface of EV and leukocytes between disease groups and HC as characterized by LC/MS/MS, the higher count of EV in plasma provided more surface area for the prothrombinase reaction to take place which in turn led to more thrombin generation. The same is likely for leukocytes which were found to be higher in patients with ACS compared with RF. Together, these results imply that a simple abundance of circulating procoagulant surfaces is sufficient to alter the amount of thrombin generation in resting states. Targeting EV clearance or shielding the circulation from the aPL membrane may therefore present a novel angle to reduce coagulation reactions in this thrombotic condition.

The use of LC/MS/MS to characterize aPL in platelets, leukocytes and EV has not been employed in arterial thrombosis patients prior to this study. Additionally, the external aPL lipidome in leukocytes and EV has not been previously characterized with LC/MS/MS. The majority of the literature on aPL trafficking and detection utilizes a flow cytometry-based method which relies on aPL-binding fluorescent probes^36–38^. The commonest of these is annexin V-FITC, which can bind to either PS or PE in the presence of calcium ^37,38^. There are a number of limitations to this method, the main one being its non-quantitative nature. The binding of annexin V probes to cells and EV during flow cytometry exhibits rapidly saturated kinetics, likely as a result of steric hindrance preventing additional aPL from binding to this large protein^27^. Consequently, whilst it is feasible to count annexin V^+ve^ cells and EV using this method, it is not possible to quantify how much aPL is on the surface, distinguish between PS and PE or know what molecular species are exposed. This is relevant since the procoagulant activity of aPL can be influenced by fatty acyl composition, with an impaired ability of PE comprising shorter FA chains to support coagulation ^27^. Mass spectrometry with biotin derivatization allows the quantitative analysis of aPL molecular species, distinguishing between external and total aPL amounts ^35^. Our study provides a first characterization of PS and PE species present on circulating membrane surfaces using an assay that distinguishes between total and externalized aPL^35^. The findings build on the current understanding of aPL externalization which had previously been reported in ACS patients using non-quantitative techniques with annexin V/lactadherin binding^39^.

Several studies have described higher numbers of EV in patients suffering from arterial thrombosis and its risk factors^40–46^. Hypertensive patients have more circulating EV which correlate with their blood pressure readings and are thought to be generated as a consequence of higher shear^40^. Similarly, diabetes mellitus is associated with higher levels of EV released as a consequence of stimuli such as advanced glycation products and oxidative stress, with a higher procoagulant phenotype in patients with poor glycemic control^41,42^. Patients with dyslipidemias have upregulated EV levels due to LDL-induced membrane blebbing^43^. Indeed, the development of atherosclerosis may be influenced by EV, generated by mechanisms described above, which alter the profile of adhesion molecules on endothelial cells and promote monocyte transmigration and vascular inflammation^47,48^. In addition, the presence of high amounts of EV within the atherosclerotic plaque may contribute to the thrombotic process that follows plaque rupture^49^. This may implicate EV in ACS where elevated numbers were demonstrated in comparison to CAD patients^44–46^, and shown to positively correlate with high-risk coronary angiographic features^50^. Previous studies characterizing plasma from ASCVD patients showed that the highest proportion of EVs in these groups is platelet-derived (40-50%), followed by endothelial-derived (20-25%) then leukocyte-derived (5-10%)^51^. Neutrophil derived EV comprise a relatively low %, being around 5% in ACS and CAD, versus less than 2% in HC^51,52^. Nevertheless, beyond their proposed use as biomarkers and their elevated amounts, the exact role and regulation of EV in human arterial thrombosis remains largely unstudied.

Our data shows no significant changes in platelet-supported thrombin generation or platelet PE/PS externalization. These findings suggest a lack of role for the platelet membrane in raising thrombotic risk in these patients. Notably, the purified factor-based experimental system we used to study membrane involvement does not take into account the many other potential platelet-dependent mechanisms including platelet aggregation, release of platelet-derived EVs, or secondary processes such as release of bioactive mediators that activate leukocytes (thromboxanes) or stimulate vascular and circulating blood cells to generate EVs. Indeed anti-platelet agents such as GP2b3a inhibitors work well in patients with a heavy thrombus burden supporting their central role^53^.

Our study adds significantly to knowledge of EV biology in vascular disease, providing the first characterization of their aPL composition and how this contributes directly to thrombotic tendency. It is generally accepted that annexin V^+ve^ EV (containing externalized aPL) are procoagulant. Previous studies have concluded that a higher number of EV implies more PS exposure in the circulation which in turn could lead to a more procoagulant phenotype and our study adds significant new information to support this idea by characterizing the specific molecular species of PS and PE involved ^45,54,55^. Up to now, methods used to study PS^+ve^ EV have been limited to flow cytometry-based assays with annexin V. This does not allow accurate quantification of external aPL, individual molecular species, nor does it determine the total aPL content within membranes. LC/MS/MS and biotin derivatization allowed the mapping specific molecular species and their amounts, whilst also distinguishing between external and total membrane aPL ^35^.

In our study, TATs and D-Dimers were similar across all groups. However published literature is conflicting, with some studies showing elevations and several others not showing changes in coagulation markers in ASCVD patients ^56–62^. A possible explanation for no increase being seen in this cohort is that many patients were actively taking medications designed to dampen thrombosis via inhibiting platelet activation. However, considering these patients are still at elevated thrombotic risk, the characterisation of the role of additional factors such as circulating PL membranes becomes important.

The timing of any intervention targeting leukocytes or EV in ACS would require careful consideration. In this study, all ACS samples were taken within 48 h of the onset of the event, and therefore the findings reflect the acute phase. This immediate stage is associated with higher thrombotic risk^63^. As such, it is managed with more intensive anti-thrombotic treatment in the first few days after ACS such as the addition of a low molecular-weight heparin to dual anti-platelet therapy^63^. It is unclear however whether the higher procoagulant potential of leukocyte/EV membranes continues in the months after the acute event where persistent activation of the coagulation system have been described in ACS^64^. To test this, longitudinal studies of patients with ACS in the acute (48 hours), intermediate (6 weeks) and distant (6 months) phase would provide more information on the variation of procoagulant membrane properties over time.

### Study Limitations

Whilst cells from patients with ACS may demonstrate lipidomic and biological differences, it is not possible to determine causality since it is not possible to predict when an ACS event will occur^65^. Furthermore, the EV cell of origin and TF surface content is unknown. Two separate preps for EV were utilized, for the coagulation assays and EV counting. This was necessary since EV for the coagulation assay needed to be generated and used on the same day, while EV for counting could be generated later using frozen plasma. Finally, the cross-sectional design of the study on the clinical cohort and the absence of longitudinal sampling timepoints (particularly for the acute phase of ACS) may be confounded by inter-individual variations which may reduce statistical power. This, alongside the relatively small sample size, makes further validation and replication in a larger cohort advantageous.

## Conclusion

In this study, the procoagulant membrane in circulating blood cells and EV was determined in patients with ASCVD. Our findings provide the first characterization of aPL in ASCVD circulating membranes and propose that higher membrane procoagulant activity in arterial thrombosis will be driven primarily by EV and leukocytes. Expanding on these findings, and testing whether interference with the procoagulant lipidome alters the observed incidence of arterial thrombosis would move the field closer towards targeting PL as therapeutic targets for the prevention of thrombosis in high-risk groups.

## Supplementary Material

Dataset

Graphic Abstract

Supplemental File

## Data Availability

The authors declare that all supporting data are available within the article and its online supplementary files. Raw data for individual experiments are available from the corresponding authors on reasonable request.

## Highlights

- The aPL profile of membranes from platelets, leukocytes and EV in patients with ASCVD is characterized in detail using lipidomics, and EV aPL levels were found to be generally higher in ASCVD groups than HC.
- Thrombin generation, EV counts and aPL levels are elevated in EV membranes of patient groups as compared to healthy controls suggesting these particles interact directly with coagulation factors to drive thrombotic risk in ASCVD.
- Membranes from leukocytes were also suggested to contribute somewhat, although platelet aPL did not appear to drive elevated thrombin generation in patients.

## Clinical Perspective

**What are the clinical implications?**

- The EV aPL membrane is proposed as a therapeutic target for dampening thrombotic risk in ASCVD.
- Targeting circulating aPL membrane levels or blocking their interaction with coagulation factors could reduce thrombotic risk in ASCVD.
