# Supplementary figures and images for: Thrombin generation is associated with extracellular vesicle and leukocyte lipid membranes in atherosclerotic cardiovascular disease
Source: Arterioscler Thromb Vasc Biol. Author manuscript; Available in PMC 2024 Sep 1. (PMC11335086; doi:10.1161/ATVBAHA.124.320902)

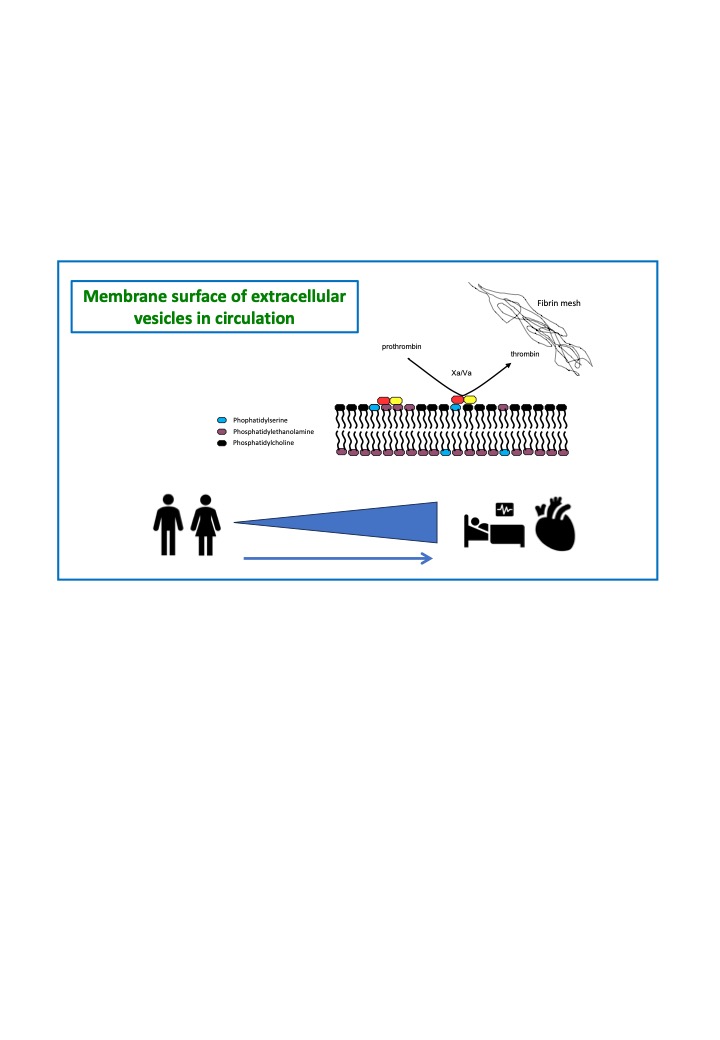

Supplement: Graphic Abstract [file EMS197530-supplement-Graphic_Abstract.jpg]
