# Supplementary material for: Thrombin generation is associated with extracellular vesicle and leukocyte lipid membranes in atherosclerotic cardiovascular disease
Source: Arterioscler Thromb Vasc Biol. Author manuscript; Available in PMC 2024 Sep 1. (PMC11335086; doi:10.1161/ATVBAHA.124.320902)
Supplement: Supplemental File [file EMS197530-supplement-Supplemental_File_.pdf]

SUPPLEMENTAL MATERIALS

Thrombin generation is associated with extracellular vesicle and leukocyte lipid membranes in atherosclerotic cardiovascular disease.

Majd B Protty[1]*, Victoria J Tyrrell[1], Keith Allen-Redpath[2], Shin Soyama[2], Ali A Hajeyah[1], Daniela Costa[1], Anirban Choudhury[3], Rito Mitra[4], Amal Sharman[2], Parveen Yaqoob[2], P Vince Jenkins[1], Zaheer Yousef[4], Peter W Collins[1] and Valerie B O'Donnell[1]*

[1] Systems Immunity University Institute, Cardiff University, Cardiff, CF14 4XN, UK
[2] Department of Nutritional Sciences, University of Reading, Reading, RG6 6AP, UK
[3] Morriston Cardiac Centre, Swansea Bay University Health Board, Swansea, SA6 6NL, UK
[4] Department of Cardiology, University Hospital of Wales, Cardiff, CF14 4XW, UK

Supplementary Figures and Tables.

Supplementary Figure Legends.

Figure S1. The experimental design of studies of arterial thrombosis in the clinical cohort. Patients were recruited as described in Methods. Blood was taken in a clinical area, and was transferred immediately to the laboratory. Platelets, leukocytes and extracellular vesicles (EV) were separated as described in Methods. The cells were divided into fractions to undergo functional testing with prothrombinase assay and lipid extraction and processing with LC/MS/MS, as outlined in Methods.

Figure S2. The workflow of the prothrombinase assay used to determine the procoagulant potential of washed cells and extra-cellular vesicles. In step 1, a known number of cells (platelets, leukocytes or EV) was aliquoted into a 96-well half-area plate. In step 2, a coagulation factor mixture containing calcium, FII, FXa, FVa was added to the plate to start the prothrombinase reaction on the surface of cells. The reaction was allowed to proceed for 5 min (step 3), before being quenched with EDTA (step 4). The amount of thrombin (FIIa) made was quantified using a p-nitroaniline (pNA) containing chromogenic substrate S-2238 and absorbance read (405 nm) and compared to a standard curve of human thrombin (step 5).

Figure S3. LC/MS/MS chromatograms of biotinylated-PS molecular species. Representative chromatograms from participant samples are shown for the PS molecular species quantified with the QTrap 4000 as in Methods. MRM transition and lipid name are displayed at the top of the chromatogram panels. These represent typical signals obtained from experiments performed.

Figure S4. LC/MS/MS chromatograms of biotinylated-PE molecular species. Representative chromatograms from participant samples are shown for the PE molecular species quantified with the QTrap 4000 as in Methods. MRM transition and lipid name are displayed at the top of the chromatogram. These represent typical signals obtained from experiments performed.

Figure S5: EV preparation is not contaminated with lipoproteins and lipoproteins elute later than EV during SEC, while lipoproteins isolated by density gradient centrifugation elute from SEC in later fractions, separate from EV. *Panel A: EV preparation is not contaminated with lipoproteins.* Paired samples of double-spun plasma or EV-rich fractions from healthy volunteers as described in Methods were analyzed for the presence of Apolipoprotein B (ApoB) using an ELISA kit (Abcam, UK) (marker for low density lipoproteins), n=3, mean +/- SEM*. Panels B,C: Chylomicrons (ApoB 48) and VLDL (ApoE 100) elute later than EV during SEC.* Platelet free plasma (PFP) was subject to SEC, and elution of ApoB 48 and ApoB 100 monitored using ELISAs as outlined in Supplementary Data (n = 2, mean +/- SD). *Panels D,E,F. Density gradient isolated lipoproteins elute later than EV during SEC.* Lipoproteins (chylomicrons, VLDL-1 and VLDL-2) were isolated using density gradient centrifugation. They were then subject to SEC to determine their elution profile (n = 12, mean +/- SEM).

Figure S6. Significantly smaller EV particles (diameter) are detected in CAD patient plasma, and a downward trend in RF and ACS patients was seen, compared with healthy controls. Platelet-free plasma (0.5 ml) from each participant was processed through size exclusion chromatography (iZON qEV columns) and nanoparticle tracking analysis (Nanosight 300), as in Methods. Data for size was plotted as a box plot with the ggplot2 R package. Statistical significance was tested with one way ANOVA and Tukey Post Hoc test (*: $p < 0.05$, **: $p < 0.01$, ***: $p < 0.001$). ACS: acute coronary syndrome (n=24), CAD: coronary artery disease but no ACS (n=19), RF: Risk factors with no significant coronary artery disease (n=23), HC: Healthy control (n=22).

Figure S7. Characterization of aminophospholipid species in EV from a clinical cohort of patients with arterial thrombosis shows overall similar amounts of externalized aPL between clinical groups. *Panel A: The amounts of externalized aPL species in EV samples were largely similar between clinical groups.* Using EV samples isolated from plasma, the amounts of externalized PS and PE species for each of the clinical groups as quantified by LC/MS/MS were plotted to examine for differences between groups. *Panel B: The amounts of total aPL species in EV samples were elevated in the CAD group.* The

amounts of total PS and PE species as quantified by LC/MS/MS for each of the clinical groups were plotted to examine for differences between groups. Lipids were extracted from EV-rich plasma fractions as in Methods. Lipid amounts (ng) were calculated by LC/MS/MS. Statistical significance was tested with one way ANOVA and Tukey Post Hoc test (*: p <0.05, **: p <0.01, ***: p <0.001). Externalized: ACS: acute coronary syndrome (n=21), CAD: coronary artery disease but no ACS (n=19), RF: Risk factors with no significant coronary artery disease (n=22), HC: Healthy control (n=23). Total: ACS: acute coronary syndrome (n=19), CAD: coronary artery disease but no ACS (n=18), RF: Risk factors with no significant coronary artery disease (n=17), HC: Healthy control (n=23).

Figure S8. Thrombin generation correlated with external PS in EV samples, but no correlation was seen with externalized aPL in resting platelets and leukocytes, and the prothrombinase assay is not affected by aspirin. The ability of EV (A), resting leukocyte (B) and resting platelet (C) membranes to support thrombin generation was assessed using the prothrombinase assay and correlated with externalized PS and PE amounts quantified by LC/MS/MS using Pearson's correlation (n=85). Lipids were extracted as in Materials and Methods. Lipids amounts (ng) were calculated by LC/MS/MS. Statistical significance was tested with Pearson's correlation. *Panel D: Prothrombinase activity on platelets is not affected by aspirin.* The prothrombinase assay was carried out, as described in Methods, on washed platelets from healthy volunteers (n=3) in the presence or absence of 1mM aspirin. Data presented as mean +/- SEM.

Figure S9. Characterization of aminophospholipid species in leukocytes from a clinical cohort of patients with arterial thrombosis shows largely similar profile of aPL lipids across clinical groups. *Panel A: Leukocytes externalize aPL molecular species upon ionophore-activation.* Using washed leukocytes (resting and iono-phore activated), the amounts of externalized PS and PE species for each of the clinical groups as quantified by LC/MS/MS were plotted to examine for differences between groups. *Panel B: Minimal differences in the amounts of total aPL molecular species between clinical groups are seen.* The amounts of total PS and PE species for each of the clinical groups were quantified by LC/MS/MS to examine for differences between groups. Lipids were extracted from washed leukocytes as in Materials and Methods. Lipids amounts (ng) were calculated by LC/MS/MS. Statistical significance was tested with one way ANOVA and Tukey Post Hoc test (*: p <0.05, **: p <0.01, ***: p <0.001). Externalized: ACS: acute coronary syndrome (n=21), CAD: coronary artery disease but no ACS (n=19), RF: Risk factors with no significant coronary artery disease (n=22), HC: Healthy control (n=23). Total: ACS: acute coronary syndrome (n=19), CAD: coronary artery disease but no ACS (n=18), RF: Risk factors with no significant coronary artery disease (n=17), HC: Healthy control (n=23).

Figure S10: Characterization of aminophospholipid species in platelets from a clinical cohort of patients with arterial thrombosis shows a similar profile of aPL externalization between ACS and HC samples. *Panel A: Thrombin-activation increased aPL externalization on the surface of platelets with minimal differences between clinical groups.* Externalized PS and PE species were quantified in platelets (resting or thrombin-activated) by LC/MS/MS for each of the clinical groups, and plotted to examine for differences between groups. *Panel B: Quantification of Total aPL species detected in platelet membranes showed minimal differences between ACS and HC.* The amounts of total PS and PE for each clinical group was plotted to examine for differences between groups. Lipids were extracted from washed platelets as in Materials and Methods. Lipids amounts (ng) were calculated by LC/MS/MS. Statistical significance was tested using one way ANOVA and Tukey Post Hoc test (*: p <0.05, **: p <0.01, ***: p <0.001). Externalized: ACS: acute coronary syndrome (n=21), CAD: coronary artery disease but no ACS (n=19), RF: Risk factors with no significant coronary artery disease (n=22), HC: Healthy control (n=23). Total: ACS: acute coronary syndrome (n=19), CAD: coronary artery disease but no ACS (n=18), RF: Risk factors with no significant coronary artery disease (n=17), HC: Healthy control (n=23).

Figure S11: D-Dimer or TAT levels were similar for all groups and no correlation with thrombin generation on EV, platelets or leukocytes was seen. *Panel A: D-Dimer was measured from stored plasma samples from all participants using a human D-Dimer ELISA kit (Abcam, UK) and plotted on a box plot. Panel B: TATs were measured in stored plasma samples from all participants using a human TAT ELISA kit (Abcam, UK) and plotted on a box plot. Panel C: Correlation plot of D-Dimer and TAT in all*

participants. D: Correlation plot between D-Dimer or TATs and thrombin generation data from EV, platelets and leukocytes. Statistical significance was tested with Pearson's correlation or one way ANOVA and Tukey Post Hoc test (*: p <0.05, **: p <0.01, ***: p <0.001). ACS: acute coronary syndrome (n=24), CAD: coronary artery disease but no ACS (n=19), RF: Risk factors with no significant coronary artery disease (n=23), HC: Healthy control (n=24), TAT: Thrombin Anti-thrombin.

Figure S12: D-Dimer or TATs did not correlate with aPL amounts in EV or leukocytes. *D-Dimer and TATs were measured in stored plasma samples from all participants using ELISA (Abcam, UK) and correlated with aPL amounts from EV and leukocytes and displayed as correlation plots.* Statistical significance was tested with Pearson's correlation (*: p <0.05, **: p <0.01, ***: p <0.001). TAT: Thrombin Anti-thrombin (n=85).

Figure S13: D-Dimer or TATs did not correlated with aPL amounts in platelets. *D-Dimer and TATs were measured in stored plasma samples from all participants using ELISA (Abcam, UK) and correlated with aPL amounts from platelets and displayed as correlation plots.* Statistical significance was tested with Pearson's correlation (*: p <0.05, **: p <0.01, ***: p <0.001). TAT: Thrombin Anti-thrombin (n=85).

Variable	Healthy control (HC) (n=24)	Risk-factor matched (RF) (n=23)	Significant coronary artery disease (CAD) (n=19)	Acute coronary syndrome (ACS) (n=24)	p
Age, Mean ± SD	64.92 ± 10.79	61.43 ± 8.16	64.53 ± 9.65	64.67 ± 9.88	0.473
Male Sex (%)	14 (58.3)	12 (52.2)	16 (84.2)	17 (70.8)	0.133
Creatinine μmol/L, Mean ± SD	-	77.57 ± 12.60	88.39 ± 31.95	84.79 ± 18.64	0.318
Haemoglobin g/dL, Mean ± SD	-	146.04 ± 13.03	138.24 ± 17.95	144.46 ± 18.88	0.380
Platelets x 10^9/L, Mean ± SD	-	250.26 ± 44.01	261.94 ± 56.08	271.50 ± 82.72	0.869
WCC x 10^9/L, Mean ± SD	-	6.96 ± 1.57	7.92 ± 1.92	9.28 ± 2.90	0.011
Neutrophils	-	4.33 ± 1.17	5.02 ± 1.32	6.51 ± 2.49	0.003
Eosinophils	-	0.19 ± 0.09	0.25 ± 0.12	0.11 ± 0.08	<0.001
Basophils	-	0.02 ± 0.04	0.03 ± 0.05	0.00 ± 0.02	0.098
Lymphocytes	-	1.82 ± 0.68	1.89 ± 0.70	1.85 ± 0.71	0.818
Monocytes	-	0.57 ± 0.19	0.68 ± 0.21	0.73 ± 0.32	0.150
RBC x 10^{12}/L, Mean ± SD	-	4.80 ± 0.42	4.14 ± 1.26	4.64 ± 0.54	0.090
Aspirin use (%)	0 (0)	20 (87)	14 (73.7)	24 (100)	0.017
P2Y12 inhibitor use (%)	0 (0)	3 (13)	6 (31.6)	24 (100)	<0.001
Anticoagulant use (%)	0 (0)	3 (13)	0 (0)	24 (100)	-
Statin use (%)	0 (0)	15 (65.2)	15 (78.9)	19 (79.2)	0.532
Hypertension (%)	0 (0)	13 (56.5)	13 (68.4)	11 (45.8)	0.350
Diabetes (%)	0 (0)	7 (30.4)	3 (15.8)	6 (25)	0.554
Smoker (%)	0 (0)	7 (30.4)	9 (47.4)	13 (54.2)	0.244
CKD (%)	0 (0)	0 (0)	3 (15.8)	3 (12.5)	-

Table S1. Baseline clinical characteristics of patients recruited in the clinical cohort. (WCC: white cell count, RBC: red blood cell count, P2Y12 inhibitors: clopidogrel, prasugrel or ticagrelor, CKD: chronic

kidney disease, SD: standard deviation, p-value tests: Fisher exact (categorical) or Kruskal-Wallis (continuous), p-value comparators: all clinical groups (age, sex) or all except HC for other variables).

Analyte	Mass	Biotinylated mass	*m/z* [M-H]$^{-}$	Biotinylated MRM transition	DP (V)	CE (V)	CXP (V)
PE 14:0_14:0 (DMPE)	635	861	860	860→227	−135	−60	−13
PS 14:0_14:0 (DMPS)	679	905	904	904→591	−150	−42	−17
PE P-18:0_20:4 (SpAPE)	751	977	976	976→303	−160	−60	−5
PE 18:0_20:4 (SAPE)	767	993	992	992→303	−170	−58	−5
PE P-16:0_20:4 (PpAPE)	723	949	948	948→303	−160	−60	−5
PE 18:0_18:1 (SOPE)	745	971	970	970→281	−170	−58	−5
PE P-18:1_20:4 (OpAPE)	749	975	974	974→303	−160	−60	−5
PS 18:0_18:1 (SOPS)	789	1,015	1,014	1,014→701	−140	−44	−23
PS 18:1_18:1 (DOPS)	787	1,013	1,012	1,012→699	−150	−46	−23
PS 18:0_20:4 (SAPS)	811	1,037	1,036	1,036→723	−145	−42	−23

Table S2. Multiple reaction monitoring (MRM) transitions for the aPL analyzed in negative ion mode as part of targeted lipidomic analysis of cells isolated from patients with atherothrombosis.

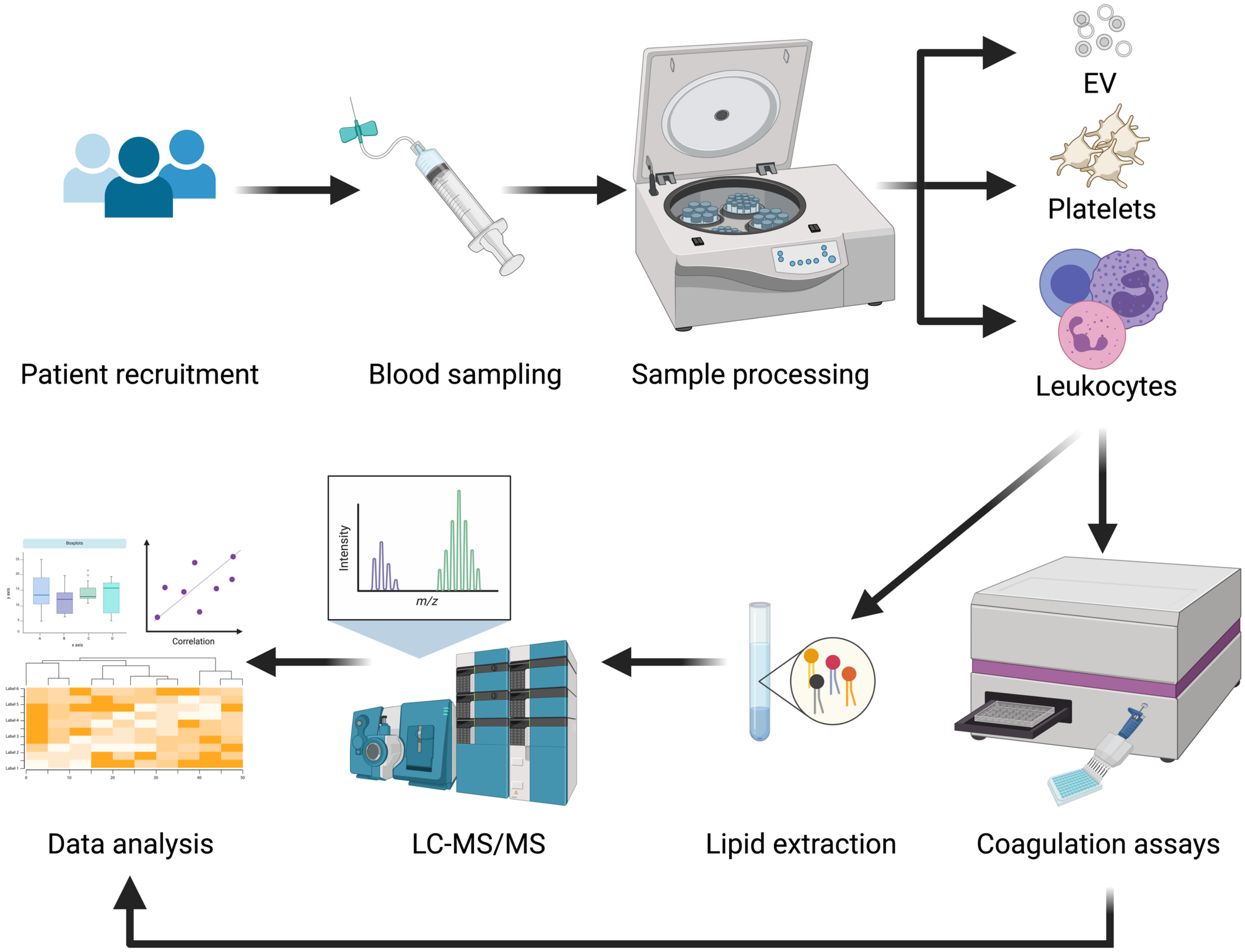

EV
Platelets
Leukocytes
Patient recruitment
Blood sampling
Sample processing
Intensity
m/z
Boxplots
Correlation
Data analysis
LC-MS/MS
Lipid extraction
Coagulation assays

Figure S1

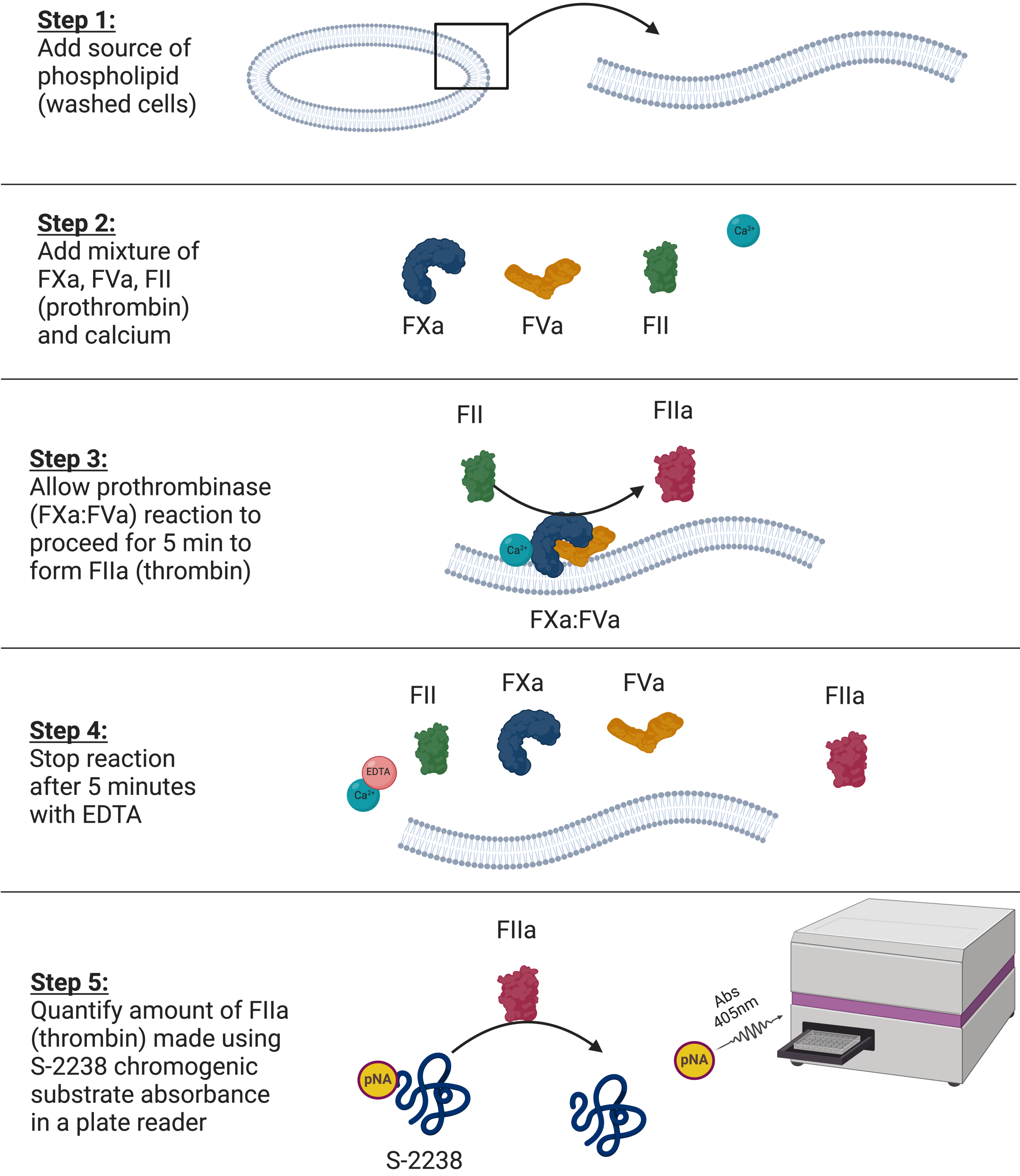

Step 1:
Add source of phospholipid (washed cells)
Step 2:
Add mixture of FXa, FVa, FII (prothrombin) and calcium
Ca2+
FXa
FVa
FII
Step 3:
Allow prothrombinase (FXa:FVa) reaction to proceed for 5 min to form FIIa (thrombin)
FII
FIIa
Ca2+
FXa:FVa
Step 4:
Stop reaction after 5 minutes with EDTA
FII
FXa
FVa
FIIa
EDTA
Ca2+
Step 5:
Quantify amount of FIIa (thrombin) made using S-2238 chromogenic substrate absorbance in a plate reader
FIIa
pNA
S-2238
Abs 405nm
pNA

Figure S2

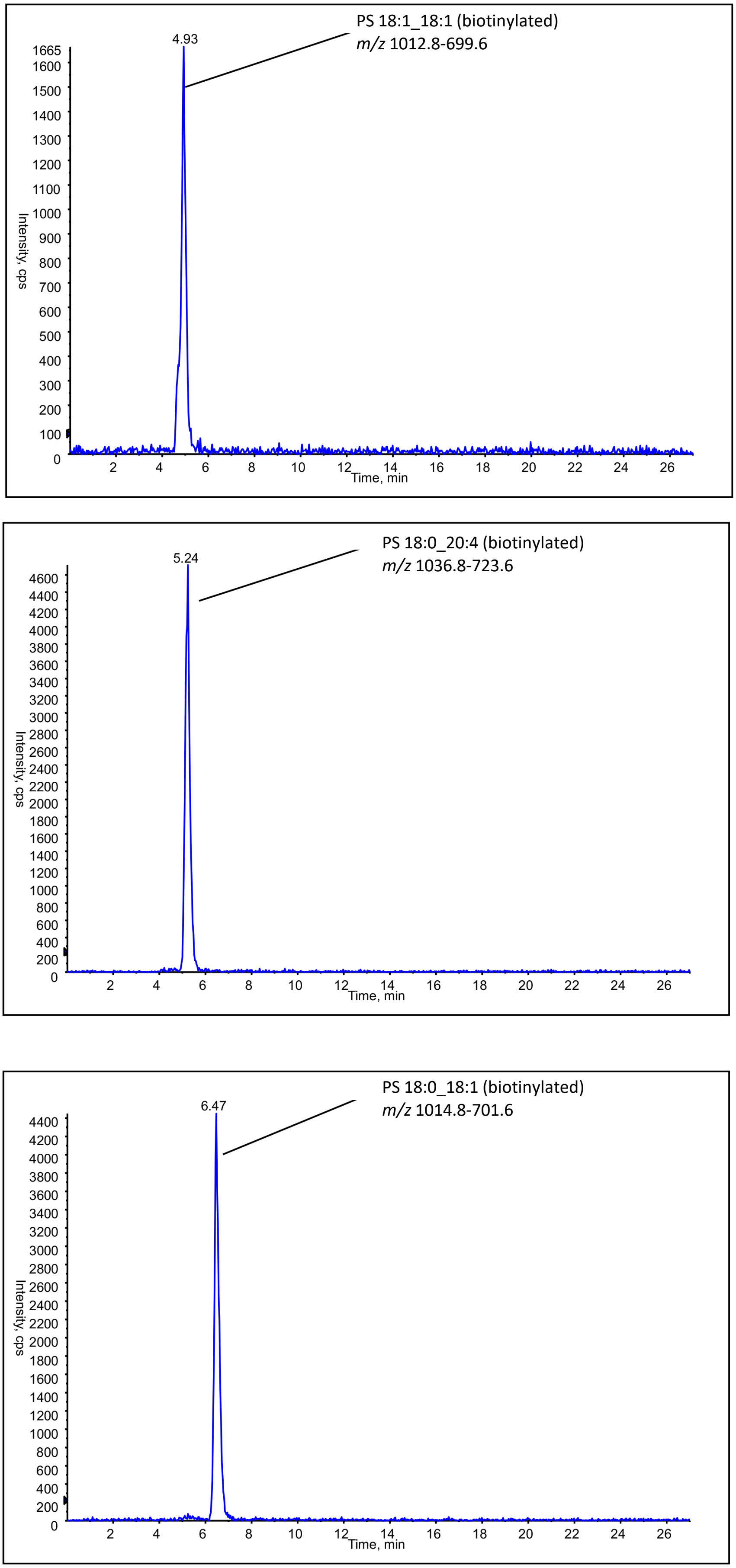
PS 18:1_18:1 (biotinylated)
m/z 1012.8-699.6
4.93
Intensity, cps
Time, min
PS 18:0_20:4 (biotinylated)
m/z 1036.8-723.6
5.24
Intensity, cps
Time, min
PS 18:0_18:1 (biotinylated)
m/z 1014.8-701.6
6.47
Intensity, cps
Time, min

Figure S3

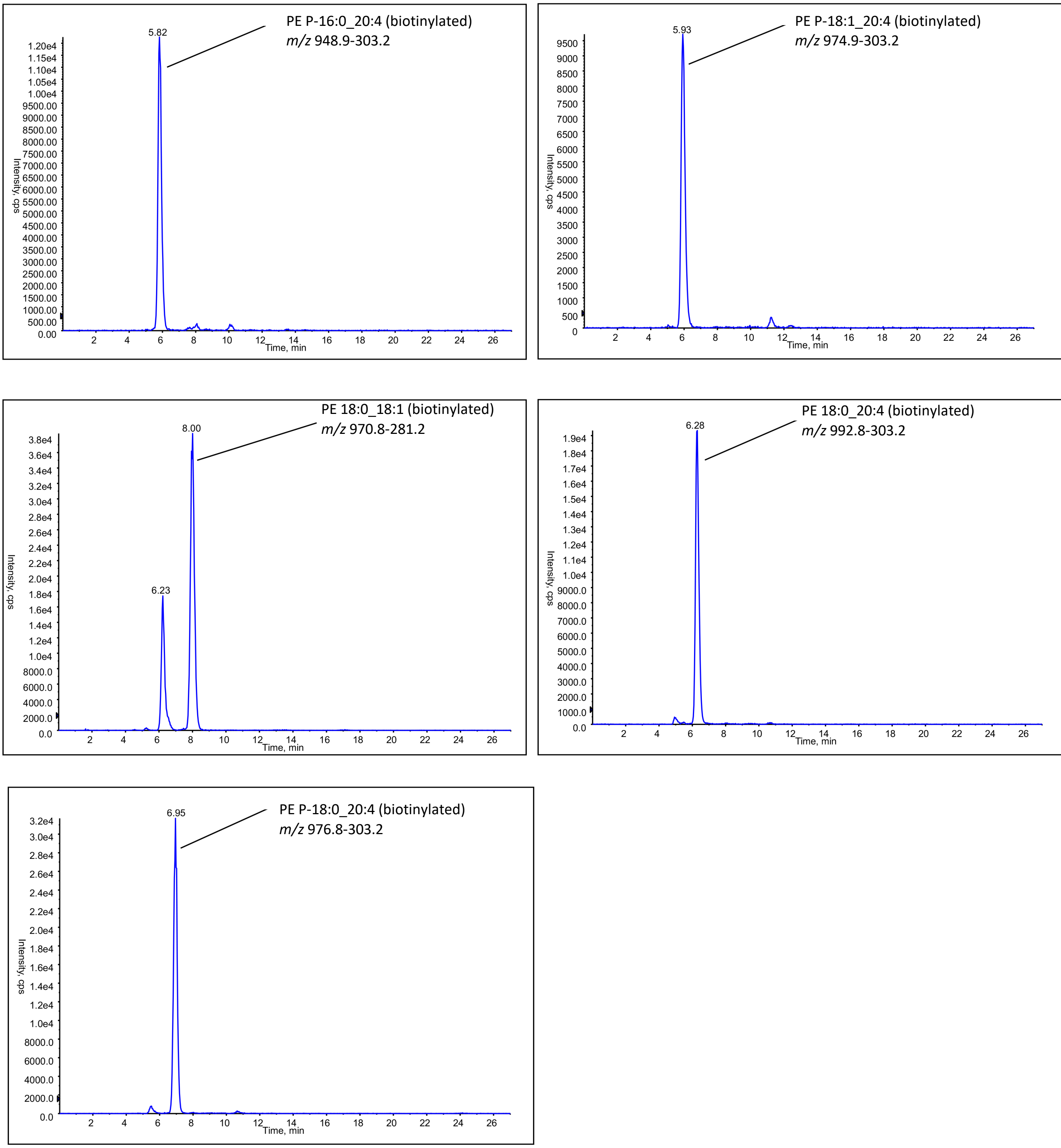
PE P-16:0_20:4 (biotinylated)
m/z 948.9-303.2
5.82
PE P-18:1_20:4 (biotinylated)
m/z 974.9-303.2
5.93
PE 18:0_18:1 (biotinylated)
m/z 970.8-281.2
8.00
6.23
PE 18:0_20:4 (biotinylated)
m/z 992.8-303.2
6.28
PE P-18:0_20:4 (biotinylated)
m/z 976.8-303.2
6.95
Intensity, cps
Time, min

Figure S4

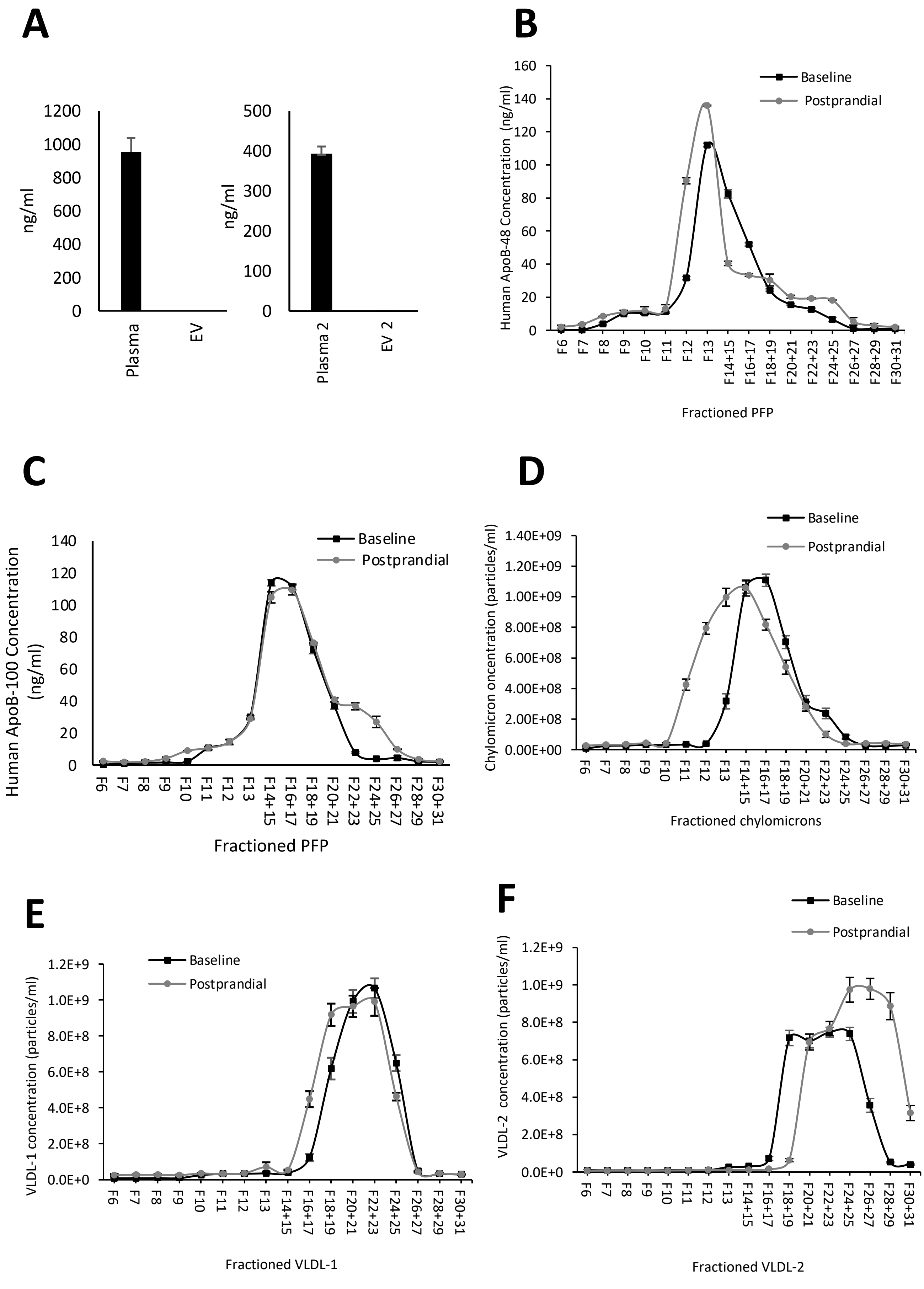
A
B
C
D
E
F
ng/ml
Plasma
EV
Plasma 2
EV 2
Human ApoB-48 Concentration (ng/ml)
Fractioned PFP
Human ApoB-100 Concentration (ng/ml)
Chylomicron oncentration (particles/ml)
Fractioned chylomicrons
VLDL-1 concentration (particles/ml)
Fractioned VLDL-1
VLDL-2 concentration (particles/ml)
Fractioned VLDL-2
Baseline
Postprandial

Figure S5

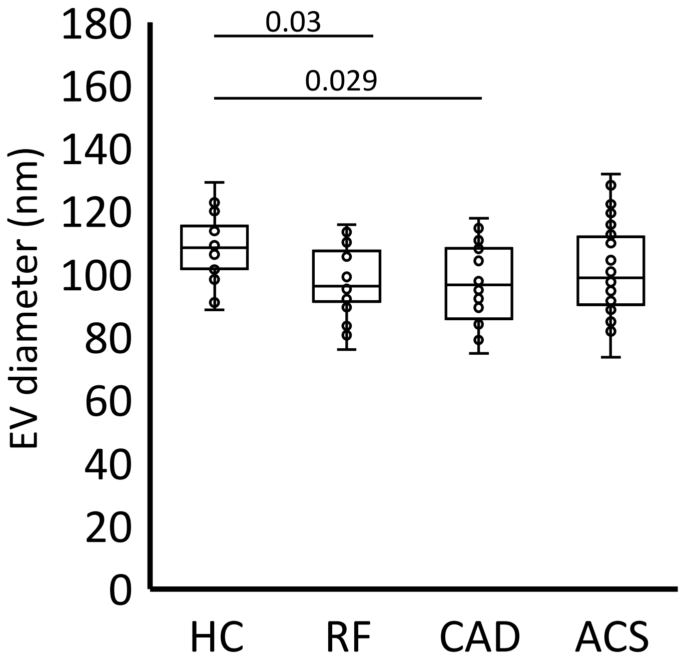

0.03
0.029
180
160
140
120
100
80
60
40
20
0
EV diameter (nm)
HC
RF
CAD
ACS

Figure S6

A – externalized aPL in EV samples

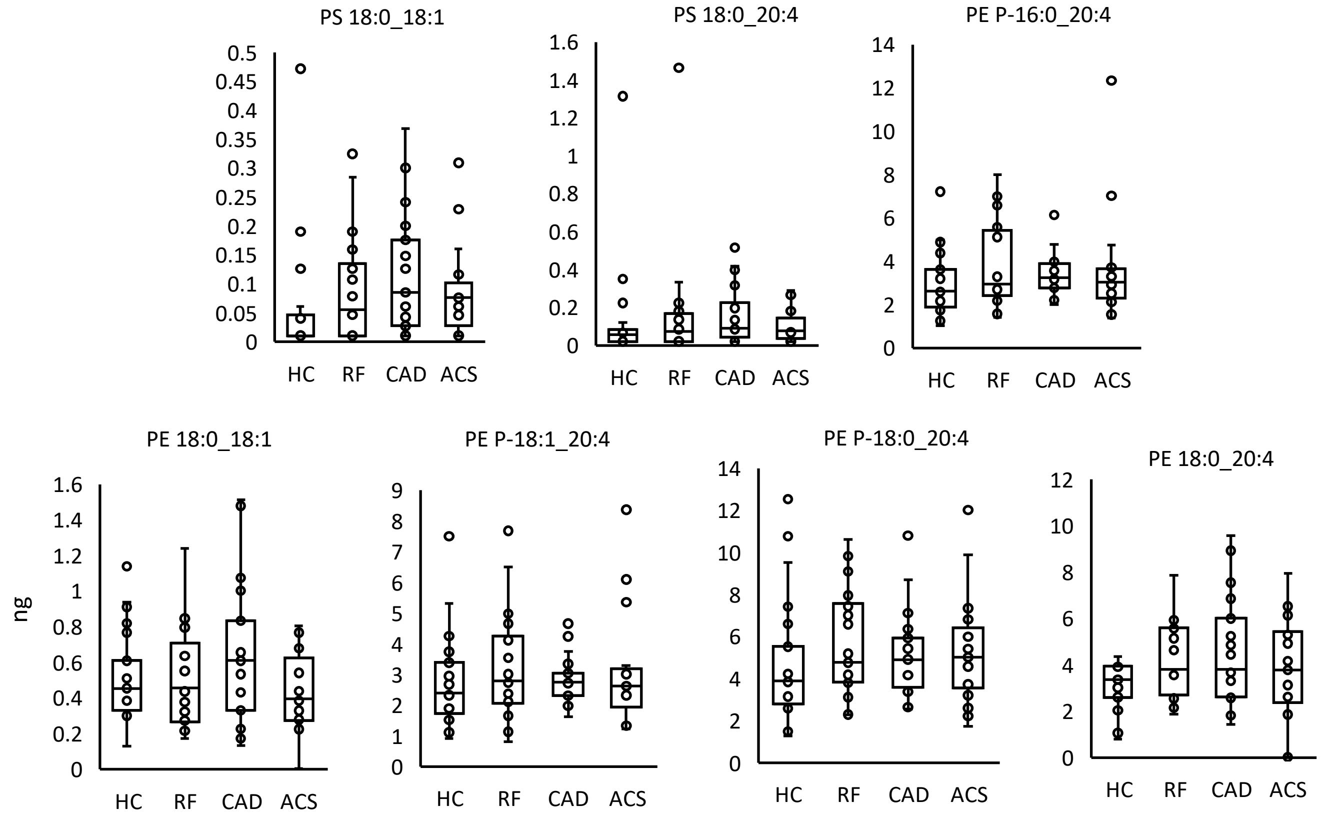

B – total aPL in EV samples

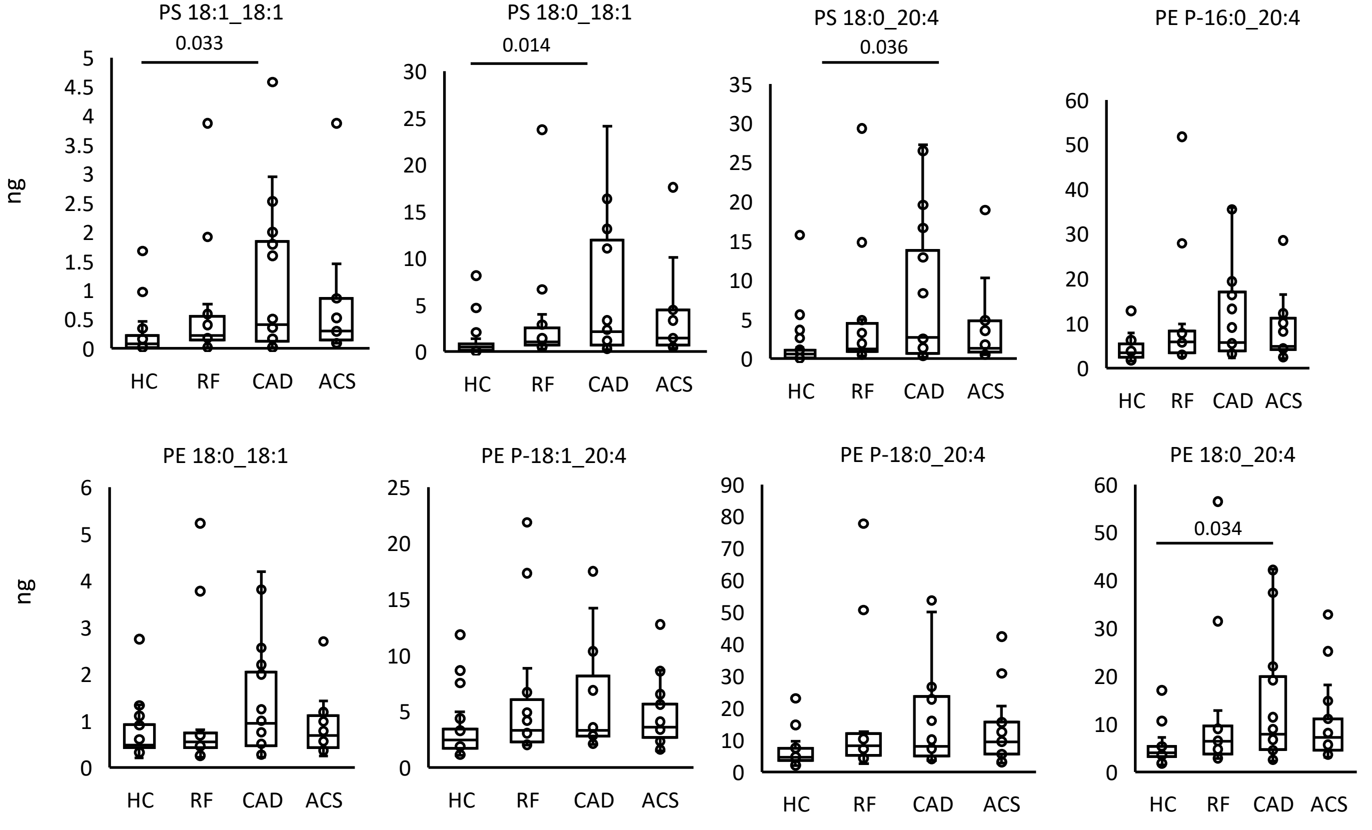

Figure S7

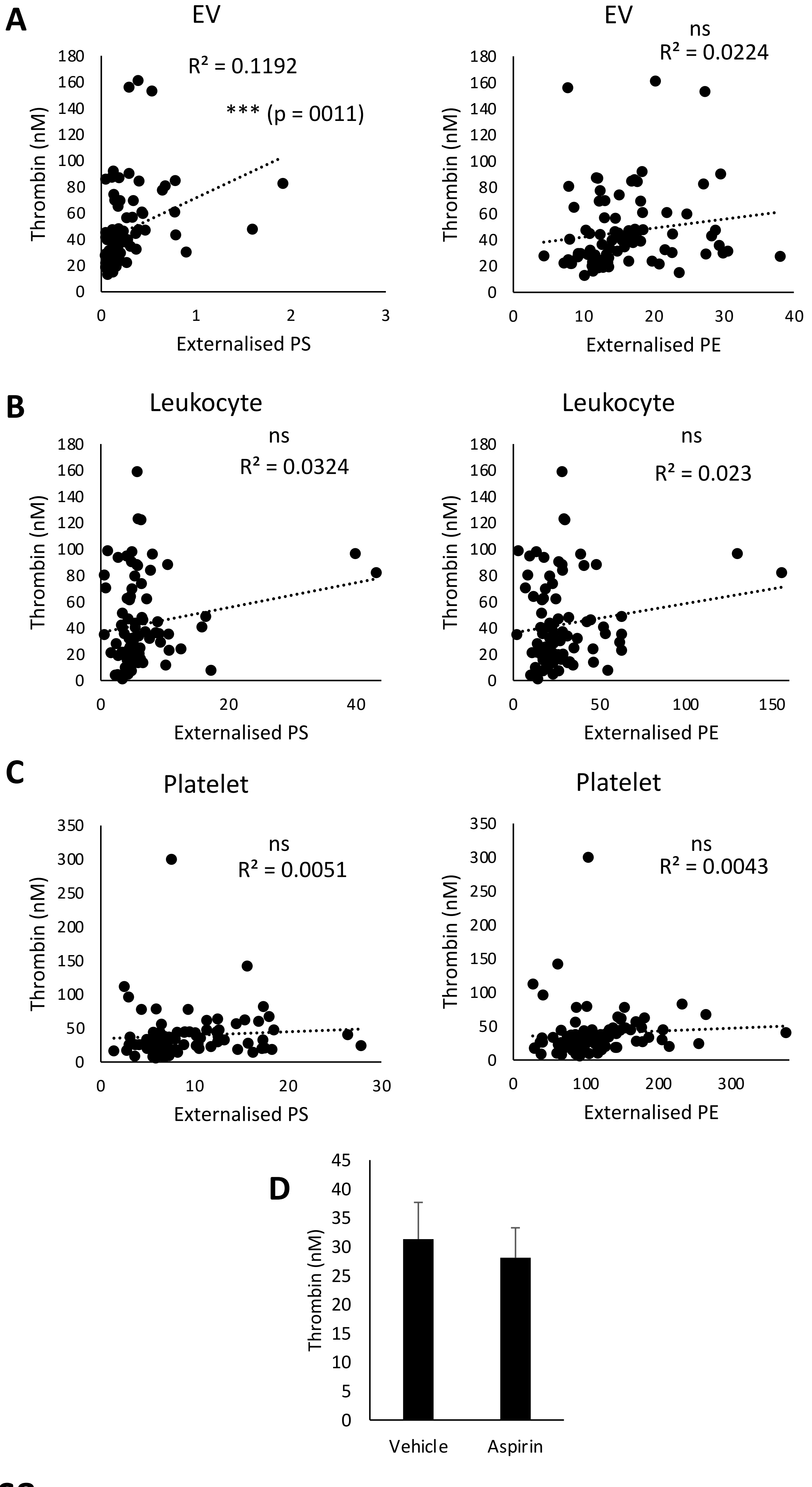
A
EV
R² = 0.1192
*** (p = 0011)
Thrombin (nM)
Externalised PS
EV
ns
R² = 0.0224
Thrombin (nM)
Externalised PE
B
Leukocyte
ns
R² = 0.0324
Thrombin (nM)
Externalised PS
Leukocyte
ns
R² = 0.023
Thrombin (nM)
Externalised PE
C
Platelet
ns
R² = 0.0051
Thrombin (nM)
Externalised PS
Platelet
ns
R² = 0.0043
Thrombin (nM)
Externalised PE
D
Thrombin (nM)
Vehicle
Aspirin

Figure S8

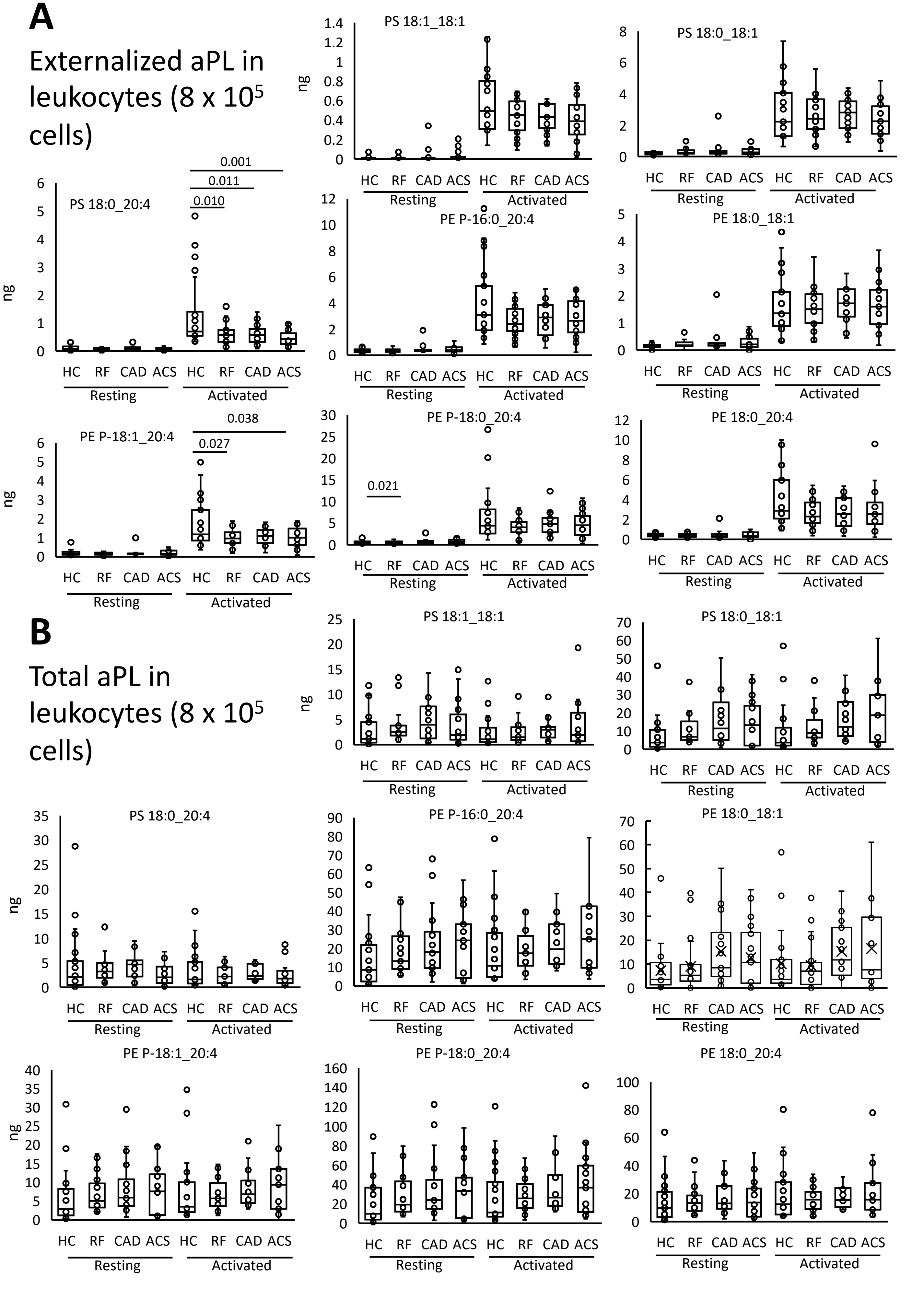
A
Externalized aPL in leukocytes (8 x 10⁵ cells)
PS 18:1_18:1
PS 18:0_18:1
PS 18:0_20:4
PE P-16:0_20:4
PE 18:0_18:1
PE P-18:1_20:4
PE P-18:0_20:4
PE 18:0_20:4
0.001
0.011
0.010
0.038
0.027
0.021
ng
HC RF CAD ACS HC RF CAD ACS
Resting
Activated
B
Total aPL in leukocytes (8 x 10⁵ cells)
PS 18:1_18:1
PS 18:0_18:1
PS 18:0_20:4
PE P-16:0_20:4
PE 18:0_18:1
PE P-18:1_20:4
PE P-18:0_20:4
PE 18:0_20:4

Figure S9

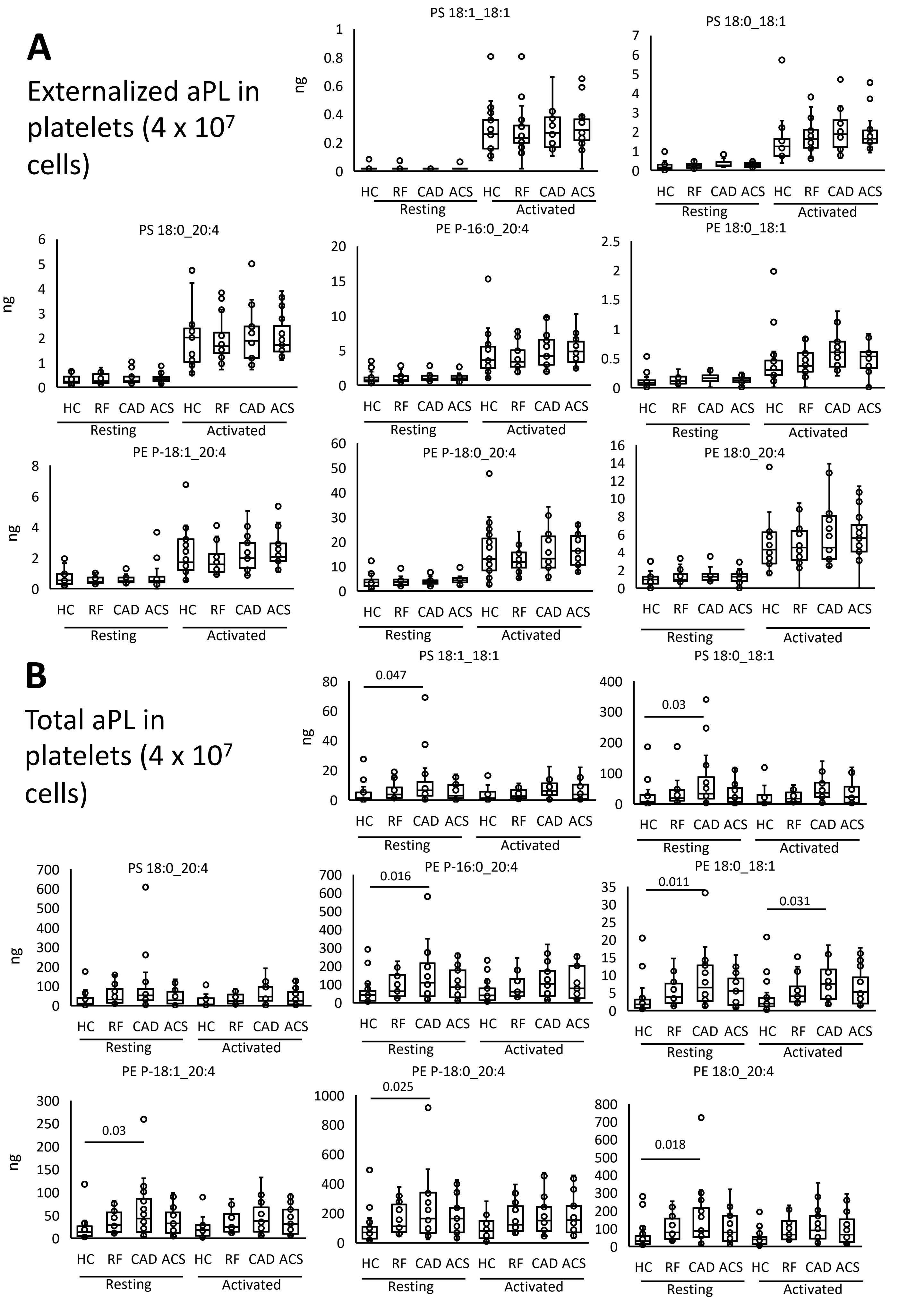

Supplementary Figure 10

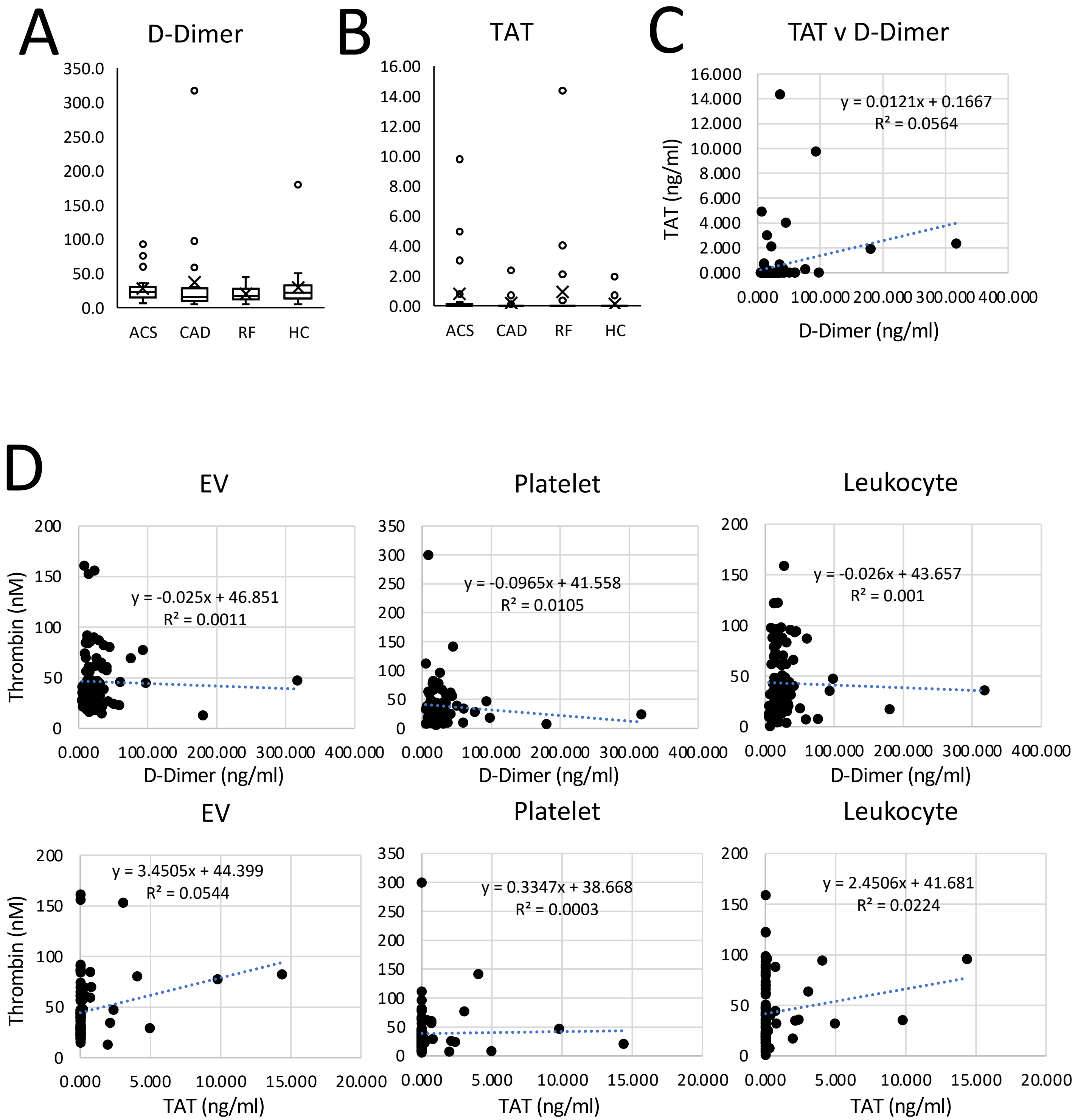
A
D-Dimer
350.0
300.0
250.0
200.0
150.0
100.0
50.0
0.0
ACS
CAD
RF
HC
B
TAT
16.00
14.00
12.00
10.00
8.00
6.00
4.00
2.00
0.00
ACS
CAD
RF
HC
C
TAT v D-Dimer
TAT (ng/ml)
16.000
14.000
12.000
10.000
8.000
6.000
4.000
2.000
0.000
y = 0.0121x + 0.1667
R² = 0.0564
0.000
100.000
200.000
300.000
400.000
D-Dimer (ng/ml)
D
EV
Thrombin (nM)
y = -0.025x + 46.851
R² = 0.0011
D-Dimer (ng/ml)
Platelet
y = -0.0965x + 41.558
R² = 0.0105
D-Dimer (ng/ml)
Leukocyte
y = -0.026x + 43.657
R² = 0.001
D-Dimer (ng/ml)
EV
Thrombin (nM)
y = 3.4505x + 44.399
R² = 0.0544
TAT (ng/ml)
Platelet
y = 0.3347x + 38.668
R² = 0.0003
TAT (ng/ml)
Leukocyte
y = 2.4506x + 41.681
R² = 0.0224
TAT (ng/ml)

Figure S11

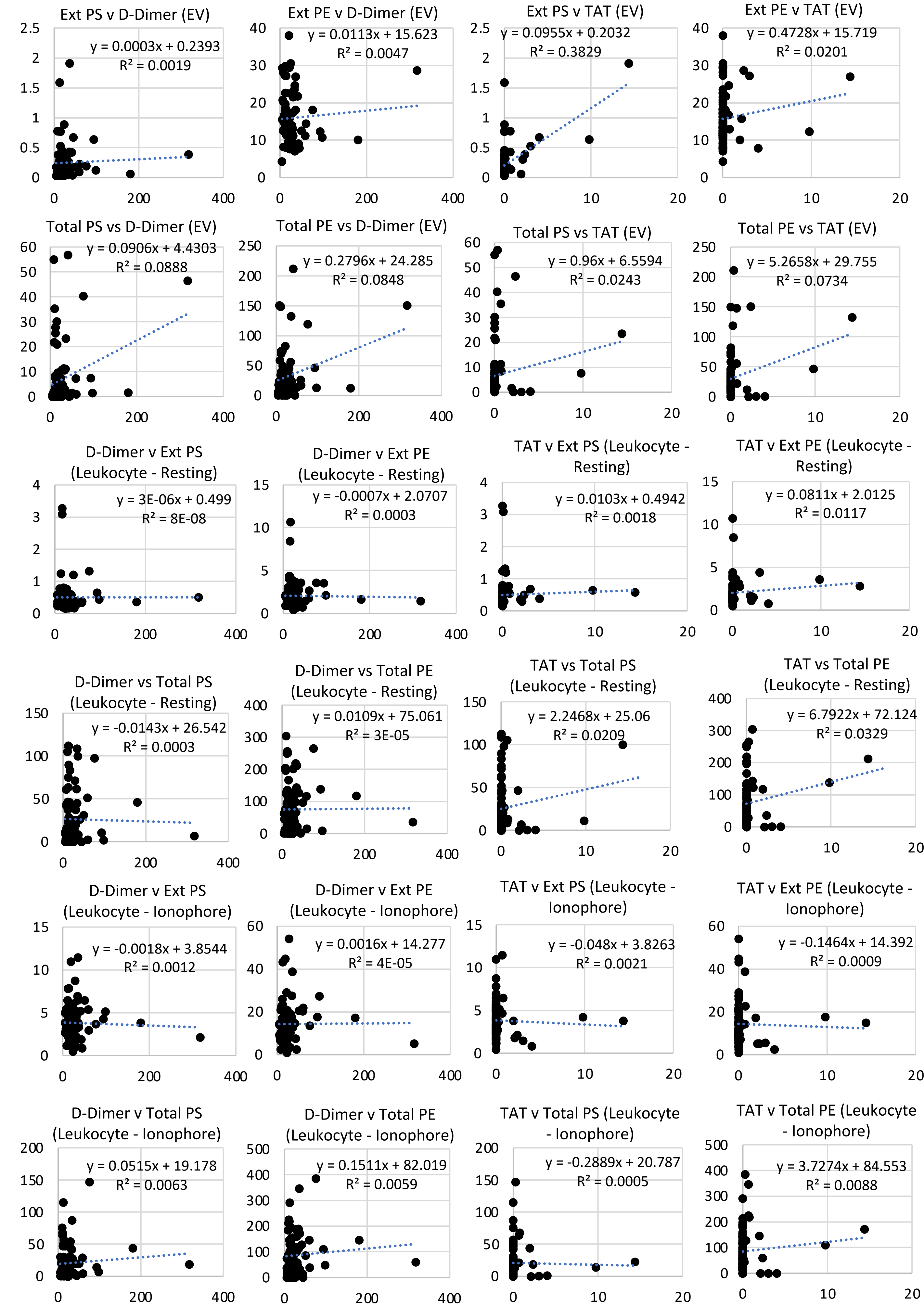
Ext PS v D-Dimer (EV)
y = 0.0003x + 0.2393
R² = 0.0019
Ext PE v D-Dimer (EV)
y = 0.0113x + 15.623
R² = 0.0047
Ext PS v TAT (EV)
y = 0.0955x + 0.2032
R² = 0.3829
Ext PE v TAT (EV)
y = 0.4728x + 15.719
R² = 0.0201
Total PS vs D-Dimer (EV)
y = 0.0906x + 4.4303
R² = 0.0888
Total PE vs D-Dimer (EV)
y = 0.2796x + 24.285
R² = 0.0848
Total PS vs TAT (EV)
y = 0.96x + 6.5594
R² = 0.0243
Total PE vs TAT (EV)
y = 5.2658x + 29.755
R² = 0.0734
D-Dimer v Ext PS (Leukocyte - Resting)
y = 3E-06x + 0.499
R² = 8E-08
D-Dimer v Ext PE (Leukocyte - Resting)
y = -0.0007x + 2.0707
R² = 0.0003
TAT v Ext PS (Leukocyte - Resting)
y = 0.0103x + 0.4942
R² = 0.0018
TAT v Ext PE (Leukocyte - Resting)
y = 0.0811x + 2.0125
R² = 0.0117
D-Dimer vs Total PS (Leukocyte - Resting)
y = -0.0143x + 26.542
R² = 0.0003
D-Dimer vs Total PE (Leukocyte - Resting)
y = 0.0109x + 75.061
R² = 3E-05
TAT vs Total PS (Leukocyte - Resting)
y = 2.2468x + 25.06
R² = 0.0209
TAT vs Total PE (Leukocyte - Resting)
y = 6.7922x + 72.124
R² = 0.0329
D-Dimer v Ext PS (Leukocyte - Ionophore)
y = -0.0018x + 3.8544
R² = 0.0012
D-Dimer v Ext PE (Leukocyte - Ionophore)
y = 0.0016x + 14.277
R² = 4E-05
TAT v Ext PS (Leukocyte - Ionophore)
y = -0.048x + 3.8263
R² = 0.0021
TAT v Ext PE (Leukocyte - Ionophore)
y = -0.1464x + 14.392
R² = 0.0009
D-Dimer v Total PS (Leukocyte - Ionophore)
y = 0.0515x + 19.178
R² = 0.0063
D-Dimer v Total PE (Leukocyte - Ionophore)
y = 0.1511x + 82.019
R² = 0.0059
TAT v Total PS (Leukocyte - Ionophore)
y = -0.2889x + 20.787
R² = 0.0005
TAT v Total PE (Leukocyte - Ionophore)
y = 3.7274x + 84.553
R² = 0.0088

Figure S12

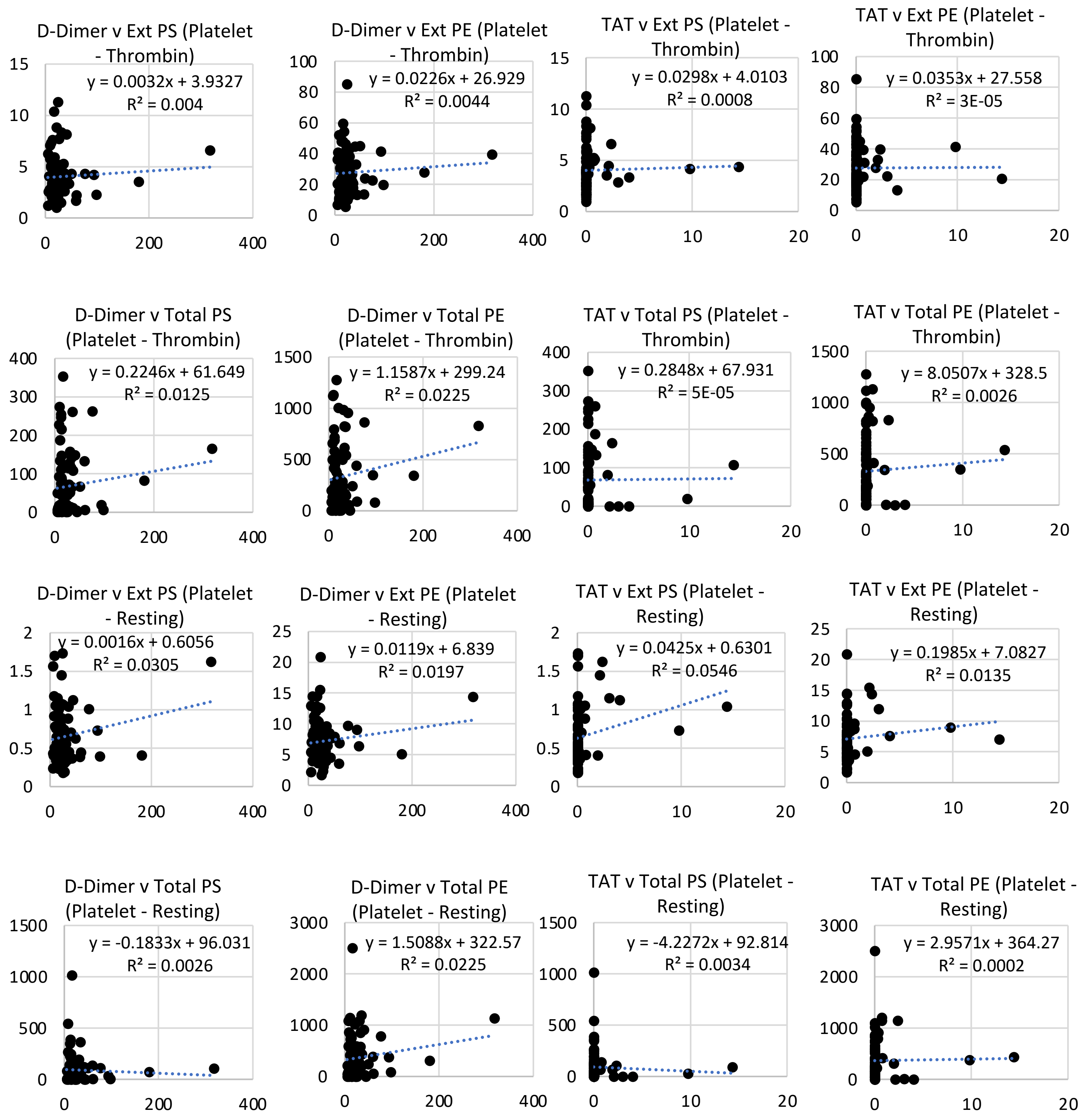
D-Dimer v Ext PS (Platelet - Thrombin)
y = 0.0032x + 3.9327
R² = 0.004
D-Dimer v Ext PE (Platelet - Thrombin)
y = 0.0226x + 26.929
R² = 0.0044
TAT v Ext PS (Platelet - Thrombin)
y = 0.0298x + 4.0103
R² = 0.0008
TAT v Ext PE (Platelet - Thrombin)
y = 0.0353x + 27.558
R² = 3E-05
D-Dimer v Total PS (Platelet - Thrombin)
y = 0.2246x + 61.649
R² = 0.0125
D-Dimer v Total PE (Platelet - Thrombin)
y = 1.1587x + 299.24
R² = 0.0225
TAT v Total PS (Platelet - Thrombin)
y = 0.2848x + 67.931
R² = 5E-05
TAT v Total PE (Platelet - Thrombin)
y = 8.0507x + 328.5
R² = 0.0026
D-Dimer v Ext PS (Platelet - Resting)
y = 0.0016x + 0.6056
R² = 0.0305
D-Dimer v Ext PE (Platelet - Resting)
y = 0.0119x + 6.839
R² = 0.0197
TAT v Ext PS (Platelet - Resting)
y = 0.0425x + 0.6301
R² = 0.0546
TAT v Ext PE (Platelet - Resting)
y = 0.1985x + 7.0827
R² = 0.0135
D-Dimer v Total PS (Platelet - Resting)
y = -0.1833x + 96.031
R² = 0.0026
D-Dimer v Total PE (Platelet - Resting)
y = 1.5088x + 322.57
R² = 0.0225
TAT v Total PS (Platelet - Resting)
y = -4.2272x + 92.814
R² = 0.0034
TAT v Total PE (Platelet - Resting)
y = 2.9571x + 364.27
R² = 0.0002

Figure S13

Major Resources Table

In order to allow validation and replication of experiments, all essential research materials listed in the Methods should be included in the Major Resources Table below. Authors are encouraged to use public repositories for protocols, data, code, and other materials and provide persistent identifiers and/or links to repositories when available. Authors may add or delete rows as needed.

Animals (in vivo studies)

Species	Vendor or Source	Background Strain	Sex	Persistent ID / URL
NA				

Genetically Modified Animals

	Species	Vendor or Source	Background Strain	Other Information	Persistent ID / URL
**Parent - Male**					
**Parent - Female**					

Antibodies

Target antigen	Vendor or Source	Catalog #	Working concentration	Lot # (preferred but not required)	Persistent ID / URL
NA					

DNA/cDNA Clones

Clone Name	Sequence	Source / Repository	Persistent ID / URL
NA			

Cultured Cells

Name	Vendor or Source	Sex (F, M, or unknown)	Persistent ID / URL
NA			

Data & Code Availability

Description	Source / Repository	Persistent ID / URL
NA		

Other

Description	Source / Repository	Persistent ID / URL
NA		

DOI [to be added]

ARRIVE GUIDELINES

The ARRIVE guidelines (https://arriveguidelines.org/) are a checklist of recommendations to improve the reporting of research involving animals. Key elements of the study design should be included below to better enable readers to scrutinize the research adequately, evaluate its methodological rigor, and reproduce the methods or findings.

Study Design

**Groups**	**Sex**	**Age**	**Number (prior to experiment)**	**Number (after termination)**	**Littermates (Yes/No)**	**Other description**
Group 1 (Control)						
Group 2						
Add more if needed						

Sample Size: Please explain how the sample size was decided Please provide details of any a *prior* sample size calculation, if done.

Inclusion Criteria

Exclusion Criteria

Randomization

Blinding

DOI [to be added]
